# Approaching prehistoric demography: proxies, scales and scope of the Cologne Protocol in European contexts

**DOI:** 10.1098/rstb.2019.0714

**Published:** 2020-11-30

**Authors:** Isabell Schmidt, Johanna Hilpert, Inga Kretschmer, Robin Peters, Manuel Broich, Sara Schiesberg, Oliver Vogels, Karl Peter Wendt, Andreas Zimmermann, Andreas Maier

**Affiliations:** 1 Institute for Prehistoric Archaeology, University of Cologne, Cologne, North Rhine Westphalia, Germany; 2 Landesamt für Denkmalpflege im Regierungspräsidium Stuttgart, Esslingen am Neckar, Germany; 3 Landschaftsverband Rheinland, Amt für Bodendenkmalpflege im Rheinland, Endenicher Strasse 133, Bonn, Nordrhein-Westfalen 53115, Germany

**Keywords:** hunter–gatherers, farmers, population size, upscaling, manuals, computational archaeology

## Abstract

In many theories on the social and cultural evolution of human societies, the number and density of people living together in a given time and region is a crucial factor. Because direct data on past demographic developments are lacking, and reliability and validity of demographic proxies require careful evaluation, the topic has been approached from several different directions. This paper provides an introduction to a geostatistical approach for estimating prehistoric population size and density, the so-called Cologne Protocol and discusses underlying theoretical assumptions and upscaling transfer-functions between different spatial scale levels. We describe and compare the specifics for farming and for foraging societies and, using examples, discuss a diachronic series of estimates, covering the population dynamics of roughly 40 kyr of European prehistory. Ethnohistoric accounts, results from other approaches—including absolute (ethno-environmental models) and relative estimates (site-numbers, dates as data, etc.) allow a first positioning of the estimates within this field of research. Future enhancements, applications and testing of the Cologne Protocol are outlined and positioned within the general theoretical and methodological avenues of palaeodemographic research. In addition, we provide manuals for modelling Core Areas in MapInfo, ArcGIS, QGIS/Saga and R.

This article is part of the theme issue ‘Cross-disciplinary approaches to prehistoric demography’.

## 1. Introduction

Prehistoric population size and density are both considered important indicator variables for a society. However, controversy exists as to the nature of the relationship between demography and socio-cultural developments. Demographic configurations can be causal or consequential to social and cultural evolution, or rather constitute one factor acting in interdependence with other influences from outside and within a society. To approach the controversy, reliable and consistently derived long-term demographic models are needed that allow for an analysis of the nature of the relationship through the millennia and across populations with different social and economic organizations. To this end, adequate up- and downscaling of data is required, because both population size and density are related to dynamic processes that can only be evaluated if approached at different temporal and spatial scales. Childe [1] proposed a model on human demographic developments (electronic supplementary material, figure S1). It discerns four epochs, interrupted by fundamental changes in the socio-economic organization of human societies. These ‘revolutions' [1] or ‘great transformations' [2,3] are related to unprecedented increases of population density. During each epoch, oscillating or cyclic changes—also termed ‘booms and busts’ [4] or ‘cultural cycles’ [5,6]—of the population sizes among cooperating groups become apparent. Building onto this, the Cologne Protocol proposes a methodological framework encompassing three aspects: spatially explicit demographic estimates using suitable proxies, scaling of data and bridging between different epochs.

## 2. Proxies and scales in current research frameworks

Early studies on absolute estimates for Palaeolithic periods noted the absence of any ‘records worth considering’ [7, p. 197] and simply assumed a population size on global scales [7,8; 9: fig. 2]. Until today, a main aim of palaeodemography is to demonstrate ‘conclusively that the pattern in the proxy data reflects past demographic change’ [10, p. 156]. To infer demographic developments, the validity, reliability and robustness of the proxy used must be checked. The internal validity, i.e. the extent to which a proxy is related to demography, can vary considerably (table 1): for example, the number of stone tools produced relates less likely to population size than the number of settlement structures does. However, any proxy is affected by biasing factors. It is thus important to consider a given proxy in relation to its spatial relevance and its temporal resolution. To overcome restrictions of proxies, meaningful combinations of proxies as well as a suitable methodological framework for adequate transfer between scales are needed.

**Table 1.** Archaeological, ecological and ethnographic proxies used to infer prehistoric demography. (In most instances, distinct statements for foraging (Fo) and farming (Fa) societies were necessary. Shading indicates whether the aspect has positive (light) or negative (dark) effects for the respective proxy.)

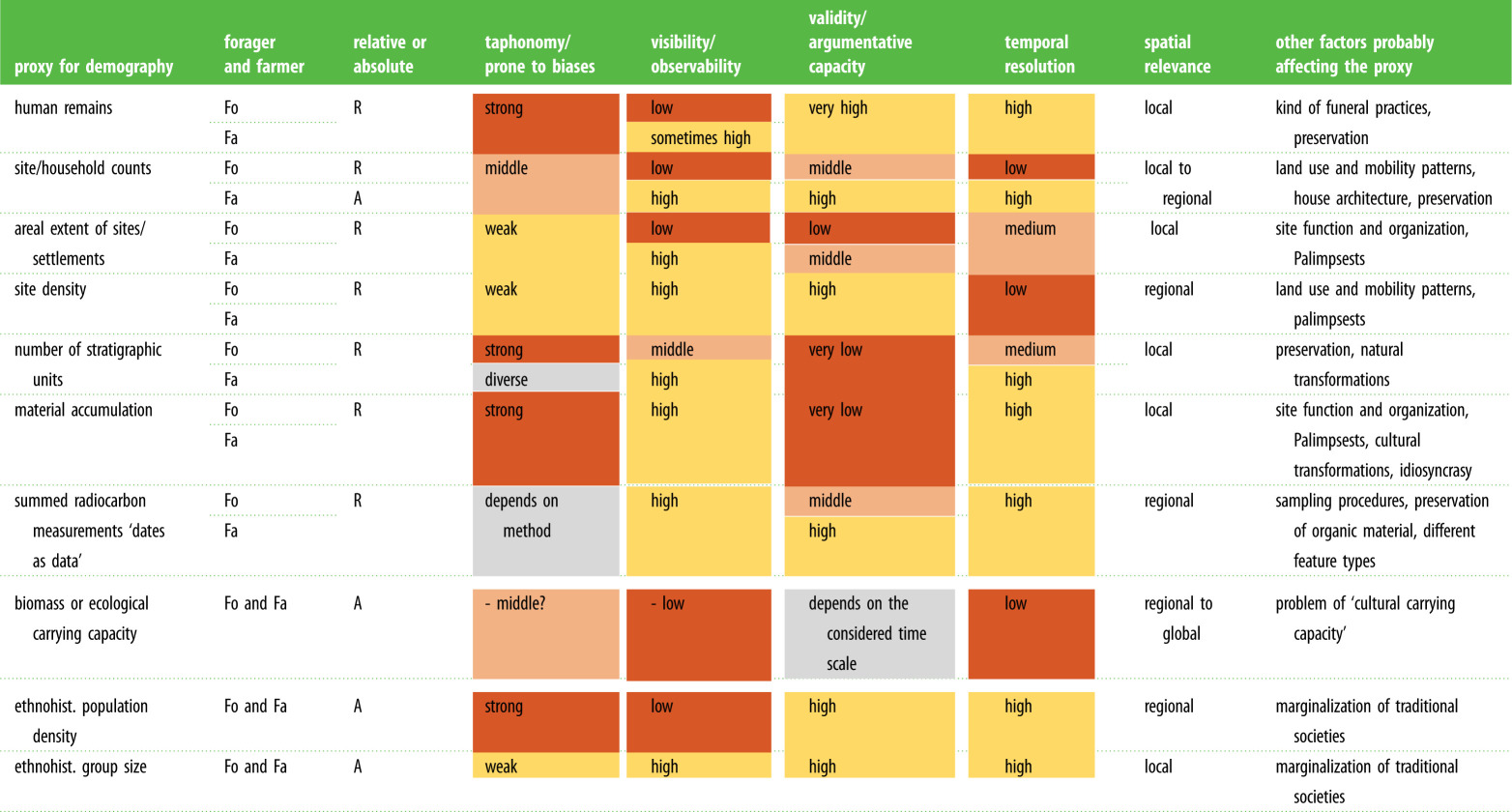

The power of a proxy to provide inferences on demography can gain additional credibility if these inferences are reproducible and consistent within different methodological and interpretative frameworks. A common approach for assessing the robustness of proxy data for prehistoric contexts is through newly discovered evidence, i.e. to consider the derived interpretation in the light of changing data availability through time and see whether it still holds true ([11–13], [14, pp. 304–318]).

Current approaches use proxies to infer demographic trends either as relative population changes or as absolute population sizes similar to census data and densities for prehistoric populations. Information on relative population dynamics is obtained by summed probability radiocarbon plots through the ‘dates as data’ approach [4,15–19], or by using the frequencies of sites and assemblages (e.g. [10–13,20,21]).

Estimates of absolute numbers of individuals for Palaeolithic and Neolithic periods are inferred from ecological, ethnographic and/or archaeological proxies ([22], [10: table 1]). For the European Upper Palaeolithic, population sizes were estimated by calculating the available biomass to assess carrying capacity [23], or by integrating ethno-historically documented population densities into interpolations of extensive areal coverage calculated from changes in site densities of subsequent periods [12,24]. Population estimates computed independently from the archaeological record were proposed by transferring the correlation of ethnohistoric population density and climatic conditions to modelled palaeoclimatic conditions of the Upper Palaeolithic [25]. Population estimates for agrarian societies at a regional scale are usually obtained by comparing hypotheses on nutritional carrying capacity with the observed frequency and size of settlements. Work on burial grounds results typically in a reconstruction of a local population [26].

Within this research landscape, the Cologne Protocol combines different proxies and methods in a spatially explicit, density-based upscaling approach [27–30]. This allows the derivation of population size and density data at different spatial scales, ranging from regional areas of social units up to continents. Proxies are selected based on their validity for the relevant socio-economic setting (mobile hunter–gatherers or sedentary farmers), while the overall methodological framework is kept consistent.

## 3. The Cologne Protocol for different socio-economic contexts

The methodological framework of the Cologne Protocol to estimate population sizes and densities at different spatial scales is outlined in table 2 for foragers and farmers. The procedure consists of two successive tasks: a geographical information system (GIS) analysis of site distribution, identifying so-called Core Areas, followed by a computational upscaling procedure of population data. Descriptions of both tasks are provided in the electronic supplementary material, S1 and S2, including manuals to reproduce the spatial analysis for different GIS software (electronic supplementary material, S1.3). In the following, we focus on the structure of this approach to forager as well as early farming society contexts.

**Table 2.** Summary of the scale levels, methods and parameters of the Cologne Protocol for mobile foragers and sedentary farming communities.

scale levels	foragers	farmers	parameters/methods
Total Area of Calculation (TAC)	diachronic comparability (occupied/empty areas, changes in land use/mobility; push and pull factors)		calculation of large-scale densities, regression map section defined by meaningful eco-cultural units
	extent of archaeological units (technocomplexes)	extent of archaeological units (archaeological cultures)	
Core Area	(Extended Area) interconnected socio-economic area (combining Core Areas and catchment areas)	(Core Area)	Optimally Describing Isolines (ODI) at regional studies
	(Core Area) (persons/group -> economically similar ethnographic data)		
(area of social unit)	annual mobility how many km^2^ per group? median, upper/lower limits of raw material catchment areas	Key Area How many km^2^ per (a) household? (b) burial ground?	Voronoi polygon (farmer)/raw material catchments (hunter–gatherers)
archaeological site	camp site (problem: variability of site function)	(a) dated household (problem: how many persons/household?) (b) dated graves	excavation

### (a) Methods, scale levels and basic assumptions

The first task is the geostatistical modelling of Core Areas. This term is not to be confused with ‘culture core’ or ‘culture area’ as used in cultural anthropology and defined by cultural traits [31, p. 37 and 93f.]. Our Core Areas are geostatistically calculated based on site coordinates by identifying an Optimally Describing Isoline (ODI). The ODI encircles areas with identical site density and delimits them against areas where sites are scarce or absent. We consider the former as intensively occupied core areas and the latter, by contrast, as marginal with minor or no settlement activity.

For the Palaeolithic, estimation periods cover several thousands of years during which areas might shift in and out of the human exploitation range, owing to environmental changes. In order to deal with these oscillations, Core Areas inferred from ODIs provide a means to average the varying extensions of the exploited area and will reduce short-term outliers for the period under research. This makes areal estimation more conservative than approaches considering all single archaeological occurrences with covering hulls (see the electronic supplementary material, S1.1 and 1.4). It is also less prone to distortions by new discoveries, and therefore, results are considered more robust [32]. The approach assumes, firstly, that the distribution of sites across the landscape reflects human occupation during a defined period; secondly, that biases thereof can be identified or controlled through critical evaluation of equivalent data from preceding and succeeding periods in the same region; and thirdly, that a certain level of site density will reflect Core Areas of intense and continuous occupation.

The second task is a mathematical procedure to estimate population sizes for the Core Areas from adequate proxies—a choice based on their validity within the specific socio-economic context—and to transfer data between scales. Through the combination of spatial information (Core Areas/ODI) and demographic information on the level of social units (table 2), the population sizes and densities for a given region are estimated. Simply put, data concerning population densities derived from archaeological and/or ethnographic observations on lower scale levels are transferred to higher levels by an upscaling procedure (see the electronic supplementary material, S1.4).

On the areal scale of a social unit, the houses and settlements of sedentary farmers can be considered to be a representation of a perennially existing socio-economic unit. In this context, the so-called Key Areas are regions where intensive research with extensive coverage warrants a particularly good knowledge of the existing sites. An analysis of the site distribution by Voronoi (Thiessen) polygons delimits the areal extent of settlements in relation to their neighbours. Here, the estimated average space per household or per person is an important result (see the electronic supplementary material, S2.1).

In contrast with settlements of sedentary people, the occupation histories of hunter–gatherer sites vary seasonally and functionally throughout a year. Determination of the area of a specific perennial socio-economic unit, therefore, needs to consider other proxies, e.g. those relating to mobility and networks. To determine the spatial range (territory) of a foraging group, the Cologne Protocol includes data on stone materials which were transported and used for tool production to an economically relevant extent (see the electronic supplementary material, S2.2). Since these data probably reflect the mobility range of a seasonally or even annually aggregating group, the Cologne Protocol uses a mean value of group 2 size [33], optimized for non-mounted hunter–gatherers with predominantly terrestrial resource exploitation (see the electronic supplementary material, S2.2; [29,30]). For foragers, the protocol thus combines information from three different proxies—site density, raw material procurement and ethnographic information—which, in combination, result in regionally differentiated estimates of hunter–gatherer population sizes and densities [30,34–37]. For foragers—contrary to farming societies—evidence indicates that Core Areas are sometimes connected by common raw material use and might have been part of seasonal rounds [30,36]. Therefore, the scale level of Extended Areas (table 2) is introduced to capture regional and supra-regional socio-economic spatial units.

The highest scale level, the Total Area of Calculation (TAC), constitutes the defined map section for the entire calculation. It should relate meaningfully to the archaeological units under study, and also consider the patterns of data biases and environmental factors. Areas known as uninhabitable (e.g. sea surface, ice sheets) should be excluded, as well as areas for which the archaeological record is known to be missing (e.g. submerged coastal areas). Diachronic comparisons take place at the scale level of TAC population densities. No prior calibration of the estimates, e.g. by the length of studied periods, is needed (see also the electronic supplementary material, S2.2.3).

### (b) Population size and density estimates: results for foraging and farming societies

Population estimates were obtained for 14 archaeologically defined periods using the Cologne Protocol, covering a total timespan of roughly 40 kyr and including hunter–gatherer as well as different forms of farming societies. To allow for a positioning of our estimates within the field of palaeodemographic research, we will discuss influencing factors, such as handling of areas devoid of archaeological sites, differing durations of the periods and uncertainties of the estimates. The results are summarized in table 3. Density estimates are given for Core Areas and the TAC. For Palaeolithic periods, TACs are held constant to improve comparability.

**Table 3.** Results of estimates for selected Prehistoric and Historic periods. (The central tendency of estimates is given for the Core Area (CA) population density, the variance in TAC population density is calculated using 1st and 3rd quartile of regionalized raw material polygons (for Palaeolithic) and considering persons per household (HH) or persons per necropolis (Necr., for later Periods).)

period	chronology			Core Area (km^2^)	TAC (km^2^)	HH or Necr.	density of HH/Necr.	sites outside CA	P/Generation/HH or Necropolis			Core Area PopDensity	TAC PopDensity			reference
	start	end	duration						low	central tendency	high		low	mean	high	
Merowingian Period (MP)	530 AD	670 AD	140	9571	60 008	Necr.								0.9		[14: Tab. 37]
(MP) Merowingians				5883.5		Necr.	0.09	147	52.6	61.8	71.1	9				[14: Tab. 27]
(MP) Saxons				3687.5		Necr.	0.02	25	11.3	13.4	15.6	0.4				[14: Tab. 27]
Roman Empire (RE)	150 AD	200 AD	50	9304	22 848	vicus+colonia +soldiers		508				56.9	10.9	14.4	17.9	[38: Tab. 3]; [14: Tab. 37]
RE agarian high				1798		Villa	0.98		10	15	20					[38: Tab. 3]
RE agrarian low				7506		Villa	0.22		25	37.5	50					[38: Tab. 3]
Iron Age (IA)	600 BC	475 BC	125	10 573	23 935							4		1.8		[14: Tab. 37]
IA lowland				2243	3720	HH	0.19	11	5	7.5	10					[14: Tab. 5,6 and 235]
IA Loess area				2651	5772	HH	1.59	15	5	7.5	10					[14: Tab. 10]
IA upland				5679	14 443	Necr.	0.05	36	11.8	14.3	16.8					[14: Tab. 16]
Early Neolithic (Linear Pottery)	5250 BC	5050 BC	200	2261	37 989	HH		398.7	7	8.5	10	8.50	0.5	0.6	0.7	[28: (after: 14: Tab. 32, 37 and 311)]

						*n* group			group size							
	BP					low		high	low		high					
Late Palaeolithic	14 000	11 700	2300	581 067	2 300 000	74	155	257	35	43	57	0.0109	0.0012	0.0025	0.0042	[37]
Final Magdalenian	14 670	14 000	670	295 000	2 300 000	113	179	249	35	43	57	0.0261	0.0021	0.0033	0.0047	[29]
Late Magdalenian	16 500	14 670	1830	144 300	2 300 000	52	85	122	35	43	57	0.0253	0.0010	0.0016	0.0023	[29]
Middle Magdalenian	18 500	16 500	2000	108 200	2 300 000	27	44	80	35	43	57	0.0175	0.0005	0.0008	0.0015	[29]
Early Magdalenian	20 000	18 500	1500	72 600	2 300 000	7	28	53	35	43	57	0.0166	0.0001	0.0005	0.0010	[29]
Last Glacial Maximum	25 000	20 000	5000	275 413	2 300 000	31	76	147	35	43	57	0.0119	0.0006	0.0014	0.0028	[34]
Gravettian Phase 2	29 000	25 000	4000	123 810	2 300 000	16	24	36	35	43	57	0.0082	0.0003	0.0004	0.0007	[35]
Gravettian Phase 1	33 000	29 000	4000	243 039	2 300 000	39	65	85	35	43	57	0.0115	0.0007	0.0012	0.0016	[35]
Aurignacian	42 000	33 000	9000	107 188	2 300 000	21	36	90	35	43	57	0.0146	0.0004	0.0007	0.0017	[36]

**Figure 1.**
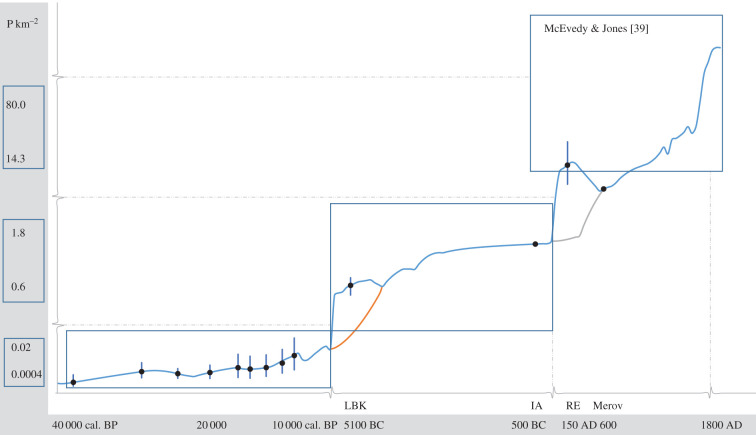
Demographic estimates of the Cologne Protocol, interpolated applying logistic equation. Modelling took place with different time-intervals (*x*-axis: Palaeolithic/Mesolithic = 500 years, Neolithic = 100 years, state societies = 50 years; for parametrization, see the electronic supplementary material, table S6). For Upper Palaeolithic phases, density values (*y*-axis) are inflated by a factor of 60 to make changes perceivable. Bars indicate uncertainties considering quartiles (see §4(b)). Areal scale of the estimates: Pal around 2,3 Mio km^2^; others: 20–40 000 km^2^. LBK, Linearbandkeramik; IA, Iron Age; RE, Roman Empire; Merov, Merovingian. The red line is related to a delay in food producing economy, i.e. Funnel Beaker Culture distribution area compared with the LBK. The grey line reflects the delay in state formation in the area east of the Rhine and north of the Danube in Central Europe.

The modelling of long-term population dynamics (figure 1) is accomplished by interpolating between the obtained estimates using a logistic equation. The mathematical expression is described in detail in Zimmermann *et al*. [3] and builds on considerations of Malthus [40,41] and Verhulst [42,43]:

$$\text{logistic}\;\text{equation}:\;N_{t}=r\;*\;N_{t-1}\;*\;(1-N_{t-1}/(K_{1}+K_{2}+\text{CE})).$$

The equation first assumes population growth (*r*) in a geometrical ratio, which accounts for each generation (*N_t_*) in relation to its predecessor generation (*N_t_*_−1_). The growth factor per generation can be determined empirically, e.g. in the case of early Neolithic farmers, by relating estimates of successive periods, (*r*) = (*N_t_*/*N_t_*_−*n*_)^(1/*n*)^. However, a self-limiting factor to the growth rate needs to be introduced (1 − *N_t_*_−1_). This self-regulation [42,43] finds applications in concepts of ecology or economics, e.g. the ‘law of diminishing returns' [44]. Finally, to interpolate between our estimates from different economic stages, we need to adjust carrying capacity (*K*) for population density, which is distinguished into *K*_1_ = a minimum nutritional carrying capacity, determined by technological/economic capacities and *K*_2_ = *Conjoncture*, i.e. booms and busts as well as trends resulting from positive feedback loops. Finally, catastrophic events (CE) such as the Black Death and the Thirty Years’ War affecting population size must be considered.

We prefer the logistic equation to other alternative interpolations (e.g. spline interpolation) in this context, because it allows maximum control and comparability of each factor and its effects across phases and epochs. Depending on *r* and *K*, a certain delay for succeeding interpolations is introduced so that maxima and minima are located between our estimates. Considering the uncertainties, this seems appropriate.

As such, the Cologne Protocol provides a mathematical interface between different epochs and fills the gap on demographic estimates before any available census data in Europe. To visualize the results, an ‘arithmetic-exponential’ representation with a linear scaling of the population axis is not suitable [45, p. 25 and fig. 2.1a] because the small figures of hunter–gatherers as well as early farmers are not recognizable if the scale has to cover values up to hundreds of persons in modern times. Also, a logarithmic scaling would only be a good solution if just two stages with a dynamic equilibrium would exist. However, owing to important bundles of innovations, we distinguish according to Childe [1], a total of four epochs in figure 1. In such a situation, logarithmic scaling would reduce differences between times with high densities. Therefore, a decomposition in four different scales is appropriate (‘arithmetic-logistic’, see [45, p. 25, and fig. 2.1c]. Parametrization of the logistic equations is reported in the electronic supplementary material, table S6. The resulting picture (figure 1) confirms Child's [1] scenario of ‘revolutions’ with stepwise increases in human population between epochs. This is considered to result from positive feedback loops with economic and social changes of societies as main drivers, which altered previous patterns of fertility and/or mortality and thus possible population densities [3].

## 4. Discussion

Many processes and factors influence the visible structure of the archaeological record of Palaeolithic and Neolithic societies: besides uncertainties in dating and techno-typological systems (e.g. [46]), these are climatic conditions, flora and fauna, soil, raw materials and topography, etc. They cause different socio-environmental interactions on various scales. Up- and downscaling is thus an inevitable component of meaningful prehistoric research [47].

### (a) Mind the gap: dare the gap!

The application of the Cologne protocol generally results in lower population density estimates than results from other approaches to prehistoric populations. One reason is the incorporation of ‘empty areas’ in the calculations and the upscaling procedure, both between Core Areas and—in some instances—within them.

Neither foragers nor farmers move across the landscape randomly, they do not use potentially inhabitable areas entirely, nor exploit those occupied areas up to the limit. As a consequence, and just as like any other species, humans do not distribute themselves evenly across space; they form a pattern of clusters and voids [48]. Gaps in the distribution of archaeological phenomena are thus not necessarily only the result of taphonomic loss and research biases, but may also reflect prehistoric reality. The present approach evaluates occupied as well as potentially empty spaces in the archaeological record individually. We have shown that areas outside as well as in between modelled Core Areas cover potential and ecologically suitable, but yet uninhabited areas [27]. This contrasts with previous demographic approaches—especially for foragers (e.g. [12,25])—as well as traditional mapping of prehistoric cultures (e.g. [49]). Large-scale maps tend to ‘upscale’ local and regional information on site location by assuming a blanket coverage, every occurrence is considered as equally representative for the spatial extent of the entity. We argue that such area-covering conceptions overestimate human presence and population size.

Core Areas and regions with minor or no occupation activities are, therefore, essential features of the Cologne Protocol. Their acceptance requires the adoption of new perspectives. At large spatial scales of investigation, extensive regions of minor or no occupation might have played specific roles in maintaining the observed long-term dynamic equilibrium between Great Transformations, providing sink areas for demographic booms and source areas for sustainable resources, e.g. prey animals. They could also have served the purpose of ‘social demarcation’ [28], or been related to other aspects of socio-spatial organization among forager societies. Such social needs can be expressed in a cultural carrying capacity [36,50], which might have become even more pronounced in later times.

The existence of areas of no or low settlement activities, regardless of their potential suitability, can be confirmed for Holocene periods. The use of ODI provides a formal procedure for source criticism. To address the validity of empty areas, changes of Core Areas in a specific region can be analysed more closely. As an example, in a case study from the Rhineland [27,28], the superimposition of ODI's from different time horizons made it very unlikely that obvious gaps between Core Areas resulted from erosion or missing archaeological observation, because many other periods were represented in the critical area. This leads to the conclusion that this empty, uninhabited space existed in the past, and that the landscape was not used to its full nutritional carrying capacity.

This is also reflected in the analysis of land suitability and use [27, pp. 21–32]. The culturally differing preferences regarding soil and precipitation were geostatistically defined using ODIs and compared with their degree of exploitation by different archaeological cultures. Again, the existence of large unused regions with apparently suitable conditions in prehistoric times can be confirmed. Additionally, land use analysis indicates that the land within Core Areas was not used to its full capacity either. To better understand the heuristic value of the Core Areas in terms of subsistence and social organization, a case study has been undertaken for farming communities, made on an unbiased, nearly complete archaeological record from a lignite mining area in Germany [51–53]. The areal requirements to satisfy human's demand for nutrition and energy were calculated based on the archaeologically documented economic structure of early Neolithic societies. The results are given in hectares (ha) per person (table 4), showing that 2.35 ha of agrarian area was required to feed a person (considering only domesticated plants and animals). This value differs considerably from the available space modelled for Core Areas (11.8 ha person^−1^). The same pattern of much larger Core Areas than were required from an economic perspective was observed for Iron Age societies. If the Core Areas were not entirely used for agrarian needs, what was their purpose? They were potentially exploitable, but not used to their full capacity for low-intensity activities such as collecting and hunting.

**Table 4.** Comparison of required and available space (hectare per person) for early Neolithic (LBK) and Iron Age societies (taken from: Wendt *et al*. [51]). (Agrarian areas were calculated based on humans' nutritional and energy requirements; numbers in bold are the intermediate sum of the values above; values modelled in the framework of the Cologne Protocol are written in italics.)

types of land use	hectare per person				
	Neolithic	Iron Age (IA)			
	LBK	total	upland	loess soils	lowland
pasture	2.0	1.7	1.7	1.7	1.7
garden, fields	0.3	0.4	0.4	0.4	0.4
yard and buildings	0.05	0.05	0.05	0.05	0.05
intermediate sum of agrarian areas	**2.35**	**2.15**	**2.15**	**2.15**	**2.15**
*max. space available in Core Area*	*11.8*	*25.0*	*125.0*	*8.4*	*45.5*
low-intensity activities	8.9	22.9	122.9	6.3	43.4

estimated densities	persons per km^2^				
*Core Area population density*	*8.5*	*4.0*	*0.8*	*5.5*	*1.4*
*TAC population density*	*0.6*	*1.8*	*0.3*	*5.5*	*1.4*

### (b) Uncertainties of density estimates

Archaeologists are familiar with uncertainties, e.g. for ^14^C-dates, where the mean date represents a time near to the true age. Values above and below 1 s.d. (i.e. beyond 68.3% probability of all possible interpretations of the date) are often excluded from consideration, because the probability that observations at this distance from the mean belong to another context is high. In statistical terminology: with increasing distance from the mean, a probability of a type II error becomes larger.

Unlike ^14^C-dates which have just one variable (proportion of ^14^C), our population estimates must consider three variables and their associated uncertainties: (i) size of Core Area, (ii) number of forager groups, houses or necropolis, and (iii) number of persons per group, household or necropolis. In an earlier study, the size of Core Areas was quite robust, but the number of persons per household was more important [14, pp. 305–308]. Therefore, for farming societies, the number of persons per household or necropolis and for foraging populations, the number of groups seem to be variables with conspicuous variability. The result is a central estimation of highest probability around the mean value and a range between the 1st and 3rd quartile, where values could be located, but with lower probabilities. The range covers 50% of possible population densities owing to the calculation of quartiles.

A question arises as to what to do when the bandwidth of another critical variable is considered. In the case of population estimations for the Upper Paleolithic, introducing group size derived from ethnographic observations is a problem of this kind. By combining variables in the calculation formula of population density (see the electronic supplementary material, S2.2), the range of uncertainty increases with every new bandwidth variable. Working with the median as well as lower and upper quartiles, two variables will result in nine combinations of values (table 5). The probability that low group size is combined with low number of groups or high group size with high number of groups adds to 22% (2 cases out of 9). If these most extreme combinations are excluded, all other possible combinations are located between the 1st and 3rd quartile of estimations based on the number of groups (see the electronic supplementary material, S2.2). Thus, considering only bandwidth of group size would result in a less conservative and reduced range of possible values.

**Table 5.** Calculation of uncertainty in density estimates for forager societies when combining the 1st, 2nd and 3rd quartiles (*Q*1, median, *Q*3) of the number of groups and the group size (here, we present an example for the Early Upper Palaeolithic, cf. table 3). (Numbers in italics, indicate the median; numbers in bold indicate the most extreme combination of variables.)

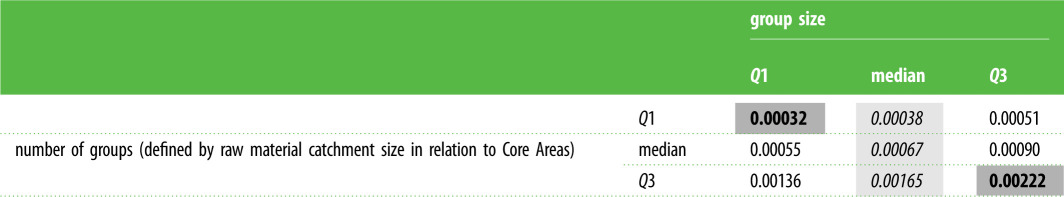

Uncertainties of low estimates for the Upper Palaeolithic calculated in this way often arrive at only half of the central tendency figure and high estimates are in many cases three-quarters larger. These bandwidths are much larger than the ones obtained for early Neolithic and Roman times within a 20 and 25% margin, a margin often deemed acceptable in historical demography [39]. Nevertheless, comparing all estimates, including the Upper Palaeolithic, to each other, these uncertainties do not alter the general tendency of the conclusion (figure 1).

### (c) Oscillations and equilibria of population

At a small spatial scale and with high chronological resolution—as in the case of the early Neolithic in the Rhineland—a boom and bust pattern is observed during the second half of the sixth Millennium BC. Complete excavated settlements start with four contemporaneous houses. After six generations, settlements grow to a maximum of 7–10 houses. This size is maintained for another century and then within two or three generations settlements are abandoned [28: fig. 6]. A quite similar pattern is observed for the Rhineland with about 38 000 km^2^ [3]. While the population estimation with 0.6 P km^2^ for TAC describes the century of maximum density, the densities for growing and declining populations are considerably smaller. A similar oscillation during the fourth Millennium BC is known for the dendrochronologically dated settlements of the Lake Constance area [6]. Considering, however, areas of around 150 000 km^2^ in western and central Europe, these oscillations are only of minor importance.

**Figure 2.**
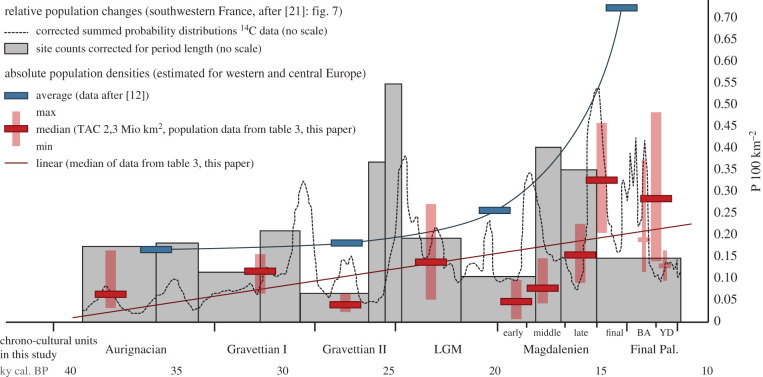
Demographic data for the Upper Palaeolithic in Europe. Comparing relative population changes at regional scale level of southwestern France (without scale, after [21], fig. 7) and density estimates for Europe (mean values, blue bars, after [12]) and TAC population density estimates by the Cologne Protocol (1st, 2nd and 3rd quartiles, red bars, see data and references in table 3), density estimates at scale given as persons per 100 km^2^.

Therefore, the European Upper Palaeolithic estimates almost reach an ‘equilibrium state’ at the TAC scale, at which detectable dynamics are low. Strong fluctuations in population size could have had taken place, especially if the entire bandwidth of our estimates is taken into consideration (table 2), but direct evidence of regions being abandoned or occupied less intensively becomes traceable only at regional spatial scales (see detailed studies by the authors in [29,30,34–37]). The general tendency of increase during the Upper Palaeolithic noted before [12] is confirmed by our estimates (figure 2). Nevertheless, demographic ups and downs do occur and approximately follow environmental conditions. Admittedly, the technocomplexes of the Aurignacian and Gravettian together form one and the same oscillation [35,36]. The underlying mechanisms of these long-term developments are still subject to debate (see [54]).

Surprisingly consistent demographic developments across scales have been observed when comparing relative data on population dynamics for the Upper Palaeolithic of southwestern France [10,21] with our Pan-European results using the Cologne Protocol. The dynamics of the independent proxy data correlate very well (figure 2). The synchronism, especially for low estimates of the later Gravettian, early Magdalenian and Late Palaeolithic, suggests that southwestern France was an important pacemaker in terms of population dynamics in western and central Europe. Discrepancies, offsets or potential delays in the developments might result from the assignment of data with different temporal resolutions. They could also point to phases where southwestern France functioned as a refugium (at the end of Gravettian Phase II; Bölling/Alleröd) or source area for expansions (during the Last Glacial Maximum (LGM) and the Middle Magdalenian).

## 5. Future avenues in modelling population estimates

Research on demography by applying the Cologne Protocol has provided several arguments in favour of Childe's [1] model on past population dynamics (electronic supplementary material, figure S1; figure 1). It has raised questions on adequate scales of analysis, enriched by several case studies for the last 40 000 years. The ability to detect dynamics in population size and density requires a nested scale model that integrates smaller scales into an archaeological long-term perspective and vice versa. Against this background, we propose an agenda of three interlinked fields for future research.

Firstly, methodologically developing the Cologne Protocol for its application to other prehistoric/economic contexts is possible. Besides the shared GIS base measurement of Core Areas, the current calculation procedure for forager societies involves certain proxies, i.e. the area being used by a specific group, and the group size [29,30,55]. A quest for alternative proxies is open here (see [56]). Additionally, concerning group size, ethnographic data show a high variability in relation to economic and environmental factors [33,57,58]. Archaeologically informed models reflecting this variability could expand the applicability of the Cologne Protocol.

Secondly, the availability of higher resolution data on relative and absolute demographic developments now allows a better (re)integration of results into other studies of prehistory and related disciplines, e.g. genetics. Existing reservations towards results from large-scale studies probably rest on scepticism concerning generalizations, which might overwhelm local or regional signals. Increasing the resolution of time series in the reconstruction of dynamic processes can be one solution. In the same way, the available scale levels (i.e. Core Areas, Extended Areas) now allow better integration of high-resolution archaeological spatial data into larger frameworks. Combining regionally working approaches, such as, for example, ‘dates as data’, refine the onset and duration of occupation histories within Core Areas. This agenda point goes along with an improvement in our understanding of the relevance and the spatio-temporal scales of our proxies and related factors.

Finally, we see the needs to address and integrate the existence of ‘empty areas’ in demographic research. Its consideration within the Cologne Protocol is certainly a factor why our estimates on population size and population density are lower in comparison to results collated from the literature (e.g. [28: fig. 8]; [22,26,59]). A basic necessity is to gain a better understanding of scale dependency of population density estimations by cross-cultural comparisons [60]. Moreover, ‘empty areas’ need to be conceptualized and explained within models on population dynamics. Are they culturally and socially loaded, reflecting areas of social demarcation or socially embedded spatial organization, as suggested for the early Upper Palaeolithic [28,36]? If individual or collective decisions—according to Malthus called ‘preventing checks’ in family planning—led to lower population densities than potentially economically sustainable, this would reinforce the introduction of a ‘cultural carrying capacity’ to demographic studies. Differences between nutritional carrying capacity and cultural carrying capacity could be used as one component to measure ‘Quality of Life’ [61]. The archaeological material culture provides evidence for a high diversity of activities not directly oriented to food production, ranging from art and adornments in some periods of the Upper Palaeolithic to megalithic tombs and communal architecture during the Neolithic. This evidence indicates that people also satisfied basic needs other than nutrition and reproduction [3,50].

## Supplementary Material

The Cologne Protocol - 1) Modelling Core Areas and 2) Estimating population sizes and densities

## Supplementary Material

Manual S01: Manual and example application to model ‘Core-Areas’ (Optimally Describing Isolines) using MapInfo & Vertical Mapper

## Supplementary Material

Manual S02: Manual and example application to model ‘Core-Areas’ (Optimally Describing Isolines) using ArcGIS

## Supplementary Material

Manual S03: Manual and example application to model ‘Core-Areas’ (Optimally Describing Isolines) using QGIS/SAGA

## Supplementary Material

Manual S04: Script to model ‘Core-Areas’ (Optimally Describing Isolines) in R

## Data accessibility

All manuals are available in the electronic supplementary material and for updated versions, we refer to https://github.com/C-C-A-A. The example dataset is available at https://crc806db.unikoeln.de/start/.
