# Supplementary material for: Approaching prehistoric demography: proxies, scales and scope of the Cologne Protocol in European contexts
Source: Philos Trans R Soc Lond B Biol Sci. 2020 Nov 30;376(1816):20190714. doi: 10.1098/rstb.2019.0714 (PMC7741091; doi:10.1098/rstb.2019.0714)
Supplement: The Cologne Protocol - 1) Modelling Core Areas and 2) Estimating population sizes and densities [file rstb20190714supp1.pdf]

# Supplementary Information

For the article

## Approaching Prehistoric Demography: Proxies, Scales and Scope of the Cologne Protocol in European contexts

Schmidt, Isabell (*); Hilpert, Johanna; Kretschmer, Inga; Peters, Robin; Broich, Manuel; Schiesberg, Sara; Vogels, Oliver; Wendt, Karl Peter; Zimmermann, Andreas; Maier, Andreas

* corresponding author: isabell.schmidt@uni-koeln.de

The first of two successive tasks within the Cologne Protocol consists of a GIS-analysis of site distribution, identifying so-called 'Core Areas'. The procedures are similar for both foraging and farming societies and are therefore described together (**S1.**), including an overview on available datasets (**S1.2.**) and manuals for different GIS software (**S1.3.**). For a schematic overview we refer to published diagrams for foragers (Schmidt & Zimmermann 2019: Fig S1) and for farmers (Zimmermann et al. 2009a: Fig. 1).

The second task of the Cologne Protocol is the estimation and the upscaling of population sizes and densities (**S2.**). Basic assumptions and parameters differ between foraging and farming societies and are therefore given separately in **S2.1.** and **S2.2**.

A rough schematic overview on the entire approach of the Cologne Protocol is shown below, synthesised from illustrations published for foragers (Maier 2017: Fig 1), for farmers (Zimmermann et al. 2009a: Fig. 1) and information in **Table 2** (this paper).

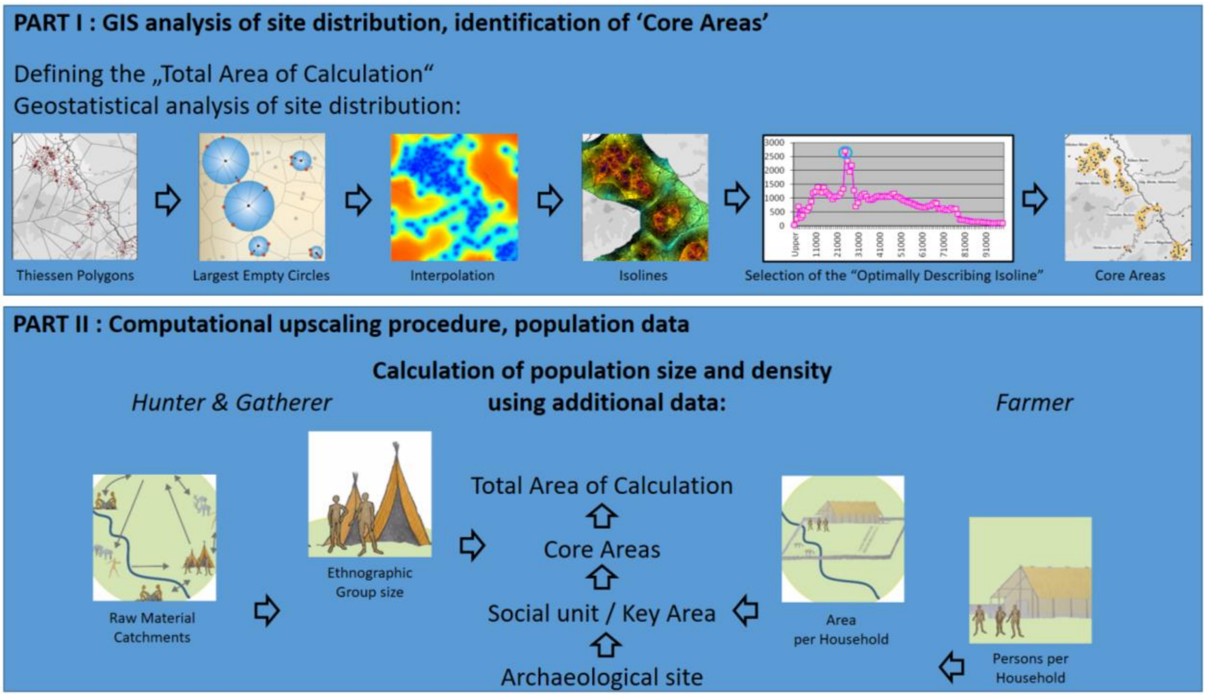


Schematic overview on the Cologne Protocol.

# Content of Supplementary Information

1. Modelling Core Areas - the Optimally Describing Isoline ..... 5
1.1. Dataset requirements ..... 5
1.2. Available datasets ..... 6
1.3. Available manuals ..... 6
1.4. The geostatistical procedure to model Core Areas ..... 8
2. Estimating population sizes and densities ..... 11
2.1. Calculation and underlying theoretical assumptions for sedentary societies ..... 11
2.2. Calculation and underlying theoretical assumptions for foragers ..... 12
2.2.1. From raw material acquisition to the number of groups per Core Areas ..... 12
2.2.2. From numbers of groups to absolute estimates of densities and people ..... 14
3. References ..... 18

**Supplementary Figures**

Figure S1: Nested Model ..... 4
Figure S2: Schematic illustration of effects of forager’s mobility patterns ..... 12

**Supplementary Tables**

Table S1: Concordance table ..... 3
Table S2: Overview of available manuals ..... 7
Table S3: Default values applied for Kriging ..... 9
Table S4: Schematic illustration of calculation ..... 14
Table S5: Ethnographic data ..... 15
Table S6: Parameterisation of Fig. 1 ..... 17

**Table S1**: Concordance table on main terminology and definitions of the Cologne Protocol (Zimmermann et al. 2009a & 2009b, Maier et al. 2016, Schmidt & Zimmermann 2019)

| Term | Description / Definition | Previous nomenclature |
|---|---|---|
| **Cologne Protocol** | Density-based upscaling procedure to estimate palaeodemographic values | "Kölner Methode"; "Cologne Geostatistical Protocol" |
| **Key Area** | Areas with excellent state of archaeological knowledge. In Neolithic contexts used to calculate density values which are transfer to the CA | |
| **Core Area (CA)** | Areas of intensive settlement activities, circumscribed by an Optimally Describing Isoline | Settlement Area, Area of economic interest, Local Scale |
| **Voronoi diagram** | Method to calculate an area belonging to a site depending on the site distribution (not applied to forager contexts) | Thiessen Polygon |
| **Extended Area** | Core Areas extended or linked by Raw Material Catchments | |
| **Total Area of Calculation (TAC)** | Meaningfully selected boundary for the area of investigation | Map Section, Global Scale, Target Scale |
| **Core Area population densities** | Density of people within CA | Local densities |
| **TAC population densities** | Density of people within TAC | Global densities |
| **Isoline** | Encircles areas of equal interpolated site densities | |
| **Optimally Describing Isoline (ODI)** | Encircles Core Areas, selected by different heuristic criteria (increase of area, percentage of site numbers) | Optimal Isoline |
| **Raw material catchment** | Area of raw material procurement per site | Raw material polygon |
| **Bounding box** | Minimum bounding geometry enclosing all voronoi vertices. The bounding box diagonal is used to compute the Lag distance and maximum search distance needed for Kriging. | |
| **Vertex/Vertices** | Points where the voronoi diagrams meet. Each vertex is the centre point of a LEC. | Node/Nodes, "corner point/points" |

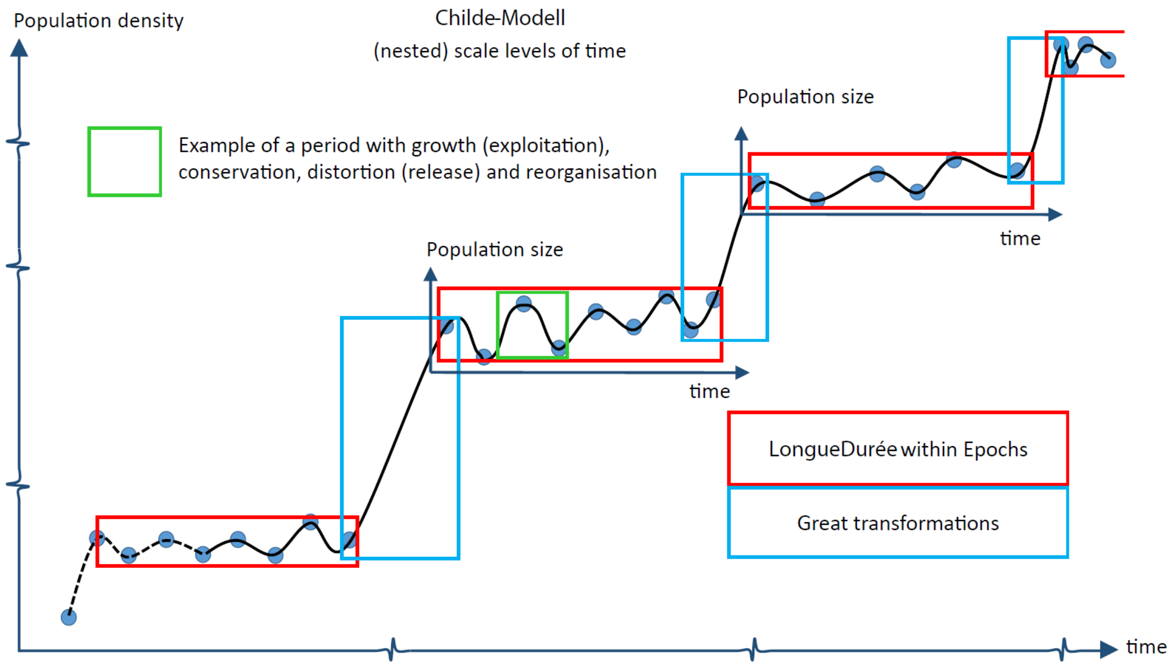


**Figure S1**: Nested model on long-term, large-scale development of population density and size.

# 1. Modelling Core Areas - the Optimally Describing Isoline

Today, there is a wide field of GIS-based methods to approach spatial point patterning in archaeological contexts (Bevan & Conolly 2006; Conolly & Lake 2006: 162–186) e.g. to determine degree and statistical significance of clustering at different spatial scales in a data set by *Ripley's K function* (Ripley 1976; Conolly & Lake 2006: 166–168). Generally, the determination of site clusters is processed in several steps. First, it should be determined whether a clustering trend exists in a given set of archaeological find locations (sites). Second, the attribution of the individual sites to individual clusters (cluster delineation) has to be computed, and third, a statistical procedure should be applied to evaluate some kind of goodness-of-fit for the resulting clusters. Several statistical approaches and GIS-tools are available to compute each of these consecutive steps. Within the workflow of the Cologne Protocol, the second part will be the focus of discussion, i.e. the delineation of site clusters. Often, clustering techniques are used to delineate site clusters (Conolly & Lake 2006: 168–173), or Kernel-Density estimates (ibid. 175–176). Technically, the Cologne Protocol is based on the Largest-Empty-Circle problem (Toussaint 1983), an approach that is rarely considered but well suitable for the delineation of site clusters and hence will be discussed in this section in greater detail.

## 1.1. Dataset requirements

Working with the Cologne Protocol requires access to a GIS and a set of sites with verified georeferenced spatial coordinates and a sound chronological attribution via absolute dating and/or typological assignment. For mobile forager societies, additional information pertaining to lithic raw material acquisition is needed. If direct information on raw materials is lacking, corresponding findings from similar chronological, spatial and environmental contexts (preferably from contemporaneous sites in neighbouring areas) can be used instead.

The selected map section, the Total Area of Calculation (TAC), ideally comprises regions with a similarly well documented and preserved archaeological record. The relation between the size of the TAC and the number of sites it comprises, however, seems to be less important.

There is no clear minimum number of sites, but even studies at smaller spatial scales should consider a minimum of 40-50 sites as a rule of thumb. More important than the mere number of sites is their spatial distribution. Generally, a lower site density will result in a larger Largest Empty Circle (LEC) radius, while a higher site density will result in a smaller LEC radius. If sites are highly clustered, fewer sites are needed to produce robust Optimally Describing Isolines (ODIs). If sites are rather equally spaced in their distribution, more sites are needed to provide a meaningful output. Strongly contrasting site densities in different regions of an area of investigation will result in less robust Core Areas. In these cases, we recommend dividing the area of investigation into regions of high and low site density calculating ODIs for each region independently (see for instance Maier & Zimmermann 2017).

In any case, the final selection of the ODI remains an individual decision, although with heuristic guidance (see below). It is thus necessary that researchers working with the Cologne Protocol have a good knowledge of their study's specific chrono-cultural context (Zimmermann et al. 2009a, 358-360; Kretschmer 2015; Maier et al. 2016).

Some specific configurations, such as linear distributions along rivers or coastlines can produce distortions in the form of edge-effects during triangulation, leading to highly irregular ODI shapes and eventually to demographically meaningless protrusions of the Core Areas into their surrounding space. To identify such spurious results, we recommend a comparison of the ODI shapes with a variance plot of the interpolated LEC radii (see Step 8 below and/or in the manuals). Caution is also advised for areas with high relief, since landscape is treated as two dimensional during the application of tessellation.

## 1.2. Available datasets

To conduct estimations of population density by the Cologne Protocol for foragers and farmers, we used existing data from the map-sets "Geschichtlicher Atlas der Rheinlande" (mentioned below) and "Das Neolithikum in Mitteleuropa" (Preuss 1988) as well as a sample of published radiocarbon dates (Vermeersch 2019).

The point-data (georeferenced archaeological sites) used as an example in all manuals of this Supplementary Information are available at:

https://crc806db.uni-koeln.de/dataset/show/distribution-maps-of-early-neolithic-in-central-europe-and-in-rhineland1559904469/

The CRC 806-website provides most of the original point-data (archaeological sites) and the derived isolines of the CRC- and Rhine-LUCIFS-research except the coordinates of the map-set "Geschichtlicher Atlas der Rheinlande" (Cüppers & Rüger 1985; Joachim 1997; Nieveler 2006; Richter 1997), because the access to the high precision location of archaeological sites within this dataset is limited due to protection reasons. Thus only isolines are available from this data-set and will be available for download at https://crc806db.uni-koeln.de/start/.

For Palaeolithic periods, data were compiled in openly accessible databases for each defined period:

| Period | Reference | Dataset |
|---|---|---|
| Late Palaeolithic | Schmidt & Zimmermann 2019 | (Schmidt & Zimmermann 2020) 10.5880/SFB806.51 |
| Magdalenian | Kretschmer 2015 | (Kretschmer 2015) 10.5880/SFB806.50 |
| Last Glacial Maximum | Maier et al. 2016 | (Maier & Zimmermann 2015) 10.5880/SFB806.50 |
| Gravettian P1 & P2 | Maier & Zimmermann 2017 | (Maier & Zimmermann 2016) 10.5880/SFB806.18 |
| Aurignacian | Schmidt & Zimmermann 2019 | (Schmidt & Zimmermann 2018) 10.5880/SFB806.42 |

## 1.3. Available manuals

The calculation of ODIs and subsequent delineation of Core Areas represents a crucial task in the Cologne Protocol. A number of consecutive steps should be comparable and reproducible within and between GIS software (see **Table S2**). For the technical procedures, please consult the manuals.

**Table S2**: Overview of available manuals for different GIS programs related to working steps in the calculation procedure. The numbering of the working steps corresponds to the numbering of all manuals presented in this study.

| **Working step** | | **Cologne** | | | | **Tübingen** | **Kiel** |
|---|---|---|---|---|---|---|---|
| | | *This publication* | | | | | |
| | **Program** | **MapInfo 8.5** | **ArcGIS X.** | **QGIS 3.10 /SAGA 2.3.1** | R 3.6.2. | **R** | **R** |
| **1.** | **Shape-Layer with sites as points** | MapInfo | ArcGIS | QGIS | R | R | (R) |
| **2.** | **Creating Voronoi polygons** | MapInfo | ArcGIS | QGIS | R | R | (R) |
| **3.** | **Extraction of vertices** | MapInfo | ArcGIS (no aggregation?) | QGIS | R | R | |
| **4.** | **Aggregation of vertices** | MapInfo | ArcGIS? | QGIS | R | R | |
| **5.** | **Defining the radius of the „Largest Empty Circle“** | MapInfo | ArcGIS | QGIS | R | R | (R) |
| **6.** | **Kriging – Preparations and Grid** | MapInfo | ArcGIS | QGIS/SAGA | R | R | |
| **7.** | **Kriging - Semivariogram** | MapInfo<br><br>Manual variogram fitting is possible | ArcGIS<br><br>Manual variogram fitting is possible | SAGA<br><br>Manual variogram fitting is possible | R<br><br>Manual variogram fitting is possible | R<br><br>Manual variogr. fitting is not poss. | R<br><br>Manual variogram fitting is not possible |
| **8.** | **Kriging - inspect and export raster output** | MapInfo | ArcGIS | SAGA | R | R | |
| **9.** | **Creating contour lines (isolines)** | MapInfo regions (polygons) are possible | ArcGIS | SAGA only lines; regions have to been built in a 2. step | R | R | R |
| **10.** | **Calculating the area and the number of sites per isoline** | MapInfo | ArcGIS | SAGA | R | R (just contour lines) | R |
| **11.** | **Data export** | MapInfo | ArcGIS | SAGA | R | R | R |
| **12.** | **Selecting the "Optimally Describing Isoline"** | Excel | Excel | Excel | R | n.a. | R |
| | | | | | | | |
| | **Author(s)** | K. P. Wendt | O. Vogels | R. Peters | R. Peters & M. Broich | Ahlrichs et al. 2016 | ISAAK |
| | **GitHub Link** | https://github.com/C-C-A-A/CologneProtocol-MapInfo | https://github.com/C-C-A-A/CologneProtocol-ArcGIS | https://github.com/C-C-A-A/CologneProtocol-QGIS | https://github.com/C-C-A-A/CologneProtocol-R | | https://github.com/ISAAKiel/lecAAR |

## 1.4. The geostatistical procedure to model Core Areas

The description follows the numbering of steps provided in **Table S2**.

**Step 1. Data preparation – Clearing of site doublets, layer with sites as points**

Sites have a certain spatial extent. Surface collections and find reports of different collectors from directly neighbouring find spots are sometimes listed under different site names. Excavations often uncover only parts of these sites. In some cases the same site is excavated in different, non-overlapping trenches. Some of these also might end up as different entries in registries and databases or reveal different chrono-stratigraphic successions. Since site density is a fundamental factor of the Cologne Protocol, multiple counting of the same site must be avoided. In a first step, closely neighbouring find localities are therefore combined into a single site. Site extent differs between Palaeolithic camp sites and Neolithic or Iron Age settlements, for instance, thresholds of proximity differ between periods. For the Palaeolithic, find spots with a site distance of ≤ 100 m are combined, i.e. aggregated. For the Neolithic this threshold is used as well, but nowadays alternatively 200 m are discussed (this distance is less if a river bisects two settlements, see also Claßen 2012: 18ff, 136ff). The same value (200 m) is used to aggregate the "wandering homesteads" of the Iron Age in the loess area of the Rhineland (Wendt et al. 2010: 237). In the Merovingian period the distance between settlement and cemeteries belonging to one unit as base for the following calculations is set to 500 m (Wendt et al. 2010: 273).

After the data set has been cleared of potential doublets, the sites' coordinates are loaded into a GIS application. It is important to use an area preserving map projection, since area sizes are essential during later steps of the procedure.

**Step 2. to Step 5. The distance measure: The Largest Empty Circle (LECs)**

In the Cologne Protocol, we use the Largest Empty Circle (LEC) between sites as a negative measure of site density. The LEC calculation transforms densities of point data (i.e. sites) into a fielded variable (LECs) via Voronoi (Thiessen) Polygons as an intermediate step (Step 2). Preparata & Shamos (1988, 256; *cf*. Fortin & Dale 2005, 62 fig. 2.18) showed that the vertices (singular: vertex, also known as "corner points" or nodes) of Voronoi Polygons constructed around sites (also referred to as Voronoi diagram, *cf.* O'Sullivan & Unwin 2002: 126-128; Illian et al. 2008: 46-49) represent the centres of LECs encircling the space between the three sites closest to each vertex. Most GIS packages offer a function to extract the vertices from mapped Voronoi polygons (Step 3). However, some functions treat every Voronoi Polygon as an independent object during the extraction of vertices, eventually producing doublets or triplets. Since vertices serve as centres for the LECs, multiple vertices need to be aggregated so that only a single vertex remains for each vertex location (Step 4).

Having cleaned the dataset, the radii of the LECs are calculated around each vertex (see manuals for details in different GIS). The radii serve as a measure of site density at each vertex point and are added as information to the vertex's location in the GIS (Step 5).

**Step 6. to Step 8. From points to areas: interpolation with general Kriging**

Having obtained LEC radii (distances) for each vertex, these point data have to be prepared to be up-scaled to a continuous areal value (Step 6). In the next step, densities of the LEC vertices are interpolated using a certain version of the Kriging methodology (Webster & Oliver 2009). Since the vertex location along with their distance to the next three sites (the LEC radii-values) reflect the density of archaeological sites in a given area, their spatial distribution can be expected to be inhomogeneous and thus to display some large-scale trends (Baddeley et al. 2016, 158; O'Sullivan & Unwin 2002, 65). The Cologne Protocol uses ordinary kriging (Webster & Oliver 2009: 154 and 196 passim), since it does not rely on a steady mean (simple kriging), but tolerates large scale trends and inhomogeneous distributions. To ensure a unified procedure, all interpolations are conducted applying the same preconditions (**Table S3**) and manually fitting a semivariogram model, choosing range and sill according to the first plateau of the empirical variogram (Step 7; Zimmermann et al. 2004: 52). It is an open question in which cases other than a spherical variogram model would provide a better option.

**Table S3**: Default values applied for Kriging.

| | |
|---|---|
| **Lag Distance** | bounding box diagonal / 250 |
| **Variogram Model** | spherical (MapInfo, ArcGIS, R), exponential or power model (QGIS/SAGA) |
| **Maximum Search Distance** | bounding box diagonal / 2 |
| **Minimum Number of Points** | 3 |
| **Maximum Number of Points** | 10 |

An alternative way of transferring point data to areal data is, for instance, Kernel Density Estimation (KDE; e.g. Bowman & Azzalini 1997). Selecting optimised scalar (e. g. Baddeley et al. 2016, 171) or matrix bandwidths (Duong 2016) or even identifying significant density clusters (Duong & Wand 2015; Duong et al. 2008) is not a problem anymore for KDE (*cf*. Herzog 2010; Wendt et al. 2010: 305).

However, for the Cologne Protocol, we prefer the procedure outlined above. The reason for this is archaeological rather than mathematical or conceptual. Because KDE results are quite susceptible to local increases in site numbers, they are rather sensitive to the steady increase in archaeological knowledge and research. The results of the LEC approach are, in contrast, quite robust towards this effect (Zimmermann et al. 2004). LEC results will start to vary notably, as soon as archaeological research discovers densely settled regions which had previously been unknown and whose spatial extents equal those of known site clusters. But given the advanced state of research, this seems rather unlikely for most regions in Europe.

The resulting raster map of the interpolated LEC values provides a visual impression of the differences in site densities within the area of observation. Regions with small LEC values, i.e. small distances between the sites, signal densely occupied map sections, while regions with large LEC values mark sparsely occupied regions. Most GIS software provide a raster map showing the variance or standard deviation as quality measure of the interpolation. Low variance/standard deviation marks a reliable estimate, high variance/standard deviation a higher degree of uncertainty (Step 8).

**Step 9. to Step 12. Computing and selecting the Optimally Describing Isoline (ODI)**

Now the raster map is to be transformed to a vector map of consecutive contour lines, encircling areas where the site density does not fall under certain threshold values, i.e. density isolines (Step 9). Calculating these contour lines from the Kriging map usually requires a step parameter (equidistance) to be entered, i.e. the distance between each line. The chosen equidistance directly influences the resolution of the resulting ODI and depends very much on the scale of the study area. Therefore, we consider it reasonable to estimate the value. Here, a pre-computed Ripley's K may be consulted again to estimate a reasonable value (see above). If a pronounced spatial clustering is present in the data set, it is indicated by a peak in the upper part of the graphical output; the scale at which spatial clustering is most pronounced usually can be determined from the x-axis.

While Ripley's K indicates the scale of spatial clustering of sites, it does not allow delineation of particular clusters. To obtain information on increases in area and site number between consecutive isolines, the information is extracted (Step 10) and – except for "R" – exported for further exploration into a spreadsheet program (Step 11). The question to be answered there is: which of these isolines describes Core Areas of intense settlement activity the best (which is the ODI – Step 12). Three measures help to identify the ODI.

The first (1) criterion is the maximum increase of space, which takes advantage of an intrinsic property of the value distribution. Those areas showing the highest site densities are encircled by Isolines following the smallest LEC values. Moving "outward" from these centres of density along the consecutive isolines of ever-increasing LEC values (decreasing density), the area encircled by each consecutive isoline grows rapidly in all directions away from these centres – up to a certain point. At larger LEC values, the contour polygons have grown so large that they cover complete site clusters and start approaching neighbouring clusters. At this point, the areal growth of consecutive isolines starts declining. This boundary effect between densely settled areas is taken as highly indicative of the ODI. To facilitate a reading of the corresponding values, a graphic visualisation of the standardised increase of the areas encircled by consecutive isolines is generated (for details see manuals). If the graph shows only a single peak value, the identification of the ODI is straightforward. Since the graph's structure is a function of a density proxy generalised for the entire area of investigation (up to a continental scale), it is influenced by varying cluster structures. The effect of these differences is particularly pronounced when clusters of varying density exhibit different transitions from high to low density at their borders, i.e. when the borders of some clusters are sharply pronounced while those of others are more blurred. This makes clear that the ODI selection based on areal increase alone is connected to a measurement imprecision which has to be dealt with. Therefore, two additional heuristic criteria have to be applied to ensure a reasonable choice of the ODI (Zimmermann et al. 2009a,b). These criteria state that the ODI should (2) encircle the included sites as closely as possible without resulting in highly fragmented isoline patches, (3) enclose at least 75% of all sites in the area of observation.

Generally, ODIs need to be cross-checked for edge effects, especially when calculated for large areas. The likeliness of a strongly heterogeneous distribution of densities is higher at larger spatial scales. Our case studies show that LECs exceeding 40 km require particularly careful evaluation.

Regions enclosed by the ODI are called Core Areas, the sizes of which are equal to the areas encircled by the selected ODIs.

# 2. Estimating population sizes and densities

## 2.1. Calculation and underlying theoretical assumptions for sedentary societies

For estimates of population sizes in sedentary societies, the Cologne Protocol combines the areal ODI-data with areal data of social units derived from archaeological research in so-called 'Key Areas'. These Key Areas are regions of between 10 and a few hundred km² that stand out because of their exceptional archaeological survey coverage which can be considered complete or almost complete. This means that all or at least most of the sites in these areas are likely to be known. For such Key Areas, the space available per social units (i.e. household or person) can be estimated. These social units can thus be connected to areal data specific to and characteristic of a certain time and area. By putting the areal data of the social units in relation to the Core Areas, the archaeologically derived information on numbers and densities of people can be upscaled to the Core Area level.

In the following, the fundamental assumptions leading to the population densities presented here are summarised; for an in depth discussion please refer to Wendt et al. 2010; Zimmermann et al. 2004; Zimmermann et al. 2009a & 2009b.

Examples of Key Areas are the settlements of the Bandkeramik (Linearbandkeramik, or LBK, culture) of the Aldenhovener Platte, a small region of approximately 150 km$^2$ located in the lignite mining area between Cologne and Aachen. Here, all Bandkeramik sites have either been excavated completely or the number of contemporaneous houses can be reliably estimated. For the middle of the 51$^{st}$ century, when populations were at their maximum in this area, a density of about one household per square kilometre is observed. An average number of 8.5 inhabitants is assumed for each Bandkeramik longhouse, a value confirmed in a series of combined settlement and cemetery excavations. Judging from analyses of ceramic production, settlement and $^{14}$C dates, an average life expectancy of 25 years per house seems appropriate (Stehli 1989).

Estimations of later periods have to be adjusted according to the rising level of complexity in land use. For the Iron Age, three independent estimates had to be carried out and combined; the fertile loess areas of the so-called '*Altsiedellandschaften*' ('old settlement land'), the two upland regions of Hunsrück and Eifel, and the lowlands of the northern Rhineland. For the Loess region, settlement data for social units could be inferred similarly to those described for the LBK, while for the other regions information from necropolises of tumuli with differing states of preservation had to be used as the basis for the calculations.

For the Roman age, the area under consideration had to be subdivided into high and low density regions. Through the outstanding archaeological visibility of Roman stone structures at the level of Key Areas as well as in distribution maps of larger regions, an upscaling procedure for the agrarian Roman landscape was unnecessary. The final population density was a combination of the data from within the ODIs regarding the numbers of individuals per villa and numbers of town inhabitants (municipia, vici) as well as military personnel.

The estimation of the Merovingian period with an excellent archaeological chronology is based on the analysis of necropolises. The density calculations for the time period of AD 1800 are based on a statistical description of the Rhine province (von Restorff 1830). The data for these two time periods are judged as reliable.

## 2.2. Calculation and underlying theoretical assumptions for foragers

For the estimation of population sizes of forager societies, the Cologne Protocol uses the size of Core Areas, the size and variability (1st and 3rd quartile) of raw material catchment areas (raw material polygons), and ethnographically documented group sizes (GROUP2 units according to Binford 2001). We use ethnographically documented group size (Binford 2001) instead of population density, since this value has been less prone to biases than any other ethnohistorically reported information on demography (e.g. population density). Basic assumptions, selection criteria, and calculation formulas are outlined in the following sections.

### 2.2.1. From raw material acquisition to the number of groups per Core Areas

Having identified the ODI and therefore described the Core Areas, a second spatial entity is needed to determine how many GROUP2 units can be considered for each Core Area. Here, Raw Material Catchments play a key role as they mirror a specific land-use of hunter-gatherers within each individual Core Area.

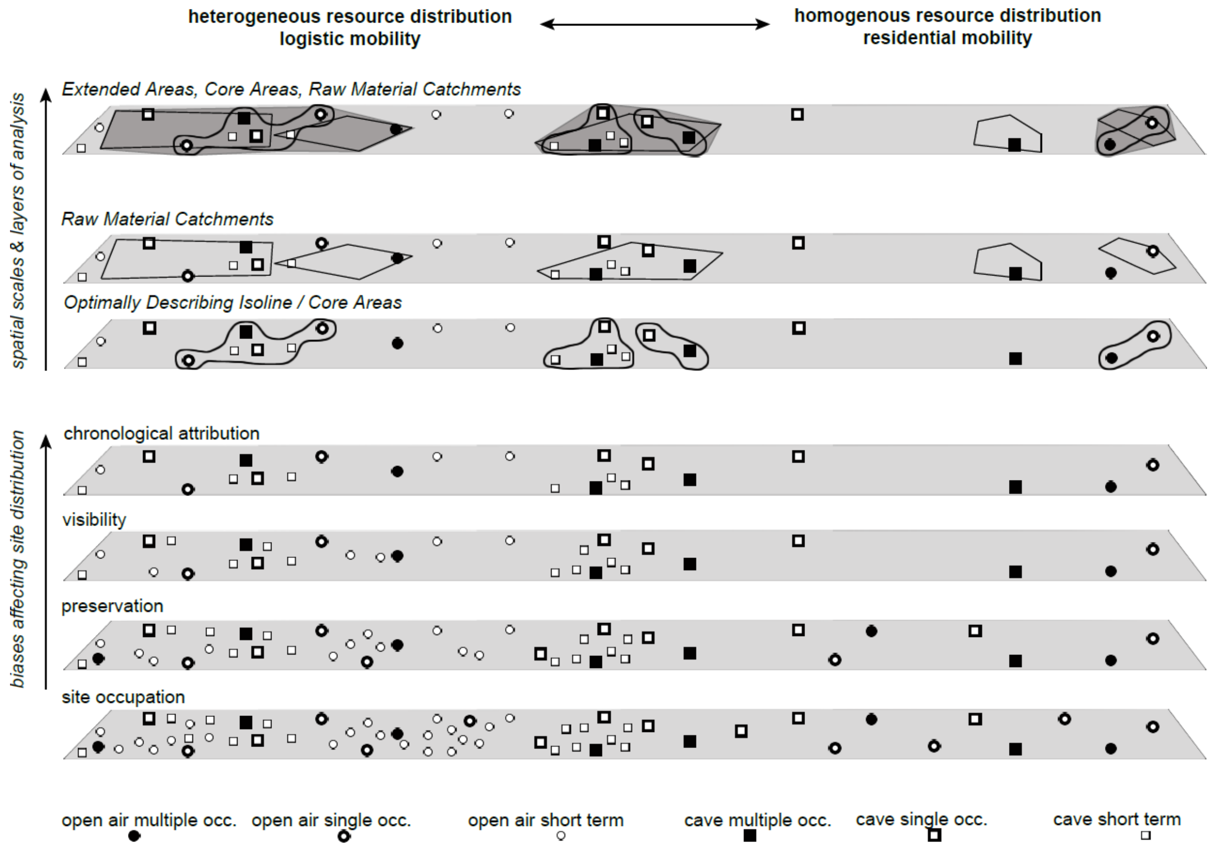


**Fig. S2** Schematic illustration on possible effects of forager's mobility patterns on site distributions, and biasing factors and the modeling outcome of the Cologne Protocol. Residential mobility might result in a generally lower density of sites (modified after: Kretschmer et al. 2016: Fig. 1).

Three basic assumptions are important. The first – following Dyson-Hudson & Smith (1978) and Kelly (1995: 161pp.) – is that in environments where resource locations are predictable and clustered (**Fig. S2**), hunter-gatherers tend to form non-overlapping territories (though they are not necessarily

defended). Throughout the Weichselian Glacial, we assume an aggregated and predictable resource distribution, as this glacial is usually connected with cold to cold-temperate environments with gregarious animals and a marked seasonality (Binford 2001).

The second basic assumption is that raw material acquisition took place as "embedded procurement" (Binford 1979: 259), which envisions the gathering of lithic raw materials as embedded in other tasks of the daily routine. A raw material catchment area, reconstructed from the lithic assemblage at an archaeological site, is thus considered as reflecting the movement of the relevant group of people in the landscape. The catchment area would therefore outline a minimum area of land tenure and resource exploitation.

The third assumption is that each catchment area is inhabited by a group of the size of a GROUP2 social unit, defined as the mean group size of the largest residential seasonal camp (Binford 2001). This is because the raw material spectrum is expected to reflect the entire, annual round of the group, not just a seasonal section of the group's mobility (Kretschmer in: Widlock et al. 2012; Kretschmer 2019: 234-235). This assumption also justifies specific criteria to exclude catchments from further analysis (see below).

It follows from these assumptions that raw material catchments are considered indicative of the spatial extent of a single socio-economic unit, comprising a GROUP 2 unit. Core Areas can be made up of several GROUP 2 units. Raw material catchments can also either extend beyond Core Areas or link otherwise disconnected Core Areas. In those cases, these additional areas and connected Core Areas are combined in a spatial super-structure called an Extended Area (see **Figure S2**) by creating a convex hull.

It is important to stress that the logical relationship between the Core Areas, the raw material catchments and the number of GROUP2 units per Core Area (or Extended Area) is neither that of a simple areal size function, nor that of an abstract carrying capacity. The point of concern is that the spatial relationship between the observable raw material catchments is thought to reflect the socio-economic structure of the population in a given Core Area. Thus, by combining the observations on Core Areas and raw material catchments, inferences about the population structure can be made. Within the subsequent calculation, larger catchments will result in lower population estimates per Core Area, whereas small catchments will result in higher population sizes (**Table S4**). This is because diverse and resource-rich environments can potentially support more people within the same space than areas with a poorer resource structure.

In order to obtain the number of GROUP2 units within Core Areas and Extended Areas, the available information on raw material sources and transport distances from all sites is collected. For each assemblage, the source areas of lithic raw materials are mapped and a convex hull is created around the site (which is buffered by a 5 km radius) and its related sources. The convex hull is the so-called 'raw material polygon' for which we then calculate the areal size. It is important to keep in mind that we are aiming at a regional spatial scale, where raw material acquisition is informative for territorial structures. Therefore, we a) summarise data on local raw materials occurring within a 5 km radius (buffer) around the site (and thus within the area of daily exploitation), and b) exclude material apparently transported over long distances that violate the assumption of embedded procurement (Féblot-Augustin 1993, 2009). Violation is defined here if raw materials occur singly or in very low numbers or percentages, or if transport distances are significantly above the general trend and therefore the lithics might have reached the site rather by long-distance trips of individuals or "down-the-line" exchange, as assumed for mollusc shells (Maier 2015). Also excluded from the sample are small raw material polygons, located within a daily exploitation radius around the site, or assemblages for which only one single non-local raw material source can be detected, since they are likely not related to annual or seasonal land-use patterns and were repeatedly identified as clear areal outliers

in our samples. For Magdalenian periods histograms were used to identify raw material polygon sizes of annual catchment areas (Kretschmer 2015, 2019).

**Table S4:** Schematic illustration of the relation between the size of Core Areas (grey areas) and raw material catchments (blue polygons). If catchments are small, estimates on population size will be generally higher, while larger catchments will produce lower estimates. Potential adjustments of the ethnographic group size, so far not applied but discussed in the main text of the article, are given in parentheses.

| | **Small Core Areas** | **Large Core Areas** |
|---|---|---|
| **Small RM Polygons** | Few groups 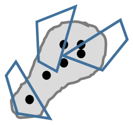 | Many groups (Group 1?) 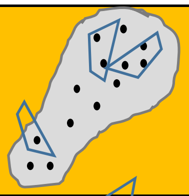 |
| **Large RM Polygons** | Very few groups (Group 3?) 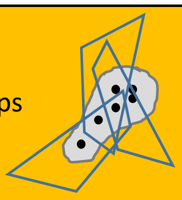 | Few groups 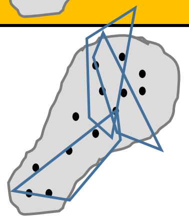 |

With reference to the third assumption mentioned above, we divide the extent of the Core Area by the mean area (2nd quartile) of all raw material polygons of the specific Core Area (or Extended Area) in order to obtain the mean number of GROUP2 units per Core Area. Many small raw material polygons will thus result in a high number of GROUP2 units whereas large polygons will result in a low number (see **Table S4**). To account for the present range in the polygon spectrum, we also calculate the maximum and minimum number of groups using the 1st and 3rd quartile of the raw material polygons.

$$\text{n groups}_{\,(\text{min., mean, max.})} = \frac{km^2 within\ Core\ Areas}{Quartile\ (Q1;Q2;Q3)\ of\ raw\ material\ polygons} \quad (1)$$

It needs to be stressed that this approach relies on well-studied raw material economies. In areas where information on raw material catchments is lacking (see also discussion in Section "Future avenues and challenges in modelling population estimates"), we transfer the catchment sizes from neighbouring areas. In order to keep the basic assumptions of the protocol warranted, it is important to select only those areas for data transfer that exhibit comparable environmental conditions.

### 2.2.2. From numbers of groups to absolute estimates of densities and people

Having estimated the number of GROUP2-units per Core Area and Extended Area, it is necessary to make inferences about the actual number of persons per GROUP2-unit in order to arrive at estimates of absolute numbers of people in the investigated area. In the archaeological literature, the "magic number" of 25 persons per group has been quoted ever since its introduction by J. H. Steward (1936) as an optimal size for a hunter-gatherer groups and has been used in many studies and calculations (e.g. Wobst 1974). As a matter of fact, many citations of group sizes in archaeology have ranged

between 20 and 30 persons (e.g. Hassan 1981: 60-61), while other studies identified aggregated group sizes of 30-50 persons (Hamilton et al. 2007, Dunbar 2012, 2014. The size of GROUP2-units varies in relation to different criteria like environmental conditions, subsistence strategies or degree of mobility. The selection criterion of having a subsistence strategy similar to those strategies employed by Palaeolithic foragers turned out to be the best one (Kretschmer, 2015: 43-56). The database of Binford (2001) comprises data on the proportions of hunting, fishing and gathering activities among ethnographically documented hunter-gatherer societies. The setting of framework conditions for the selection criterion has to be primarily based on archaeological data.

The idea of dominant hunting subsistence strategies during the Palaeolithic is supported by studies of Pleistocene hunter-gatherer diet and analyses of excavated materials showing a strong reliance on meat, especially of terrestrial ungulates (e.g. Hahn 1983: 317-321; Delpech, 1983; Gordon 1988; Charles 1998; Bignon 2008).

However, in recent studies, the importance of aquatic resources has been pointed out. A direct proof of fish in the faunal remains is often problematic, since the small and fragile bones are easily overlooked or simply often badly (or not at all) preserved. Nevertheless, there exist several examples of Late Upper Palaeolithic sites with fish remains (e.g. Cleyet-Merle 1990; Le Gall 1992; Van Neer et al. 2007; Torke 1981). The increase of barbed-points (harpoons) and depictions of aquatic animals (e.g. Bosinski 2008; Cleyet-Merle 1990; Julien 1982) could be seen as indications of the significance of aquatic resources. The consumption of fish is also indicated by stable isotope analyses of bone collagen (e.g. Hayden et al. 1987; Richard et al. 2005; Richard & Hedges 1999).

**Table S5:** Group size and economic basis of 16 hunter-gatherer groups (data taken from Binford 2001) selected as samples for the cited Upper Palaeolithic studies (see also: Kretschmer 2015: 53, Table 6-3).

| Name of h&g group | Region | % Gathering | % Hunting | % Fishing | Group 1 (n) | Group 2 (n) | Group 3 (n) |
|---|---|---|---|---|---|---|---|
| **Round Lake Ojibwa** | Ontario | 15 | 65 | 20 | 7 | 50 | 150 |
| **North Saulteaux** | Ontario | 5 | 65 | 30 | 15 | 45 | 185 |
| **Rainy River Ojibwa** | Ontario | 10 | 60 | 30 | 21 | 57 | 190 |
| **Nunamiut Inuit** | Alaska | 0,1 | 89 | 10,9 | 18,5 | 25,1 | 75 |
| **Noatak Inuit** | Alaska | 0,1 | 75 | 24,9 | 17,5 | 30 | 105 |
| **Ona** | Isla Grande de Tierra del Fuego | 5 | 75 | 20 | 20 | 45 | 290 |
| **Kaska** | British Columbia | 10 | 65 | 25 | 16 | 58 | 139 |
| **Tahltan** | British Columbia | 10 | 60 | 30 | | 71 | 165 |
| **Mistassini Cree** | Labrador Peninsula (Quebec) | 0,9 | 74 | 25,1 | 6 | 37 | 215 |
| **Sekani** | Alberta | 10 | 60 | 30 | 18 | 40 | 164 |
| **Naskapi** | Quebec | 1 | 73 | 26 | 23 | 39 | 117 |
| **Mountain** | Northwest Territories | 10 | 75 | 15 | 15 | 60 | |
| **Satudene** | Northwest Territories | 3 | 75 | 20 | 12,5 | 29 | |
| **Hare** | Northwest Territories | 10 | 65 | 25 | 13 | 26 | 120 |
| **Slave** | Northwest Territories | 10 | 60 | 30 | 13 | 39 | 220 |
| **Chippewyan** | Saskatchewan | 5 | 70 | 25 | 23 | 75 | 295 |
| **Q1** | | | | | 13 | 35 | 125 |
| **Median** | | | | | 16 | 43 | 165 |
| **Q3** | | | | | 19 | 57 | 209 |

Demonstrating the use of vegetal resources during the Upper Palaeolithic is more difficult; however, several lines of direct and indirect evidence reveal the importance and changing role of plants in Upper Palaeolithic diet (Power & Williams 2018). Arguments come also from the availability of a wide range of edible plants in this time period as well as nutritional needs of human population and ethnographic observations (e.g. Owen 1996, 2002; Speth 1991).

In general, terrestrial animals have been proven to be important in the nutrition of Palaeolithic foragers. The expectation of more than 50 % hunting in subsistence strategies is therefore set as a selection criterion. In contrast, the maximum contribution of aquatic resources has never been reported as more than 30 % proportion of dietary intake, thus the subsistence strategies of fishing or gathering should be below this value (Kretschmer 2015: 52-53).

To arrive at reliable estimates of the size of GROUP2- units, we only selected from the ethnographic record of 339 documented hunter-gatherer cases (Binford 2001) those that met these requirements, while excluding mounted groups. This left us with 16 hunter-gatherer groups (**Table S5** after Kretschmer 2015), whose consumption ratios varied between 60 and 89 % for hunting, 11 and 30 % for fishing, and 0.1 and 15 % for gathering (Kretschmer 2019). The number of persons in GROUP2 units for these cases ranges between 35 (1st Quartile) and 57 (3rd Quartile) persons with a median number of 43 persons. Archaeological findings of excavated habitation structures corroborate these ethnographic observations as there is no evidence for larger groups in the Palaeolithic record. Information on the size of Upper Palaeolithic dwellings like in Gönnersdorf and Andernach-Martinsberg in the Middle Rhine region (Bosinski 1979; Holzkämper 2006; Sensburg 2007; Terberger 1997) or Pincevent and Etiolles in the Paris Basin (Audouze 1992; Julien 2006; Leroi-Gourhan 1984) show that group sizes from 35 to 57 persons should be possible, especially taking into account that the full areas of these sites are not completely excavated. As a result, the average size of 43 persons per Catchment Area has been used in all calculations of population densities so far (e.g. Kretschmer 2015: 55-56; Maier & Zimmermann 2017; Maier et al. 2016; Schmidt & Zimmermann 2019).

By multiplying the number of GROUP2-units per Core Area or Extended Area with the estimated median number of persons per GROUP2-unit (43), we calculate the absolute number of persons per region. Eventually, we calculate the population density within Core areas (regional population density) and Extended areas (supra-regional population density) as well as within a larger map section or the whole investigated area (Total Area of Calculation).

$$\text{Core Area population size} = \text{n groups} \times \text{group size} \quad (2)$$

$$\text{population density within Core Area} = \frac{Core\ Area\ population\ size}{km^2 within\ Core\ Area} \quad (3)$$

$$\text{population density within Extended Area} = \frac{Core\ Area\ population\ size}{km^2 within\ Extended\ Area} \quad (4)$$

$$\text{population density within Total Area of Calculation} = \frac{Core\ Area\ population\ size}{km^2 within\ Total\ Area\ of\ Calculation} \quad (5)$$

**No need to calibrate by length of each period**

In contrast to other approaches, no specific calibration procedure is required when conducting diachronic comparisons of the results obtained with the Cologne Protocol. Each site-distribution of a defined period produces Core Areas which can be directly compared to Core Areas of another period. Methodologically, the maximum temporal resolution in the Cologne Protocol is only delimited by the chrono-cultural resolution of the archaeological record. Limits for a minimum temporal resolution need to be evaluated against blurring effects, e.g. internal changes in subsistence or mobility strategies. For example, if distribution patterns of Core Areas are mutually exclusive between two succeeding periods, the Core Area for the entire dataset will be larger than the Core Area of each of the two periods. This in turn would result in an overestimate of the population size and density (Schmidt & Zimmermann in press). This effects is shown for a case study on the Late Palaeolithic of Europe, for which a subdivision (affected by regional taxonomic uncertainties and a general scarcity of direct dating, e.g. Sauer & Riede 2018) produced much lower results than a combined estimate for the entire period. The resulting difference is displayed in Fig. 3 (see Schmidt & Zimmermann in press). Conversely, we can argue that blurring effects will remain low if spatially exclusive settlement patterns can be considered unlikely. Thus, although calibration is not methodologically necessary, a critical evaluation of biases within the archaeological record has to be conducted prior to – and during – interpretation.

**Table S6:** Parameterisation of Fig. 1. Please see Main Text Section "Population size and density estimates – results for foraging and farming societies" for further details.

| | Time intervals (years) | Total Area of Calculation (km²) | Growth factor per generation (25 years) | Initial value | K1 | K2 dependent on estimations of population density |
|---|---|---|---|---|---|---|
| States with high energy consumption | 50 | 360,000 | 1.25 | - | 0.903 | 0.05 – 0.2 |
| States with low energy consumption | 50 | 360,000 | 1.2 – 1.24 | 0.1 | 0.553 | 0.01 – 0.3 |
| Farmers | 100 | 20-40,000 | 1.18 | 0.05 | 0.303 | 0 – 0.03 |
| Hunter-Gatherers | 500 | 2.3 Mio | 1.15 | 0.005 | 0.03 | 0 – 0.4 due to inflation |

## 3. References

Ahlrichs JJ, Henkner J, Schmidt K. Seamless workflow for defining archaeological site densities with contour lines by using the open source (geo-)statistical language R. Technical note 1: Collaborative Research Center 1070 – Geoscientific and Archaeological Research (Tübingen 2016). https://uni-tuebingen.de/forschung/forschungsschwerpunkte/sonderforschungsbereiche/sfb-1070/organisation/serviceprojekt-s/technical-notes/

Audouze F. L'occupation magdalénienne du Bassin Parisien. In: JPh. Rigaud/H. Laville/B. Vandermeersch (eds.), Le Peuplement Magdalénien. Paléogéographie physique et humaine. Colloque de Chancelade 10–15 octobre 1988 (Paris 1992) 345–356.

Baddeley A, Rubak E, Turner R. 2015: Spatial Point Patterns: Methodology and Applications with R Chapman and Hall/CRC Press: Boca Raton.

Bevan, A., Conolly, J., 2006. Multiscalar Approaches to Settlement Pattern Analysis, in: Lock, G., Molyneaux, B.L. (Eds.), Confronting Scale in Archaeology: Issues of Theory and Practice. Springer US, Boston, MA, pp. 217–234. https://doi.org/10.1007/0-387-32773-8_15

Bignon O. 2008: Chasser les chevaux a la fin du Paleolitique dans le Bassin parisien: Strategie cynegetique et mode de vie au Magdalenien et a l'Azilien ancien, Bar S Series 1747. British Archaeological Reports : Oxford.

Binford LR. 2001: Constructing Frames of Reference. An Analytical Method for Archaeological Theory Building Using Hunter-Gatherer and Environmental Data Sets. University of California Press: Berkeley.

Binford LR. Organization and formation processes: Looking at curated technologies. Journal of Anthropological Research. 1979; 35(3): 255-273.

Bosinski G. 1979: Die Ausgrabungen in Gönnersdorf 1968–1976 und die Siedlungsbefunde der Grabung 1968. Der Magdalénien-Fundplatz Gönnersdorf 3. Steiner: Stuttgart.

Bosinski G.2008: Urgeschichte am Rhein. Tübinger Monographien zur Urgeschichte. Kerns: Tübingen.

Bowman A, Azzalini A. 1997: Applied smoothing techniques for data analysis : the kernel approach with S-Plus illustrations. Oxford statistical science series 18. Clarendon Press: Oxford.

Charles R. 1998: Late Magdalenian chronology and faunal exploitation in the north-western Ardennes, BAR International Series 737. Archaeopress: Oxford.

Claßen E. Siedlungen der Bandkeramik bei Königshoven. Mit einem Beitrag von Ursula Tegtmeier. Rheinische Ausgrabungen Band 64. Verlag Philipp von Zabern: Darmstadt. 2012. Zugl. Universität zu Köln Dissertation 2007.

Cleyet-Merle JJ. 1990: La préhistoire de la pêche. Errance: Paris.

Conolly, J., Lake, M., 2006. Geographical information systems in archaeology, Cambridge Manuals in Archaeology. Cambridge University Press, Cambridge. https://doi.org/10.1017/CBO9780511807459

Cüppers H, Rüger ChB. 1985: Römische Siedlungen und Kulturlandschaften. Geschichtlicher Atlas der Rheinlande Beih. III/1-2. Rheinland Verlag: Köln.

Delpech F. 1983: Les faunes du Paléolithique supérieur dans le Sud-Ouest de la France. Éditions du Centre national de la recherche scientifique: Paris.

Dunbar RIM. Bridging the bonding gap: the transition from primates to humans. Philosophical Transactions of the Royal Society B: Biological Sciences [Internet]. 2012 Jul 5;367(1597):1837–46. Available from: http://dx.doi.org/10.1098/rstb.2011.0217

Dunbar RIM. The Social Brain. Current Directions in Psychological Science [Internet]. 2014 Apr;23(2):109–14. Available from: http://dx.doi.org/10.1177/0963721413517118

Duong T, Cowling A, Koch I, Wand MP. Feature significance for multivariate kernel density estimation. Computational Statistics & Data Analysis [Internet]. 2008 May;52(9):4225–42. Available from: http://dx.doi.org/10.1016/j.csda.2008.02.035

Duong T, Wand M. feature: Local Inferential Feature Significance for Multivariate Kernel Density Estimation. R package version 1.2.13. 2015; (2015-10-26). Available from: https://CRAN.R-project.org/package=feature

Duong T. ks: Kernel Smoothing. R package version 1.10.2. 2016; [2016-02-29]. Available from: https://CRAN.R-project.org/package=ks

Ebrahim GJ. Statistical analysis and modelling of spatial point patterns * Illian J., Penttinen A., Stoyan H., Stoyan D. (eds). Journal of Tropical Pediatrics [Internet]. 2007 Nov 29;55(1):69–69. Available from: http://dx.doi.org/10.1093/tropej/fmn022

Féblot-Augustins J. Mobility Strategies in the Late Middle Palaeolithic of Central Europe and Western Europe: Elements of Stability and Variability. Journal of Anthropological Archaeology [Internet]. 1993 Sep;12(3):211–65. Available from: http://dx.doi.org/10.1006/jaar.1993.1007

Féblot-Augustins J. Revisiting European Upper Paleolithic Raw Material Transfers: The Demise of the Cultural Ecological Paradigm? In: Lithic Materials and Paleolithic Societies [Internet]. Wiley-Blackwell; 2009. p. 25–46. Available from: http://dx.doi.org/10.1002/9781444311976.ch3

Fortin M-J, Dale MRT. Spatial Analysis [Internet]. Cambridge University Press; 2005. Available from: http://dx.doi.org/10.1017/CBO9780511542039

Gordon BC. 1988: Of men and reindeer herds in French Magdalenian prehistory. British Archaeological Reports, BAR International Series 390. B.A.R.: Oxford.

Hahn J. 1983 Eiszeitliche Jager zwischen 35 000 und 15 000 vor heute. In: Müller-Beck H. (eds.), Urgeschichte in Baden-Württemberg (Stuttgart 1983) 273–330.

Hamilton MJ, Milne BT, Walker RS, Burger O, Brown JH. The complex structure of hunter–gatherer social networks. Proceedings of the Royal Society B: Biological Sciences [Internet]. 2007 Jul 3;274(1622):2195–203. Available from: http://dx.doi.org/10.1098/rspb.2007.0564

Hassan FA. Demographic Archaeology. In: Advances in Archaeological Method and Theory [Internet]. Elsevier; 1981. p. 225–79. Available from: http://dx.doi.org/10.1016/B978-0-12-624180-8.50010-X

Hayden B, Chisholm B, Schwarcz HP. Fishing and Foraging. Marine Resources in the Upper Paleolithic of France. In: Soffer O. (ed.), The Pleistocene Old World: Regional Perspectives (London 1987) 279–291.

Herzog I. Aus Punkten werden Flächen. Vorschlag einer Methodik zur Abgrenzung von Gebieten mit hoher Fundpunktdichte. Berichte der Römisch-Germanischen Kommission 91 (Frankfurt a. M. 2010) 37-95.

Heuvelink GBM. R. Webster, M.A. Oliver: Geostatistics for Environmental Scientists. Mathematical Geosciences [Internet]. 2009 Mar 20;41(4):487–9. Available from: http://dx.doi.org/10.1007/s11004-009-9221-9

Holzkämper J. 2006: Die Konzentration IV des Magdalénien von Andernach-Martinsberg, Grabung 1994–1996 (Universität Köln 2006).

Joachim HE. 1997: Bronze- und Eisenzeit. Geschichtlicher Atlas der Rheinlande, Beiheft II/3.1-II/3.4. Rheinland-Verlag: Köln.

Julien M. (1982). Les harpons magdaleniens. 17e supplement a Gallia prehistoire, C.N.R.S. Paris.

Julien M. 2006: À la recherche des campements d'hiver dans le Magdalénien du Bassin parisien. Bulletin de la Société Préhistorique Française. 2006; 103(4): 695-709.

Kretschmer I. 2015: Demographische Untersuchungen zu Bevölkerungsdichten, Mobilität und Landnutzungsmustern im späten Jungpaläolithikum. Kölner Studien zur Prähistorischen Archäologie 6. Verlag Marie Leidorf: Rhaden.

Kretschmer I. Demographic studies of hunters and gatherers in the European Late Upper Palaeolithic. In: Eriksen BV, Rensink E, Harris S. (eds.), The Final Palaeolithic of Northern Eurasia. Proceedings of the Amersfoort, Schleswig and Burgos UISPP Commission Meetings (Kiel 2019) 231-245.

Inga Kretschmer, Andreas Maier und Isabell Schmidt. (2016) Probleme und mögliche Lösungen bei der Schätzung von Bevölkerungsdichten im Paläolithikum. In: T. Kerig, K. Nowak, G. Roth (Hrsg), Alles was zählt... Festschrift für Andreas Zimmermann. UPA 285 (Bonn 2016).

Le Gall O. Les Magdaléniens et l'Ichtyofaune Dulcaquicole. In: Rigaud JPh, Laville H, Vandermeersch B. (eds.), Le Peuplement Magdalénien. Paléogéographie physique et humaine. Colloque de Chancelade 10–15 octobre 1988 (Paris 1992) 277–285.

Leroi-Gourhan A. 1984: Pincevent. Campement magdalénien de chasseurs de rennes. Guides archéologiques de la France 3. Ministère de la Culture, Direction du Patrimoine, Sous-direction de l'Archéologie : Imprimerie nationale: Paris.

Maier A, Lehmkuhl F, Ludwig P, Melles M, Schmidt I, Shao Y, et al. Demographic estimates of hunter–gatherers during the Last Glacial Maximum in Europe against the background of palaeoenvironmental data. Quaternary International [Internet]. 2016 Dec;425:49–61. http://dx.doi.org/10.1016/j.quaint.2016.04.009

Maier A, Zimmermann A. CRC806-E1 Gravettian-Sites Database V-20160219. CRC806-Database [Internet]. 2016; http://dx.doi.org/10.5880/SFB806.18

Maier A, Zimmermann A. CRC806-E1 LGM-Sites Database V-20150313. CRC806-Database [Internet]. 2015. http://dx.doi.org/10.5880/SFB806.12

Maier A, Zimmermann A. Populations headed south? The Gravettian from a palaeodemographic point of view. Antiquity [Internet]. 2017 Jun;91(357):573–88. Available from: http://dx.doi.org/10.15184/aqy.2017.37

Maier A. The Central European Magdalenian [Internet]. Vertebrate Paleobiology and Paleoanthropology. Springer Netherlands; 2015. Available from: http://dx.doi.org/10.1007/978-94-017-7206-8

Nieveler E. 2006: Merowingerzeitliche Besiedlung. Archäologische Befunde in den nördlichen Rheinlanden. Geschichtlicher Atlas der Rheinlande Beih. 4, 10. Habelt Verlag: Bonn.

O'Sullivan D, Unwin D. Geographic Information Analysis Wiley, Hoboken, 2002

Owen LR. Der Gebrauch von Pflanzen im Jungpaläolithikum Mitteleuropas. Ethnographisch-Archäologische Zeitschrift. 1996; 37: 119–146.

Owen LR. Reed tents and straw baskets? Plant resources during the Magdalenian of Southwest Germany. In: SLR. Mason/JG. Hather (eds.), Hunter-gatherer archaeobotany. Perspectives from the northern temperate zone (London 2002) 156–173.

Power RC, Williams FL. Evidence of increasing intensityof food processing during the Upper Palaeolithic of western Eura-sia. Journal of Palaeolithic Archaeology. 2018; 1: 281–301.

Preparata FP, Shamos IS. 1988: Computational Geometry: an Introduction. Springer: New York.

Preuss, J., ed. 1998. Das Neolithikum in Mitteleuropa: Kulturen, Wirtschaft, Umwelt vom 6. bis 3. Jahrtausend v.u.Z., Übersichten zum Stand der Forschung. Weissbach, Germany: Beier & Beran.

Restorff FV. 1830: Topografisch-Statistische Beschreibung der Königlich Preußischen Rheinprovinzen. Nicolai: Berlin und Stettin.

Richards MP, Hedges REM. Stable Isotope Evidence for Similarities in the Types of Marine Foods used by Late Mesolithic Humans at Sites along the Atlantic Coast of Europe. Journal of Archaeological Science. 1999; 26: 717–722.

Richards MPR, Jacobi R, Cook J, Pettit PB, Stringer CB. Isotope evidence for the intensive use of marine foods by Late Upper Palaeolithic humans. Journal Human Evolution. 2005; 45: 390-394.

Richter J. 1997: Neolithikum / von J. Richter unter Mitarbeit von E. Claßen, mit einem Beitrag von A. J. Kalis und J. Meurers-Balke. Geschichtlicher Atlas der Rheinlande Beiheft II/2.1-II/2.2. Rheinland-Verlag: Köln.

Ripley, B.D. 1976. The second-order analysis of stationary point processes. Journal of Applied Probability 13, 255–266. https://doi.org/10.2307/3212829

Schmidt I. CRC806-E1 Raw-material-polygons. Database on the Late Palaeolithic of Europe V2019-05-08. CRC806-Database, 2019. University of Cologne, https://doi.org/0.5880/SFB806.48

Schmidt I, Zimmermann A. CRC806_E1_EUP_Sites_Database_20170721 [Internet]. CRC806-Database; 2018. University of Cologne, https://doi.org/10.5880/SFB806.42

Schmidt I, Zimmermann A. CRC806-E1 Database on Final Palaeolithic Sites of Europe V2020-08-20. CRC806-Database, 2020. University of Cologne, https://doi.org/10.5880/SFB806.51

Schmidt I, Zimmermann A. Population estimates for the Final Palaeolithic (14,000 to 11,700 years cal. BP) of Europe – challenging evidence and methodological limitations. Bulletin de la Societe Prehistorique Francaise, (in press).

Sensburg M. 2007: Die räumliche Organisation der Konzentration IIa von Gönnersdorf. Struktur und Dynamik eines magdalénienzeitlichen Siedlungsbefundes am Mittelrhein, Monogr. RGZM 69. Römisch-German. Zentralmuseum: Mainz.

Speth JD. Nutritional constraints and Late Glacial adaptive transformations: The importance of non-protein energy sources. In: Barton N, Roberts AJ, Roe DA(eds.), The Late Glacial in north-west Europe:

Human adaption and environmental change at the end of the Pleistocene. CBA Research Report 77 (Oxford 1991) 169–178.

Stehli P. Zur relativen und absoluten Chronologie der Bandkeramik in Mitteleuropa. In: Rulf J. (eds.), Bylany Seminar 1987. Collected Papers. (Prague 1989) 69-78.

Steward JH. The economic and social basis of primitive bands. In: Kroeber AL. (Ed.), Essays in Anthropology. (Berkeley 1936) 331-350.

Terberger Th. 1997: Die Siedlungsbefunde des Magdalénien-Fundplatzes Gönnersdorf. Konzentrationen III und IV, Der Magdalénien-Fundplatz Gönnersdorf Bd. 6. Franz Steiner Verlag: Stuttgart.

Torke W. 1981: Fischreste als Quellen der Ökologie und Ökonomie in der Steinzeit Südwest-Deutschlands. Urgeschichtliche Materialhefte 4. Distribution by Moreland International: Bad Bramstedt.

Toussaint, G.T., 1983. Computing largest empty circles with location constraints. International Journal of Parallel Programming 12, 347-358. https://doi.org/10.1007/BF01008046

Van Neer W, Wouters W, Germonpré M. Fish remains from three Upper Palaeolithic cave deposits in southern Belgium. Anthropologica et Praehistorica. 2007; 118: 5-22.

Vermeersch PM. Radiocarbon Palaeolithic Europe Database, Version 25. 2019; Available from: http://ees.kuleuven.be/geography/projects/14c-palaeolithic/index.htm

Wendt KP, Hilpert J, Zimmermann A. Landschaftsarchäologie III. Untersuchungen zur Bevölkerungsdichte der vorrömischen Eisenzeit, der Merowingerzeit und der späten vorindustriellen Neuzeit an Mittel- und Niederrhein. Berichte der Römisch-Germanischen Kommission 91 (Frankfurt a. M. 2010) 217-338.

Widlok T, Aufgebauer A, Bradtmöller M, Dikau R, Hoffmann T, Kretschmer I, et al. Towards a theoretical framework for analyzing integrated socio-environmental systems. Quaternary International [Internet]. 2012 Oct;274:259–72. Available from: http://dx.doi.org/10.1016/j.quaint.2012.01.020

Wobst HM. Boundary Conditions for Paleolithic Social Systems: A Simulation Approach. American Antiquity [Internet]. 1974 Apr;39(2Part1):147–78. Available from: http://dx.doi.org/10.2307/279579

Zimmermann A, Richter J, Frank Th, Wendt KP. Landschaftsarchäologie II. Überlegungen zu Prinzipien einer Landschaftsarchäologie. Berichte der Römisch-Germanischen Kommission 85 (Frankfurt a. M. 2004) 37-95.

Zimmermann A, Hilpert J, Wendt KP. Estimations of Population Density for Selected Periods Between the Neolithic and AD 1800. Human Biology [Internet]. 2009a Apr;81(2–3):357–80. Available from: http://dx.doi.org/10.3378/027.081.0313

Zimmermann A, Wendt KP, Frank T, Hilpert J. Landscape Archaeology in Central Europe. Proceedings of the Prehistoric Society [Internet]. 2009b;75:1–53. Available from: http://dx.doi.org/10.1017/S0079497X00000281
