# Supplementary material for: Approaching prehistoric demography: proxies, scales and scope of the Cologne Protocol in European contexts
Source: Philos Trans R Soc Lond B Biol Sci. 2020 Nov 30;376(1816):20190714. doi: 10.1098/rstb.2019.0714 (PMC7741091; doi:10.1098/rstb.2019.0714)
Supplement: Manual S01: Manual and example application to model ‘Core-Areas’ (Optimally Describing Isolines) using MapInfo & Vertical Mapper [file rstb20190714supp2.pdf]

UNIVERSITY OF COLOGNE
Institute for Prehistoric Archaeology

- Abt. Jüngere Steinzeiten
- Archäologisches Zentrum für umwelt- und kulturgeschichtliche Geoinformationen NRW
- SFB 806 Our Way to Europe

Supplementary file for:

Approaching Prehistoric Demography: Proxies, Scales and Scope of the Cologne Protocol in European contexts
Schmidt, Isabell; Hilpert, Johanna; Kretschmer, Inga; Peters, Robin; Broich, Manuel; Schiesberg, Sara; Vogels, Oliver; Wendt, Karl Peter; Zimmermann, Andreas; Maier, Andreas. *Philosophical Transactions of the Royal Society B*

Manual and example application to model 'Core-Areas' (Optimally Describing Isolines) using MapInfo & Vertical Mapper

Karl Peter Wendt
Mai 2020

with the collaboration of
Isabell Schmidt

karl-peter-wendt@t-online.de

This repository contains a manual and example application on how to model the 'Optimally Describing Isoline' (ODI) of the 'Cologne Protocol' using MapInfo GIS Software (see also: https://github.com/C-C-A-A/CologneProtocol-MapInfo/). This modelling approach constitutes the first of two successive tasks within the 'Cologne Protocol' to estimate past population sizes and densities, described in more detail elsewhere (Schmidt et al. 2020: S2.1. and S2.2.).

The manual outlines the technical implementation of working steps 1 to 12 (see Schmidt et al. 2020: Table S2): Firstly a GIS-analysis of site distributions and secondly the identification of the ODI. The working steps include the construction of Voronoi diagrams and "Largest Empty Circles", kriging, converting the kriging results into isolines and finally calculating the criteria to select the ODI.

Rhein LUCIFS
Deutsche Forschungsgemeinschaft DFG

Definition of project:

A calculation of the site density and a selection of the „Optimally Describing Isoline“ will be conducted for a geospatially referenced dataset of archaeological sites.

As a case study we use the distribution map of sites assigned to the early Neolithic period in central Europe (Preuss 1998, map 1; available at CRC 806 database: https://crc806db.uni-koeln.de/start/). Please download the available zip-file in Gauss-Krüger projection (13_earlyNeolithic_CE_sites_GK3.shp), not in WGS84.

The „Optimally Describing Isoline“ – defined based on the density of sites – encloses „Core Areas“, which are geographical areas that were used by prehistoric societies, foragers or farmers; without claiming that the whole designated area was in use at the same time.

The contour of Core Areas should be comprehensible, allowing links to other geographical features such as topography, glaciology, hydrology, soil and precipitation. In the work of the Rhein-LUCIFS project and the CRC 806 „Our Way to Europe“ – Project E1 – defining Core Areas constitutes one working step of the „Cologne Protocol“, which is used to estimate population sizes and densities (see Zimmermann et al. 2009; Schmidt et al. 2020).

All work is done with the programs MapInfo (8.5), Vertical Mapper (3.5) and MS-Excel. Working steps involve geostatistical methods (see p.3). Screenshots on the final pages (20-25) of this manual are made from a German versions of MapInfo and MS-Excel, but translations are provided. We apologise for any inconvenience.

This manual was written for a sample of inland sites from sedentary farming societies in central Europe, dated to roughly 7000 years ago. For datasets of other economies (e.g. foragers) or different environmental settings (e.g. including coastlines or glaciers) alternative working steps are provided in grey letters.

Notation conventions:

Name of file, folder or directory
Colum- or row heading
/Menu / submenu level 1 / submenu level 2 / etc.; button
Option, value entries
(exemplification)
<default value>

Program Versions:
MapInfo 8.5, Vertical Mapper 3.5

Definition of involved working steps:

step	aim	MapInfo/Vertical Mapper:
1	Shape-Layer with sites as points	
2	Creating Voronoi Polygons (VP) around sites	*Vertical Mapper/ Natural Neighbour Analysis/ Create Regions From Points*
3	Extraction of vertices (VP-node points)	*Vertical Mapper/ Create Grid/ Poly-to-Point*
4	Aggregation of vertices	*Vertical Mapper/ Data Aggregation/ Simple Point Aggregation*
5	Defining the radius of the „Largest Empty Circle“	*Distance Calculator*
6	Kriging – Preparations and Grid	*Vertical Mapper/ Create Grid/ Interpolation/ Kriging*
7	Kriging - Semivariogram	*Vertical Mapper/ Create Grid/ Interpolation/ Kriging*
8	Kriging – inspect and export raster output	
9	Creating contour lines (Isolinies)	*Grid Manager/ Contour*
10	Calculating the area and the number of sites per isoline	
11	Data export	*Export to ASCII*
12	Selection of the „optimally describing Isoline“	*Example given for Excel*
	Preparation for comparisons and graphical presentation	*Query / Selection, Objects / Data aggregration / partition*

Step 1: Dataset (Shape-layer with sites as points)

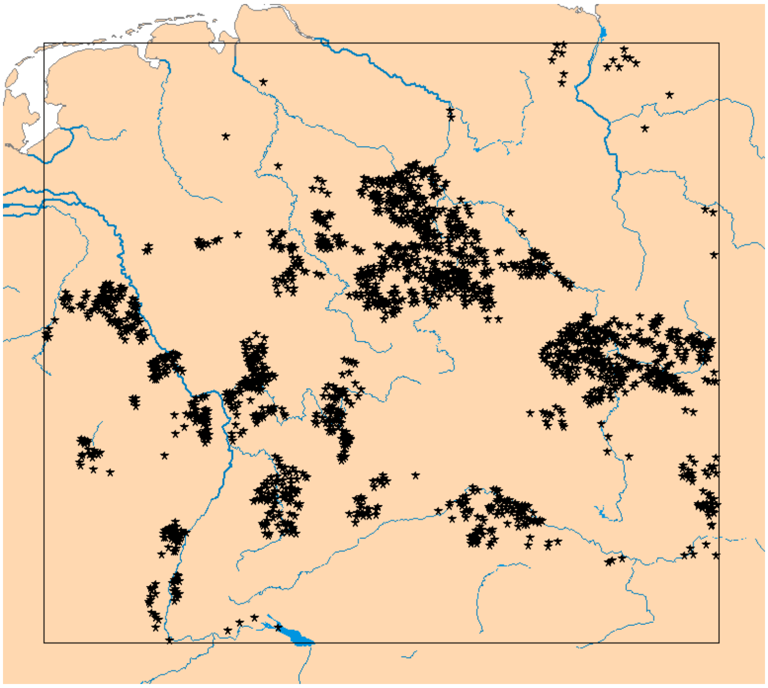

Our example data set consists of 2378 early Neolithic sites.

Start MapInfo and import the data set: 13_earlyNeolithic_CE_sites_GK3.shp

Convert Shape File to MapInfo Table and check the projection system of your sites-layer and your individual base map. Both should be EPSG 31467 (DHDN / 3-degree Gauss-Krüger zone 3). If they are not, change them accordingly.
Use Cosmetic Layer to create a rectangular map section around the sites. Use */Save Cosmetic Layer* to save the file as map_section.

In some instances, a rectangular map-section is meaningless for the dataset at hand, e.g. if major glaciers or marine areas are included. Alternatively, one might use one of the following options to define the outline of the map section:

1) Create buffers around sites. The buffer radius needs be chosen carefully, e.g. the largest nearest neighbour distance. In some cases, this approach can reduce edge effects during interpolation.
2) Use the option */Hull boundary width /User defined* in step 2 instead of */select region from map*.

The aggregation of sites can be another usefull step of data preparation (see Sup. Information in: Schmidt et al. 2020). The idea is to avoid superimposed site-locations, which result e.g. from coordinate imprecision or coarseness (generally to be checked beforehand) or coordinates for several excavation/localities within a site. For hunter-gatherer and neolithic case studies a coincidence point distance of 100 m has been used in several studies. Site aggregation follows the same procedure as described in Step 4: “Aggregation of vertices”.

For this manual we did not aggregate the sites of 13_earlyNeolithic_CE_sites_GK3 by 100 m (which would result in an aggregation from 2378 points to 1869 points).

Step 2: Creating Voronoi Polygons

With *Vertical Mapper/ Natural Neighbour Analysis/ Create Regions From Points (Voronoi)* we start the process of creating Voronoi polygons (VP) around the sites of the data table 13_earlyNeolithic_CE_sites_GK3. They serve to create spatial relations of the sites by delineating the maximum space available for each site.

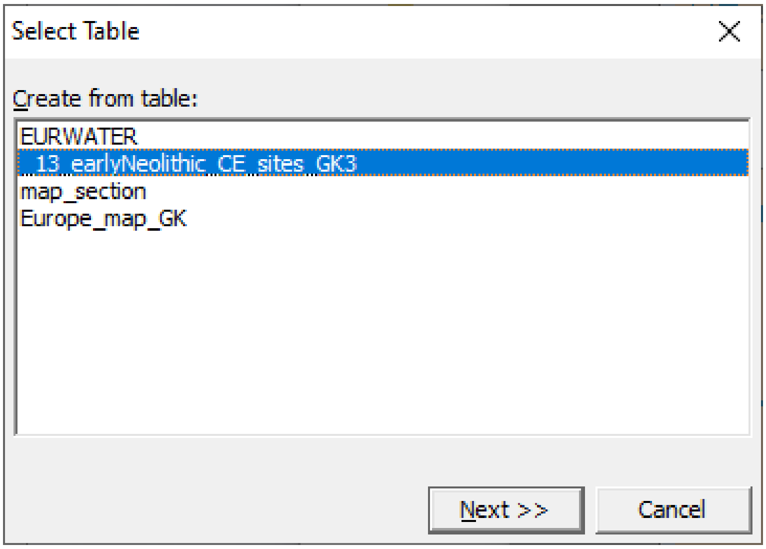

Select Table 13_earlyNeolithic_CE_sites_GK3
Press *Next* to continue.

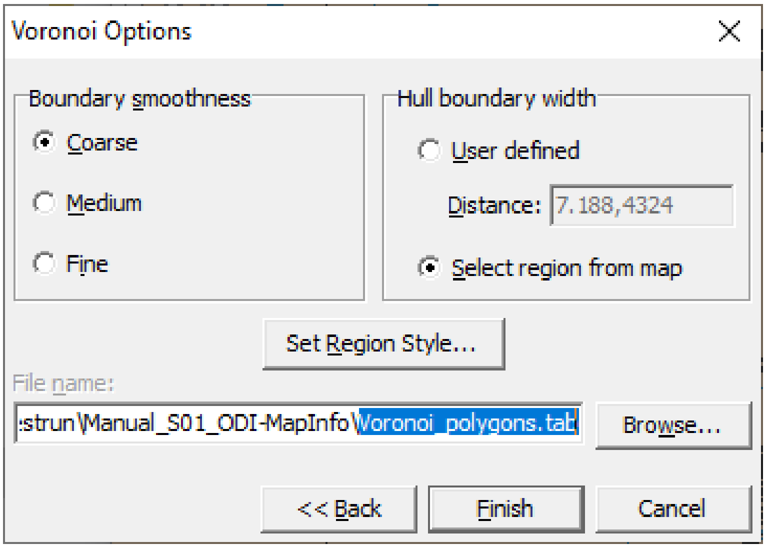

We chose from the next dialogue window

Boundary smoothness: *<u>Coarse</u>*

Hull boundary width: *<u>select region from map</u>*

We use *Browse* to open a window to determine the storage space and file name of the MapInfo table of Voronoi_polygons.
With *Save* we close the window and return to the previous one, which we also close with *Finish*.

Step 2: Creating Voronoi Polygons

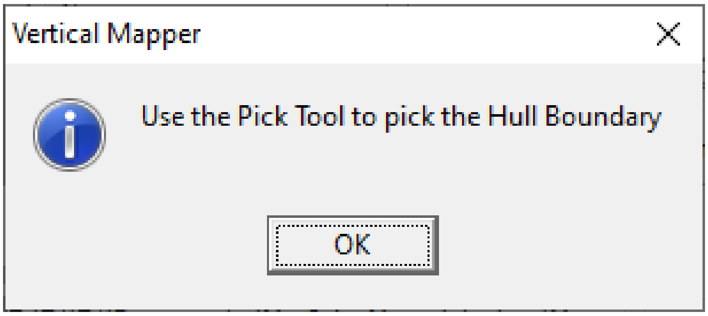

A dialogue box to use a Pick Tool appears, click OK. With the tool we click on the map and thereby select the map section frame as a boundary (Hull Boundary). Now a window with polygon lines should appear.

This window can be closed immediately and the new Table: Voronoi_polygons can be made visible in the *Layer control* by *Add* to the current map.

Visual output of result: Voronoi polygons
MapInfo Table: Voronoi_polygons

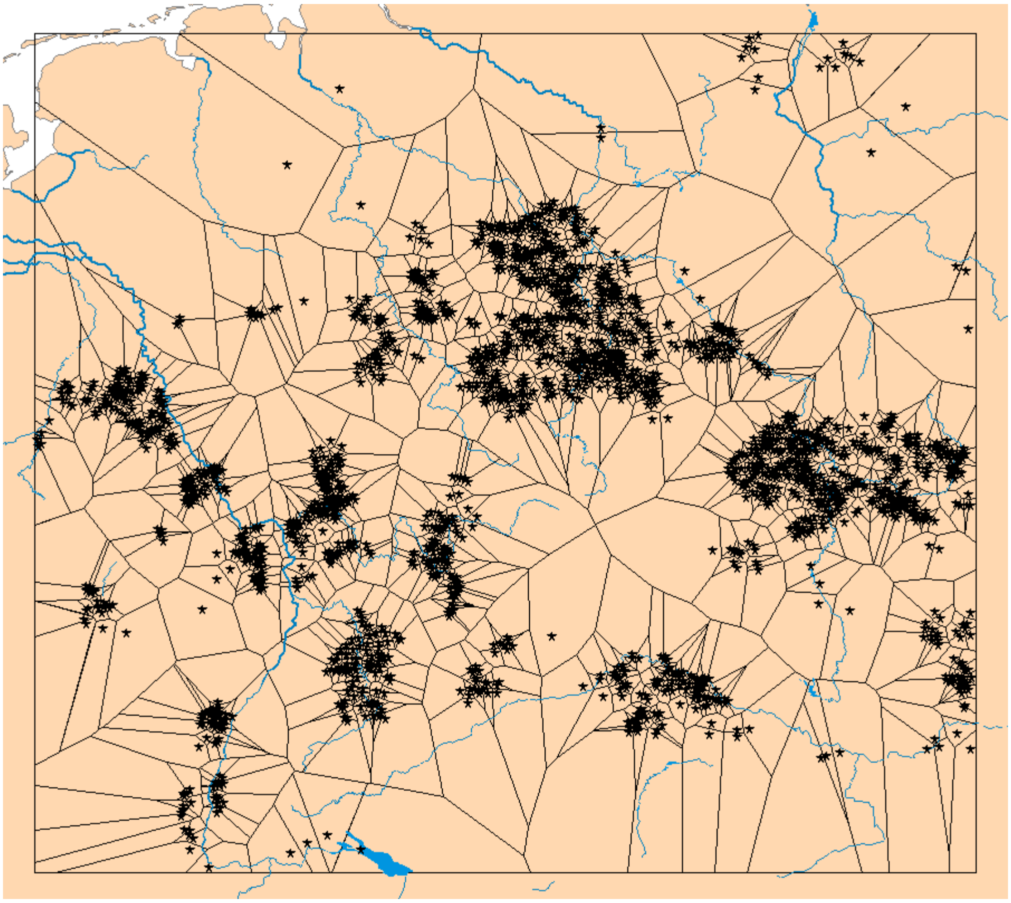

Step 3: Extraction of vertices

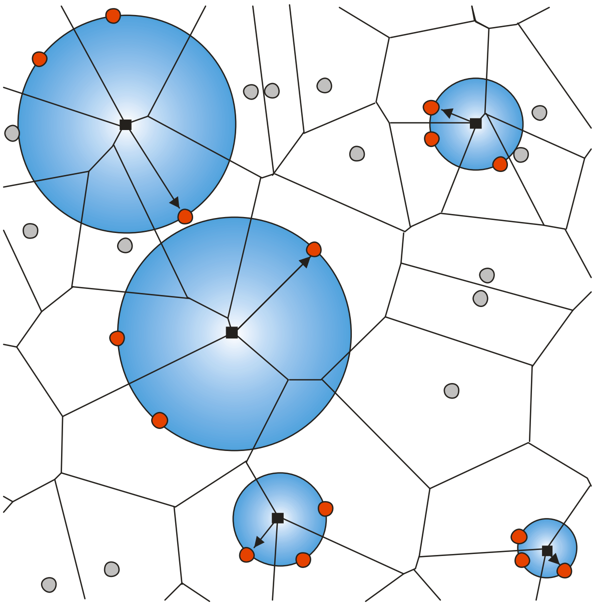

Figure by K.P. Wendt, after Preparata & Shamos 1988.

Excursus:

Each node of the Voronoi polygons constitutes the midpoint of an empty circle between three sites (Preparata & Shamos 1988, 256ff., 207, Fig. 5, 18). The radius of these Largest Empty Circles (LEC) is used as measuring value for the site density in the LUCIFS project.

To extract the nodes of the VP, we open a window with */Vertical Mapper /Create Grid /Poly-to-Point* , select Voronoi_polygons and set the options:
Extract from:
<u>Regions</u>
Distance between points:
<u>Use nodes only</u>
With *Browse* we open the dialog for saving the nodes.
The file is saved as Vertices_ptp.

The file automatically opens with cross symbols.

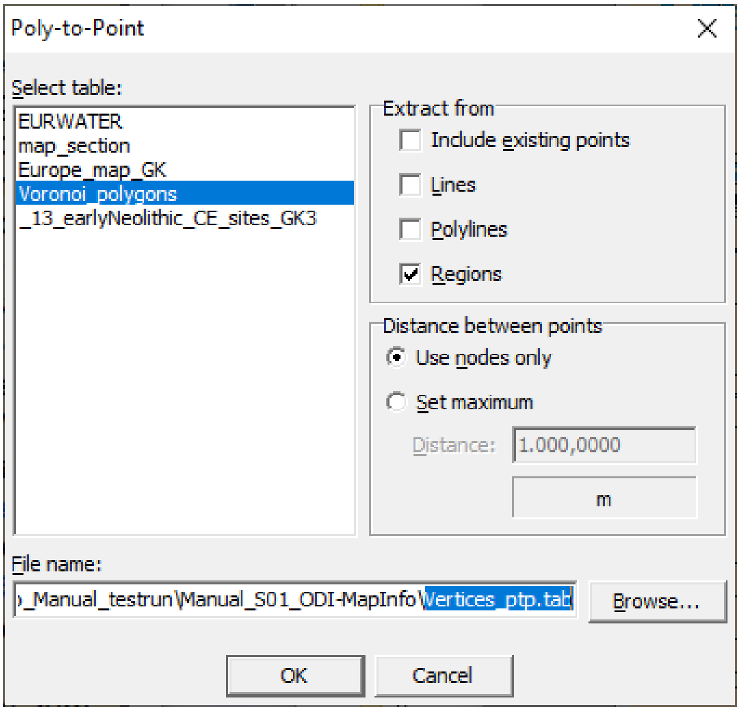

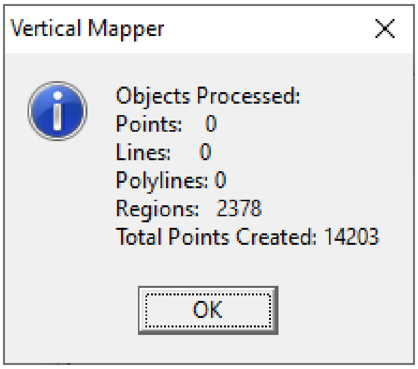

Step 4: Aggregation of vertices

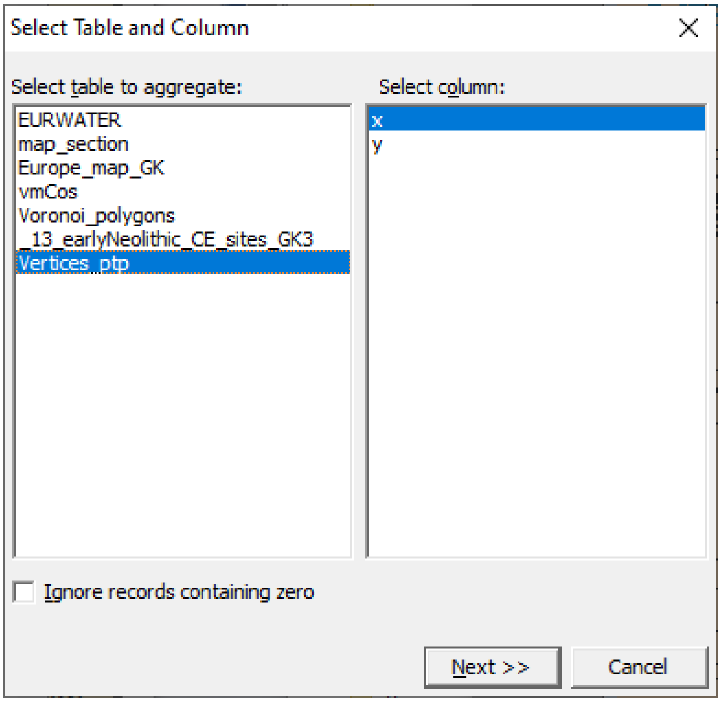

[If the table Voronoi_ptp contains no coordinates yet (x/y or Long/Lat), extract coordinates as explained on page 10 before proceeding.]

Several VP nodes are now doubled. All dopplers must be removed before proceeding, otherwise the radii for the LEC are calculated twice. */Vertical Mapper /Data Aggregation /Simple Point Aggregation* opens the corresponding menu. With *Select table to aggregate*:

We select Vertices_ptp. At *Select column:* one of the *coordinate columns of the table (x or y)* should automatically be selected, since the aggregate functions must refer to a column (if not, return and adjust Table structure manually under: */Table /Maintenance /Table Structure*). Press *Next* to continue to the next menu item.

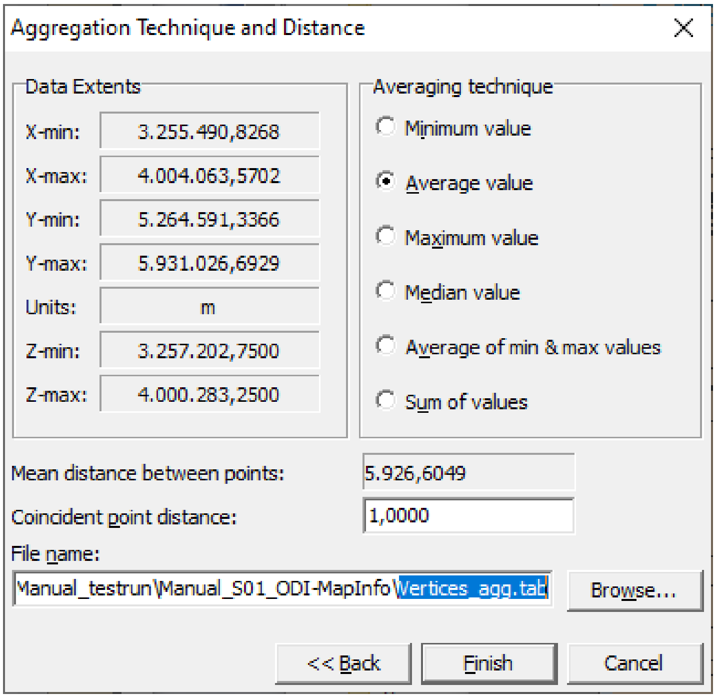

Select:
Averaging technique:
Average value and
Coincident point distance: 1

The data of points lying on top of each other are averaged for the column selected in the previous window (in our case, the coordinate values which are only one meter apart).

With *Browse /Save Table As* we save the Table of aggregated VP nodes as Vertices_agg. Continue with *Finish*.

Step 4: Aggregation of vertices

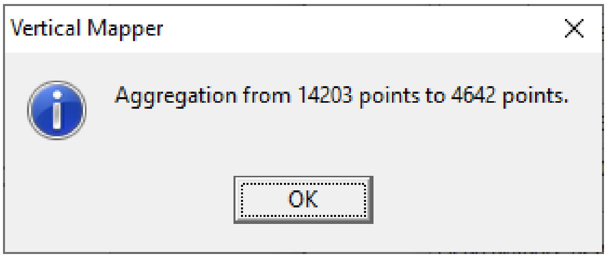

A pop-up window shows us the number of nodes transferred to the new MapInfo Table. We confirm the window (*OK*). A map window with points opens (these are the VP-nodes). We close that window. In our original map window we open the *Layer control* and with *Add* we choose the MapInfo Table Vertices_agg.

We have now created the center points of the LEC (blue crosses on the map).

Before proceeding we need to extract coordinates of the VP nodes because so far they only exist as a position in the map window.

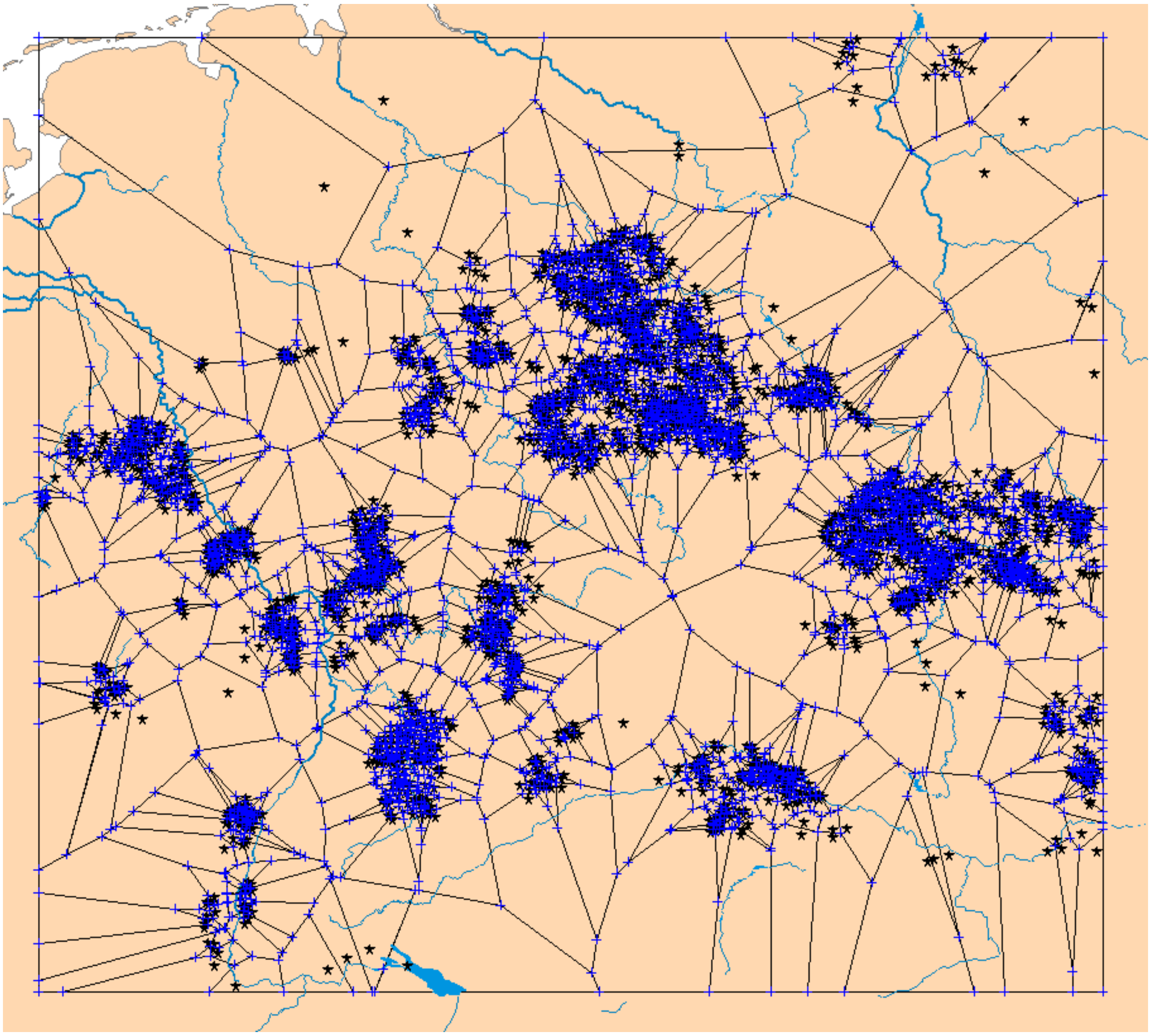

Step 4: Aggregation of vertices

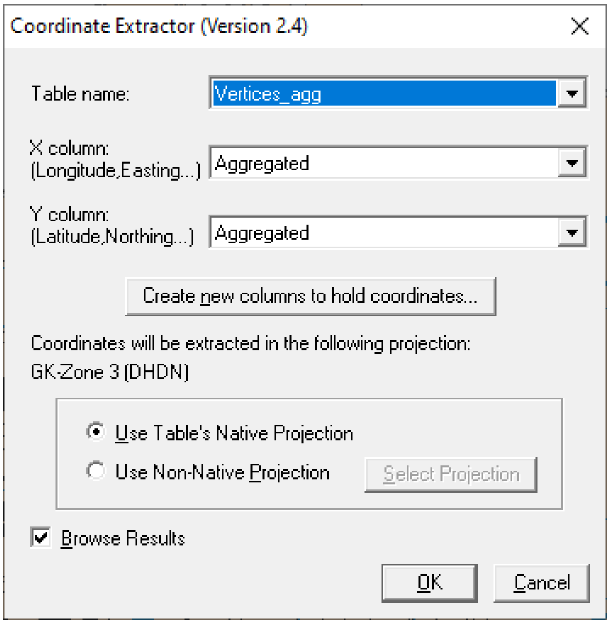

For further calculation, coordinates of each vertice need to be extracted. With */Tools /Coordinate Extractor /Extract Coordinates* the corresponding coordinates are assigned; we select the *Table name* Vertices_agg.

Leave all other fields unchanged, instead press the button *Create new columns to hold coordinates*.

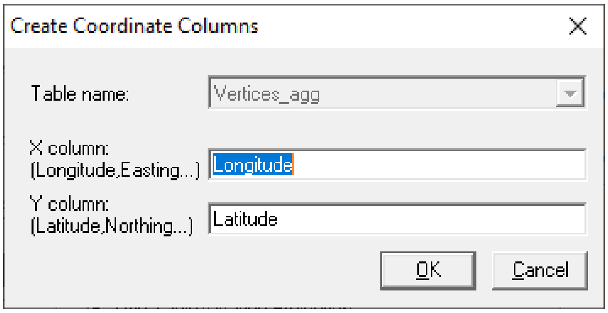

In the following dialogue window we leave the column names as proposed, then we proceed with *OK.*

Before proceeding, we also need to add an *ID* column to Vertices_agg

Select */Table /Maintenance /Table Structure* and add a field with the name *ID* for the new column. The new column can be any numeric type (preferably chose the same type as for column *_id* in Table 13_earlyNeolithic_CE_sites_GK3.shp since they need to correspond later anyway).

Update Column

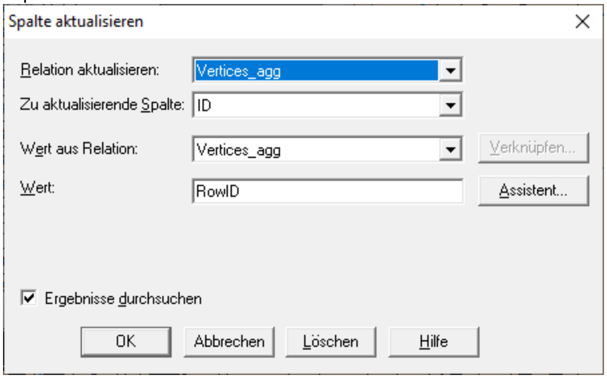

Then select */Table /Update Column* and chose
Table to update: Vertices_agg
Column to update: ID
And enter *Value*: <RowID>

Proceed with *OK.*

Step 5: Defining the radius of the „Largest Empty Circle“

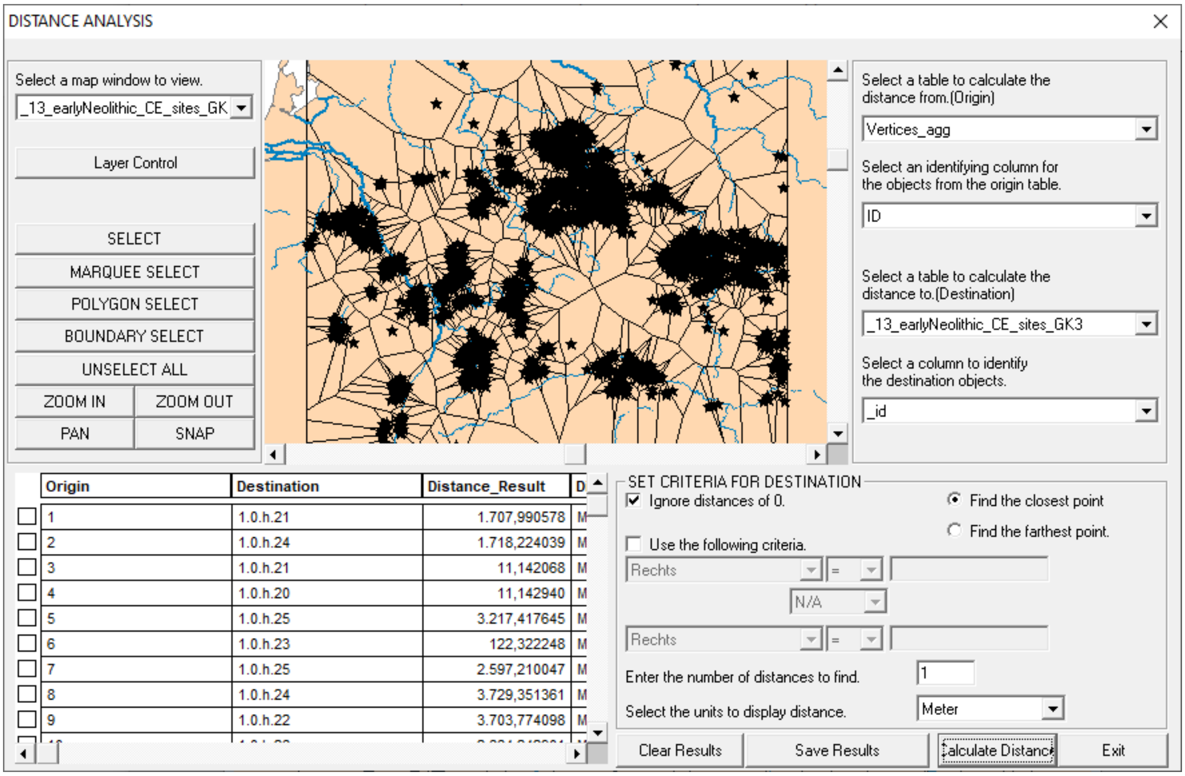

Now we measure the radius of the LEC by calculating the nearest distance between neighbouring vertices (Vertices_agg) and sites (13_earlyNeolithic_CE_sites_GK3). We start with *Tools /Distance Calculator /Run Distance Calculator* and select the MapInfo Tables and ID columns:

Origin: Vertices_agg, *ID*: *ID*
Destination: 13_earlyNeolithic_CE_sites_GK3, *ID:* *_id*
(Note: the format of the ID-columns must be identical in both tables, e.g. either *decimal,* or *floating point, or* ….).

We keep the presetting of
> *Ignore distances of 0*
and
> *find the closest point*
Enter the number of distances to find: 1
Select the units to display distance: Meter

We start the procedure with
„*Calculate Distance*“

Finally, we select „*Save Results*“ and save the Table as „lec_radii“.
Press *Exit* and open the new Table.

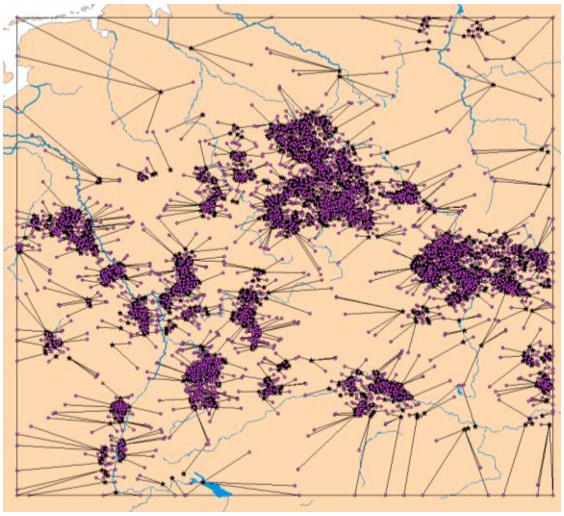

Step 5: Defining the radius of the „Largest Empty Circle“

To transfer the radii values into the table Vertices_agg, we select Vertices_agg with */Table /Maintenance /Table Structure,* and add one new column in the MapInfo Table (here: *Radii*), select *decimal* and enter “*20*” for *“Width”* and “*6*” at *decimal place*. Change *ID* to *character (“Zeichen”)* and enter “*30*”, identical to the column *Origin* in lec_radii. Press *Ok* and return to map.

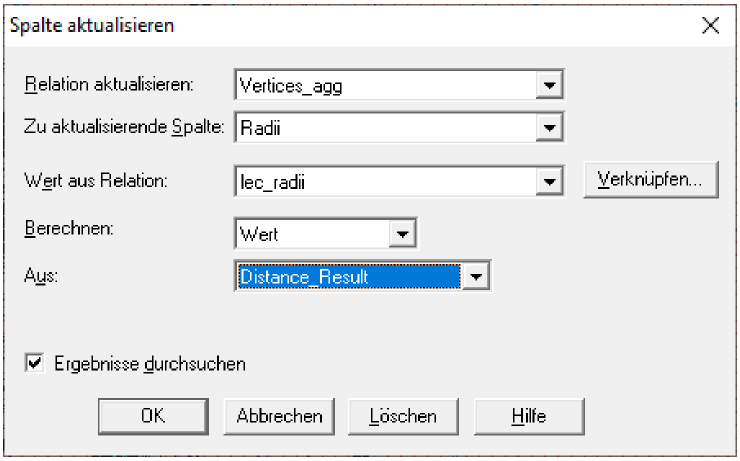

Then we select */MapInfo Tabel /update column* and select

Table to Update: Vertices_agg
Column to update: *Radii*
Get Value From Table: lec_radii
Calculate: *Value („Wert“)*
From („Aus“): *Distance Result*
(*which is a preset name given by the Distance Calculator for the output table*).

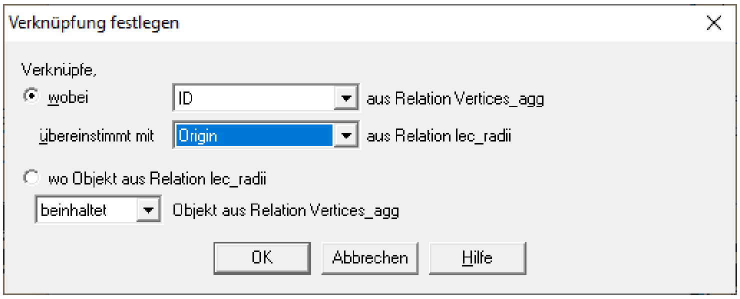

Press „*Connect*“ (*Verknüpfen*) and define the columns connected.
(for lec_radii this is the column *Origin* which was created by the Distance Calculator; for Vertices_agg it is *Origin*).

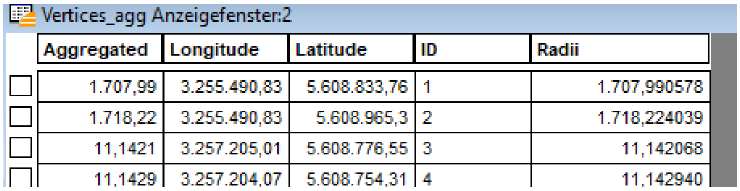

Vertices_agg Anzeigefenster:2

Aggregated	Longitude	Latitude	ID	Radii
1.707,99	3.255.490,83	5.608.833,76	1	1.707,990578
1.718,22	3.255.490,83	5.608.965,3	2	1.718,224039
11,1421	3.257.205,01	5.608.776,55	3	11,142068
11,1429	3.257.204,07	5.608.754,31	4	11,142940

Press *OK* to finish and inspect the values.

The column *Radii* of table Vertices_agg now contains the distance values between the VP nodes and sites, i.e. the measured radius of the LEC. At this stage, they are still point data that need to be interpolated to surface data in the next step.

Now we have to use Kriging to interpolate the *Radii* point data into surface data.

Step 6: Kriging – Preparation and Grid

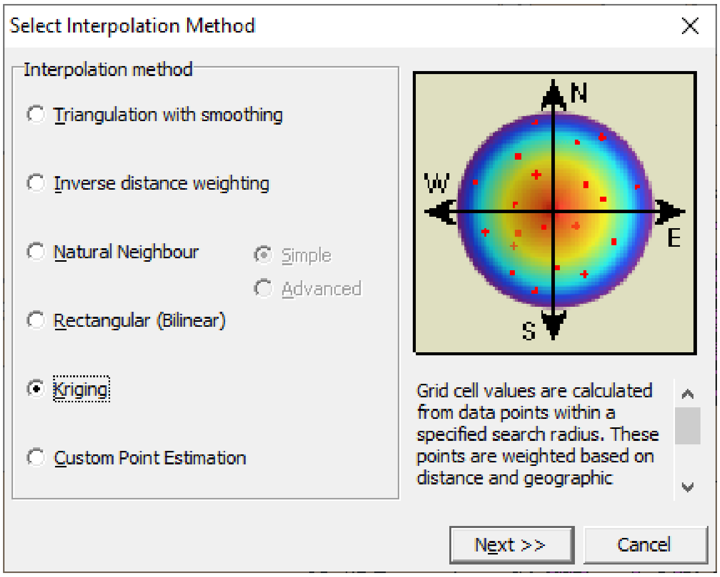

With */Vertical Mapper /Create Grid /Interpolation* we start the interpolation of the point data in order to identify areas with the same low-density limit.

In the dialogue window that opens, we mark *Kriging* and press *Next*.

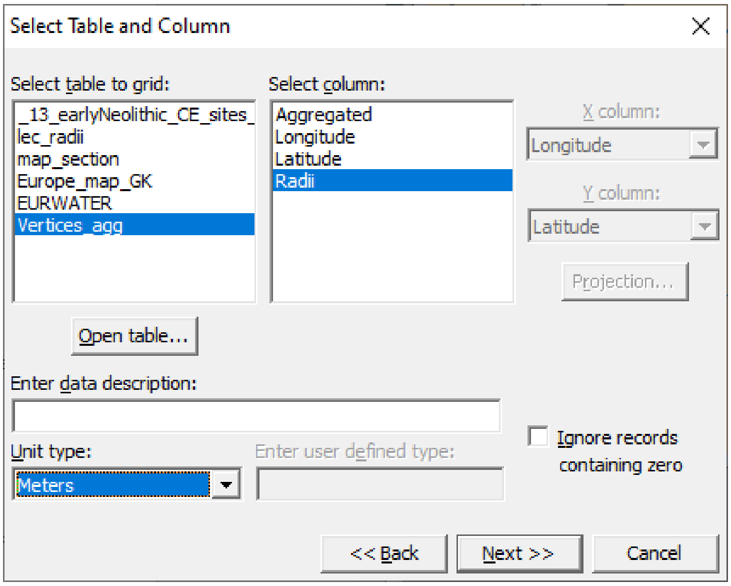

In the following dialogue window select the LEC values:

Select table to grid: Vertices_agg
Select column: *Radii*
For *Unit type* we select *Meters*.

On the right hand side of the window, the columns for the coordinates should be correctly preselected by the program. Otherwise they need to be rearranged by changing the table structure.

Press *Next*.

Step 6: Kriging – Preparation and Grid

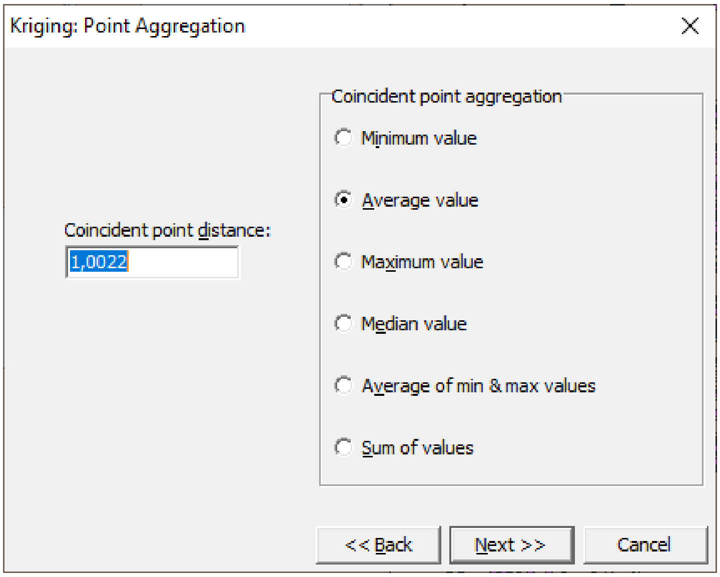

The pre-set value in the following window indicates the distance between two sites, at which the program aggregates both sites into a single one. The program also offers the possibility to select how the coincident point aggregation should be conducted. Here we leave the pre-setting and we proceed with *Next*.

Now we can set the *Cell size* of the grid cells (*<u>100m</u>*) [for continental scales, we select *<u>1000m</u>*], other default settings can be adopted. We use *Browse* to open a window to determine the storage space and file name of the MapInfo table to kriging_lec_radii.tab. Then we press *Variogram builder*.

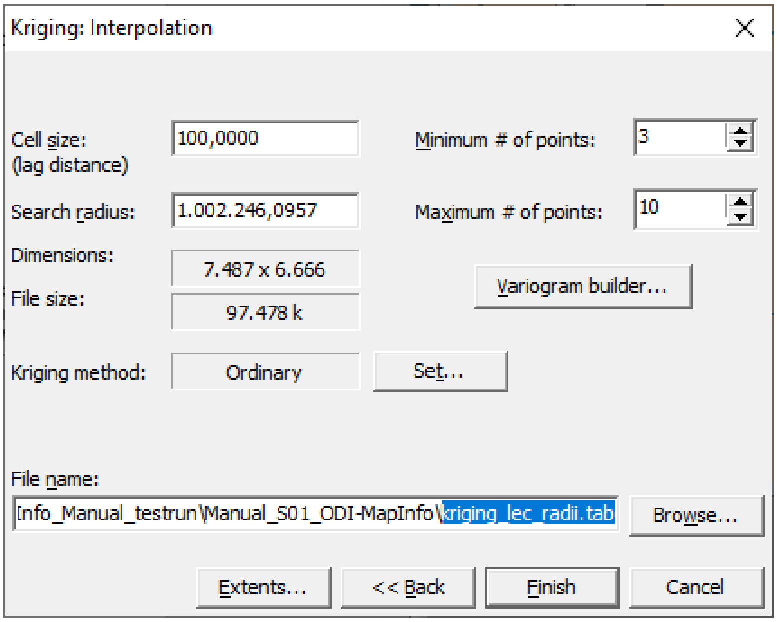

Step 7: Kriging – Semivariogram

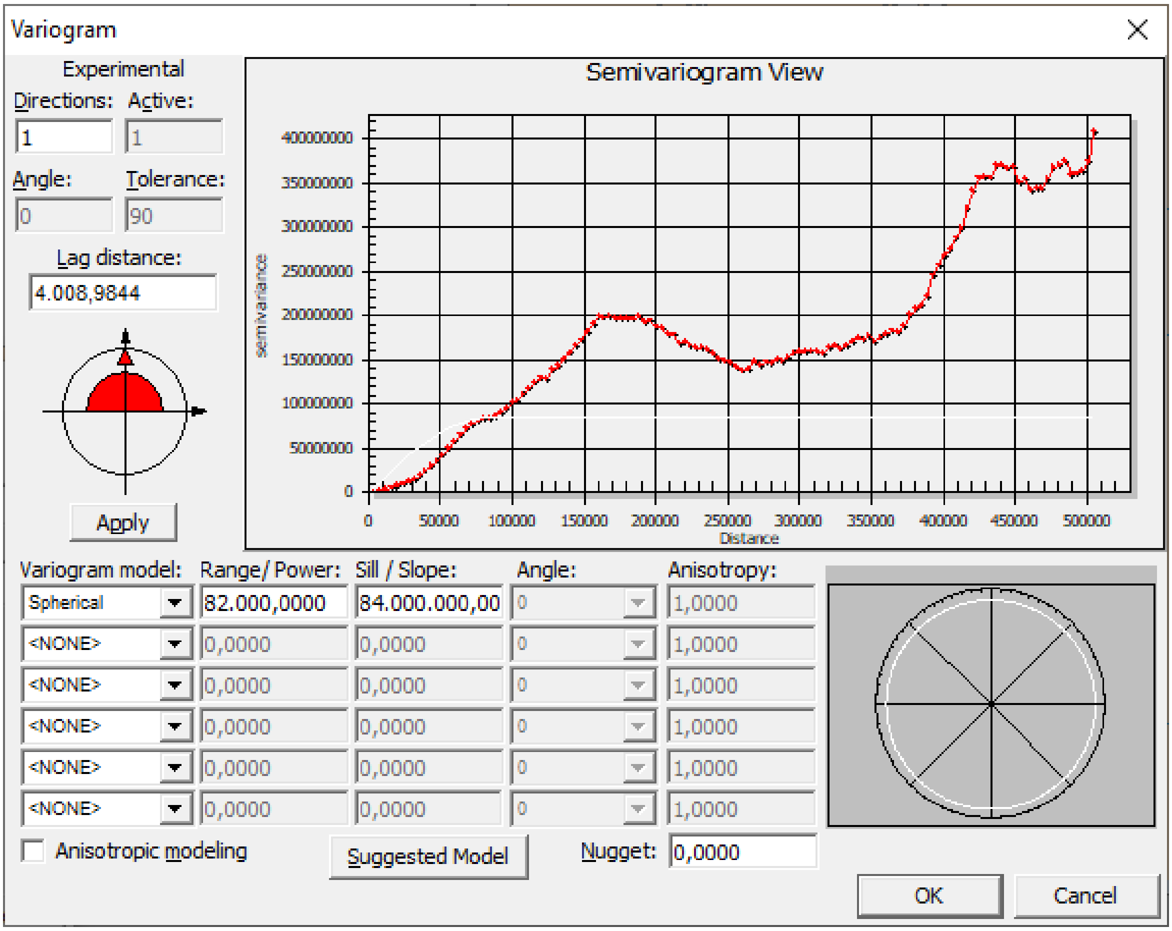

The Semivariogram shows the relationship between the investigated value and the distance between measured points. It allows to weight the data using the following settings:
For *Experimental /Directions*: we change the value from 2 to 1.
Press *Apply* to accept the change.

Then we choose for the *Variogram model: Spherical*. Set *Nugget* to *0*.

The remaining curve visualizes the relationship between point distance (*x-axis: Distance*) and the degree of scatter of the investigated values (*y-axis: semivariance*). We search for the first data plateau (*in this example the x-axis value would be*: 82.000 [*Range*]; *and y-axis value*: 84.000.000 [*Sill*]). We enter both values in the corresponding window *Range/Power* and *Sill/Slope*.

To facilitate the search for the first plateau, we can enlarge the Semivariogram View by clicking right on the diagram, select *Maximize* and zoom in.

Step 7: Kriging – Semivariogram

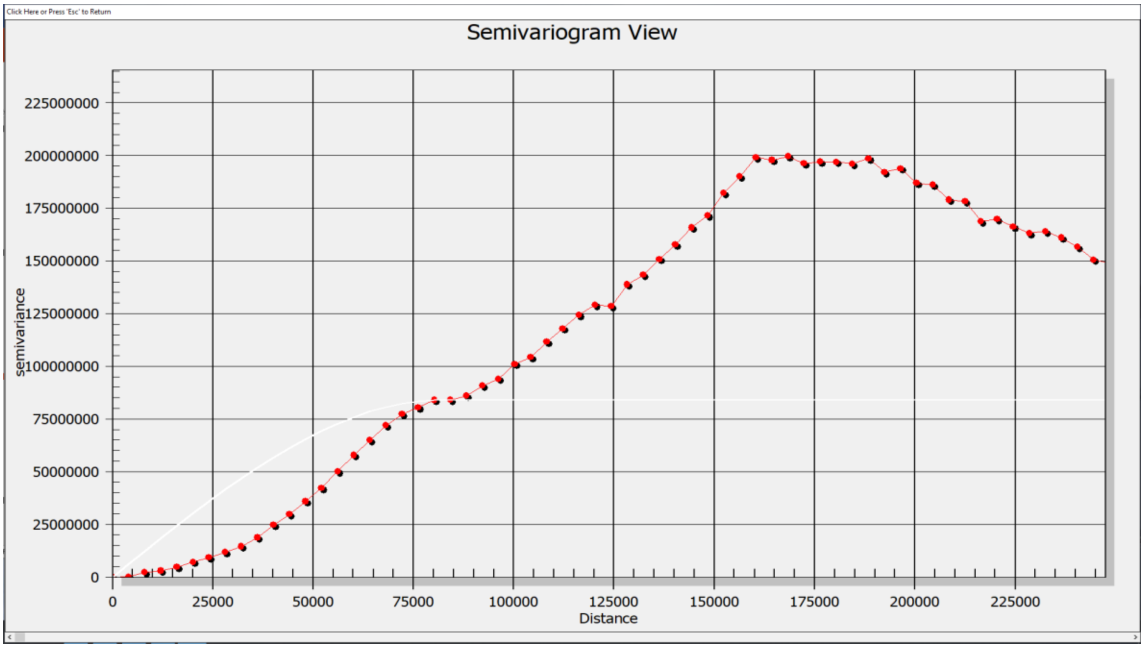

The maximised Semivariogram Views should look like this. With *OK* we return to the previous window and press *Finish*.

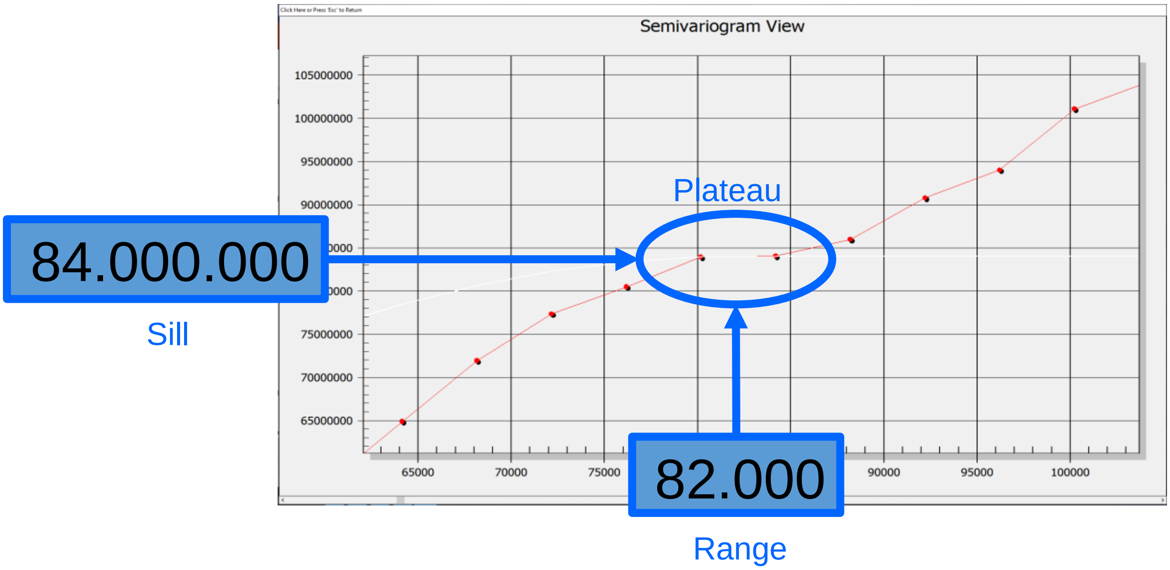

…

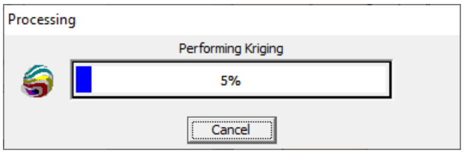

…

Step 8: Kriging – inspect and export raster output

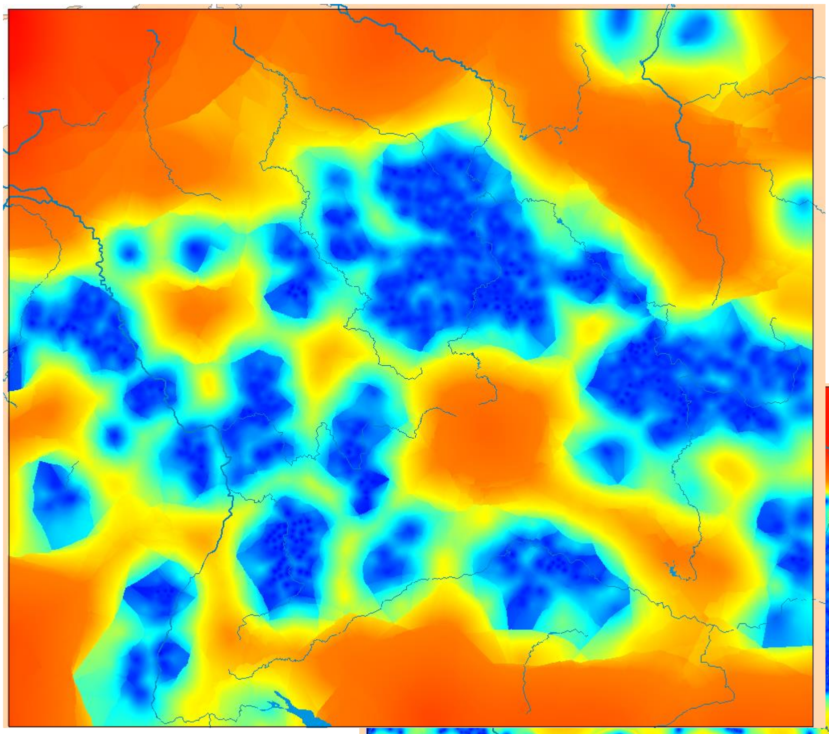

kriging_lec_radii.grd

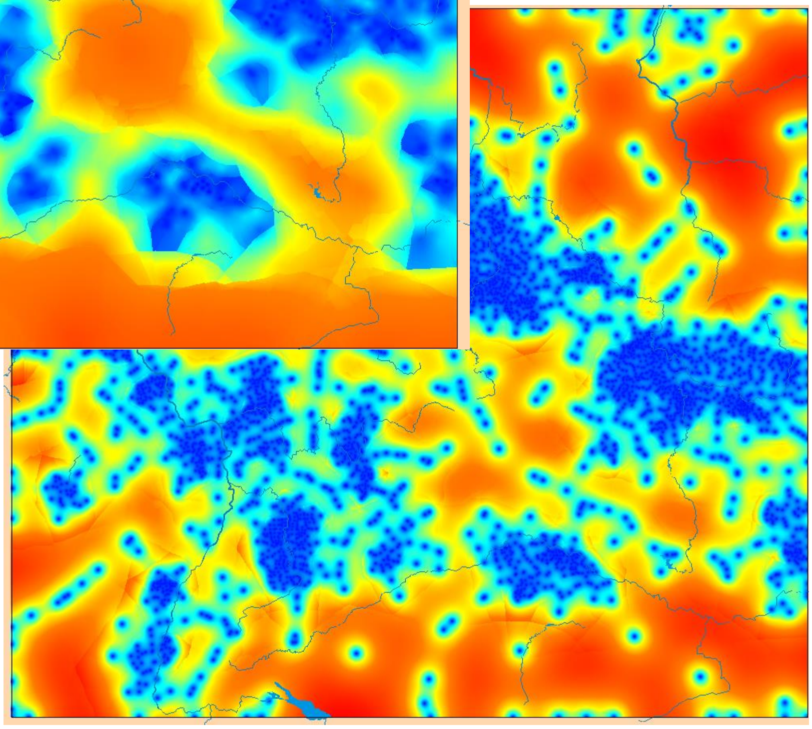

kriging_lec_radii_var.grd

As a result, two raster images are stored in the designated file. The left image shows the interpolated LEC radii (kriging_lec_radii.grd). The right image displays the variance of the data (kriging_lec_radii_var.grd). The right image is only used to check for areas on the map where interpolation is safe (*blue*) and where it is not (*red*), e.g. to inform the contour of a clipping mask or to identify edge effects after interpolation.

For the next steps we only need the left grid file with the LEC data. We close the right grid file with the variance values.

In cases of a map section that encompasses coastlines or glaciers we recommend to use the function */Grid Manager /Tools /Trimmer* to cut off these areas before interpolation.

Step 9: Creating contour lines (Isolines)

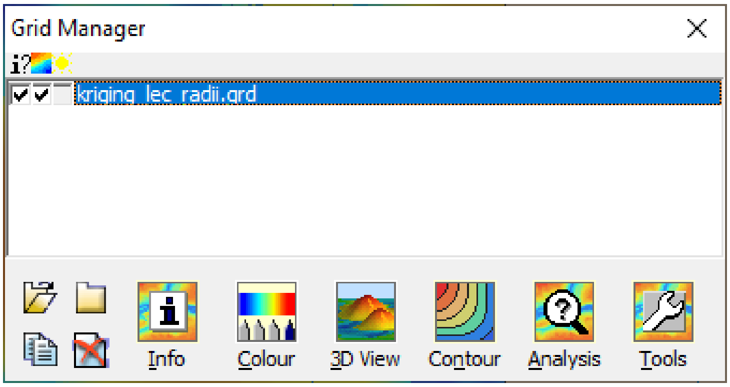

With *Grid Manager /Contour* we start creating isolines.

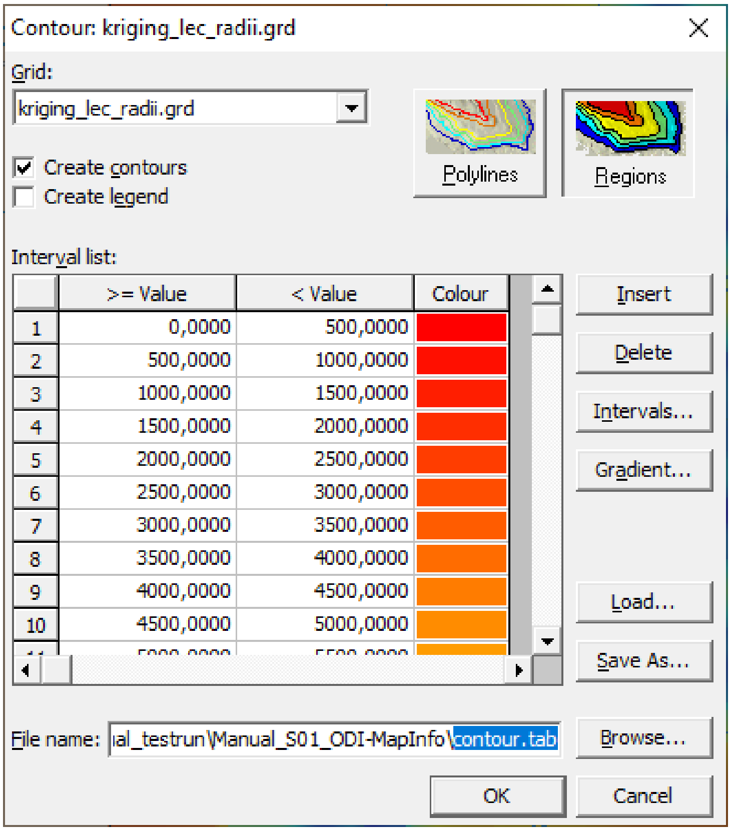

Activate the *Regions* button in the following dialogue window.

Then use *Browse /Save Contour as*, select folder and save the file name as contour.

Open *Intervals* and set the value range (*Minimum* = *0*, *Maximum* = *25.000m*) and the *Interval Value* (= *500m*).

[for continental scales, we select a higher *Maximum* value, e.g. *100.000m*, and *Interval Value* = *1.000m*]

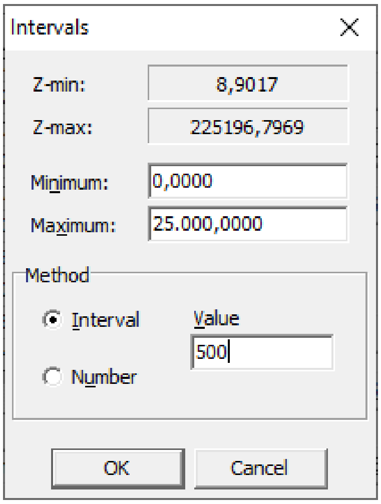

The *minimum* should be set to "*0*" in order to maintain even interval limits.

The *Gradient* can be set individually and will influence the visibility of the areal increase in the isolines. For better visibility, you can chose here to *flip colours*

Step 9: Creating contour lines (Isolines)

Result:

Isolines of site density
(*density measure: Largest Empty Circles*)
MapInfo Table:
contour

The result should look like the image below. (MapInfo sometimes requires to save contour as a copy before proceeding. We name the copy contour_, close contour and continue).

In order to find the Optimally Describing Isoline *(in the sense of Core Areas*), further data must now be compiled and processed in MapInfo and evaluated in Excel *(this is demonstrated using area sizes of the isolines, and number and percentage of enclosed archaeological sites as an example*).

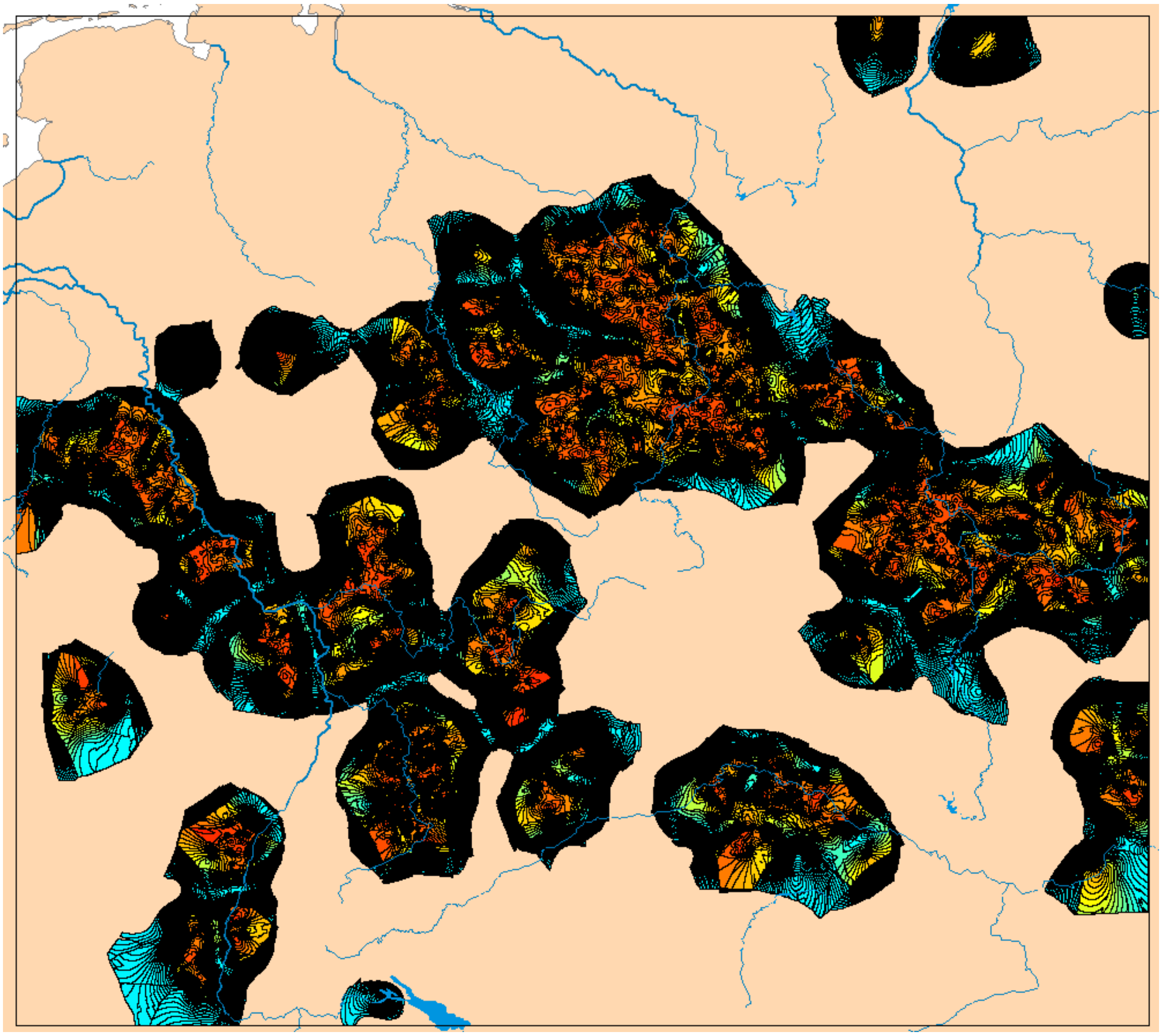

Step 10: Calculating the area and the number of sites per isoline

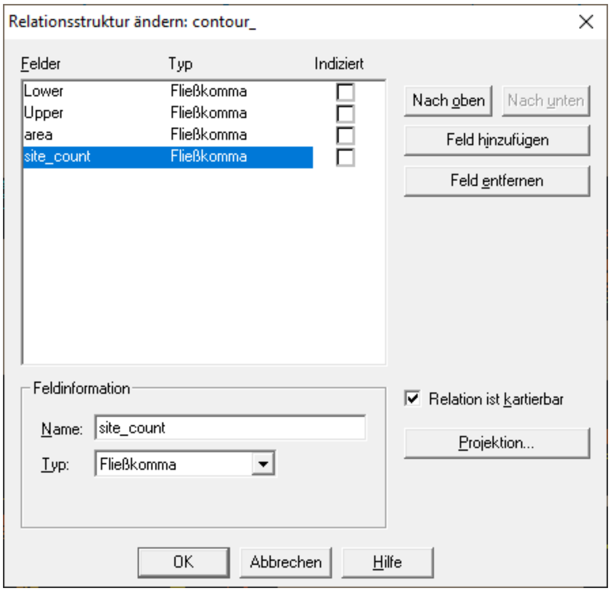

With */Table /Maintenance /Table Structure* two new columns are created in the MapInfo table contour_. To do this, the MapInfo table is marked in the automatically opening dialogue window and selected with *OK.*

In the following dialogue window (left) the MapInfo table structure is displayed with two columns, whereby *Lower* contains the lower, *Upper* the upper interval limit of the respective isoline. With the button *Add field* two new columns in *floating point* format are created with the names *area* and *site_count*. Depending on the format, the number of permitted characters may also have to be specified. Press *OK* to start the process.

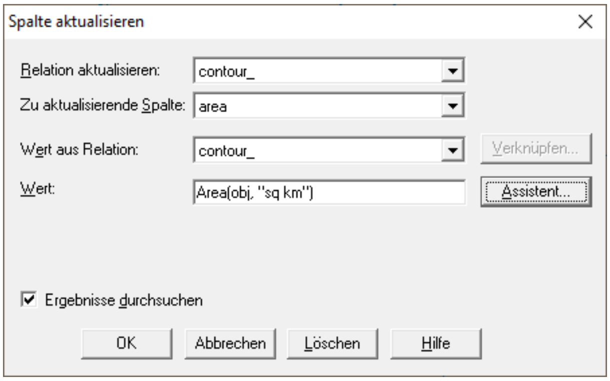

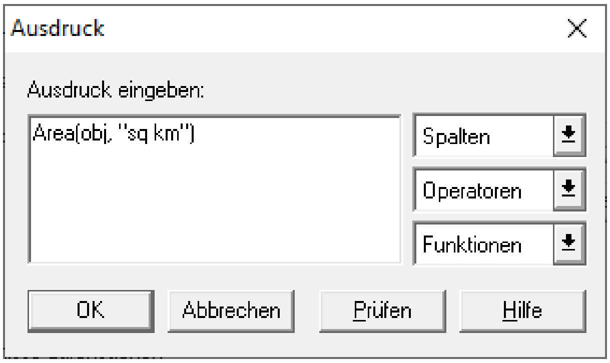

With */Table /Update column*, new values are now calculated for the column *area*. The dialogue window allows the selection of the MapInfo table (*Update relation:* contour_) and the column (*Column to be updated: area*). Press button *Assistant.* You can now use three input fields to construct a formula or choose between different functions. Here only the functions field has to be activated and select from the range of different functions *<area>*. The default calculates km^2 (*sq km*), the value can be changed in the function by clicking on it. With *OK* we return to the previous window and start the calculation with *OK*. If we let the preset checkmark on the menu item *Search results*, the Table opens in a new window.

Step 10: Calculating the area and the number of sites per isoline

To count numbers of site per isoline interval, we use */Table /Update column*.
New values are now calculated for the column *site_count*.

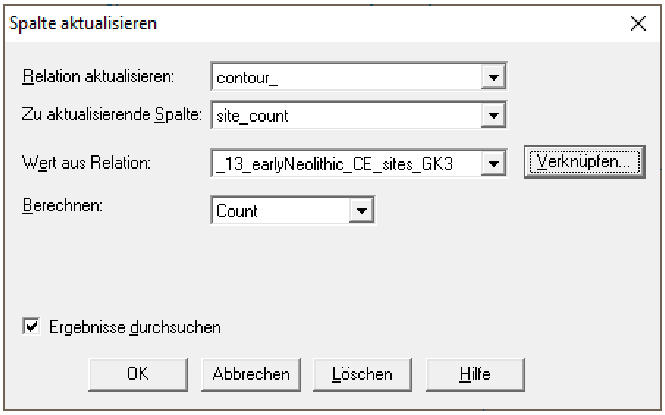

The dialogue window allows the selection of the MapInfo table (*Update relation*: contour_) and the column (*Column to be updated: site_count*). Take *Value from Table*: 13_earlyNeolithic_CE_sites_GK3. *Calculate: Count*

We start the calculation with *OK*.

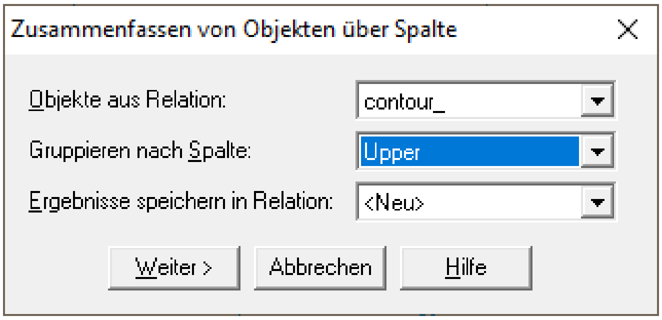

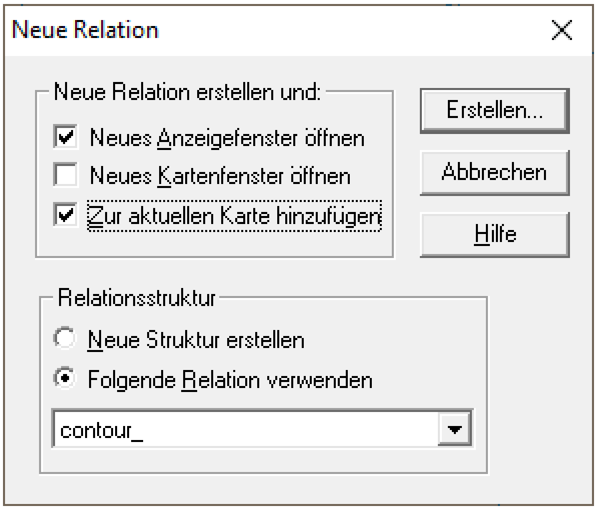

To sum areas of the same isoline intervals we open MapInfo Table contour by */Table /Sum objects /Connect via column* and select the column *Upper* in *Group by column*. Save results as *<New>* (default). Press *Next.*

The dialogue offers settings: We tick *Open new display window*, and *Add to current map*. We leave the settings under *Table structure* as default and start the process with *Create*.

The *New Table Structure* dialogue appears. Changes can still be made here (*e.g. in the formats*). Press *Create*. The window for saving opens automatically and requires to specify storage file and name (contour_sum).

Step 10: Calculating the area and the number of sites per isoline

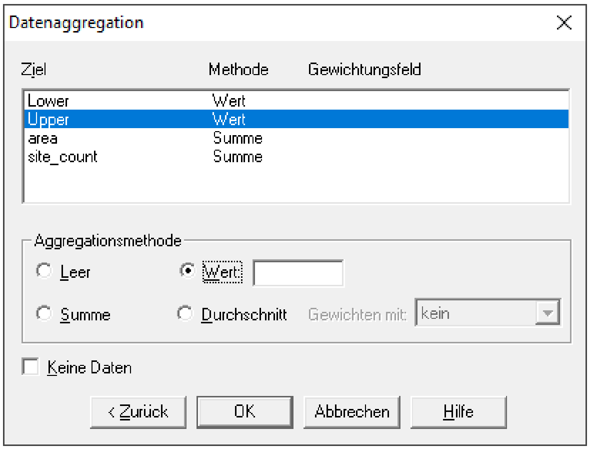

The next window asks how to summarise the column values. For *Lower* and *Upper*, <*Value*> has to be chosen (*click*) without further specifications, since the interval limits remain unchanged. For *area* and *site_count* chose <*sum*>. Press *OK* to start calculation.

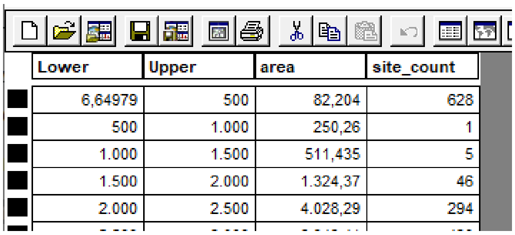

Lower	Upper	area	site_count
6,64979	500	82,204	628
500	1.000	250,26	1
1.000	1.500	511,435	5
1.500	2.000	1.324,37	46
2.000	2.500	4.028,29	294

Open the table of the file *contour_sum* and control the results of the summarizing process (columns *area* and *site_count*).

Step 11: Data export

With */Table /Export* we create an Ascii file that is imported to Excel for further processing. After selecting the name and the storage location, *Delimited Ascii (txt)* must be selected in the _File type_ field. In the next dialogue window, select *Tabulator* as the delimiter, *ANSI* as the character set and the first line of the data set should serve as the column heading.

For the next step we start MS Excel and with *File /Open* we import the Ascii file into the spreadsheet. In order for Excel to recognize the file, the *file type* must be set to *text files*.

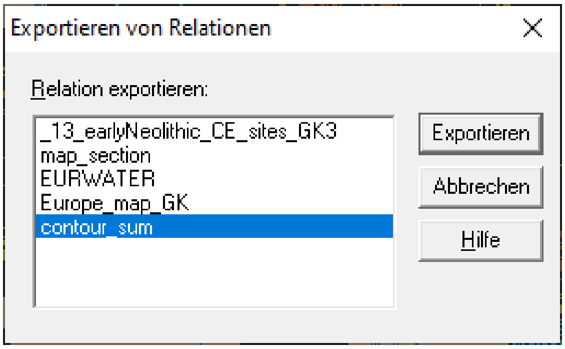

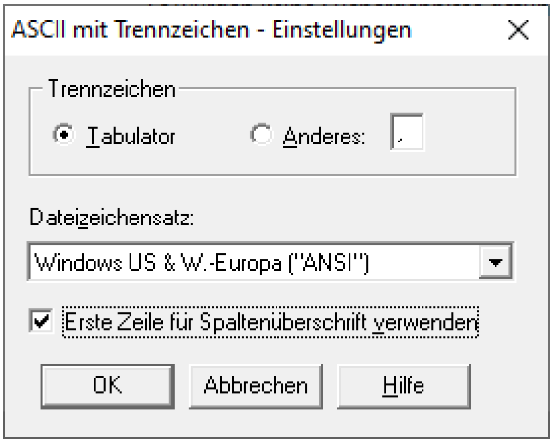

Step 12: Selection of the optimally describing Isoline

"Following the recommendations of Zimmermann et al. (2004, 53f.) and Zimmermann et al (2009, 9ff.) three statistical properties are of interest: the difference in increase of sites per equidistance, the number of areas with a specific site density and the increase of included space" (Broich & Peters 2020). The most important is, however, the increase of included space (Zimmermann et al. 2009, 9) detailed below. For the others, we refer to the R-manual (GitHub repository CologneProtocol-R: https://github.com/C-C-A-A/CologneProtocol-R).

	A	B	C	D	E	F
1	Lower	Upper	area	site_count	site_cumul	site_perc
2	7	500	82	628	628	26,7
3	500	1000	250	1	629	26,7
4	1000	1500	511	5	634	26,9
5	1500	2000	1.324	46	680	28,9
6	2000	2500	4.028	294	974	41,4
7	2500	3000	6.349	430	1404	59,6
8	3000	3500	7.152	302	1706	72,5
9	3500	4000	7.279	207	1913	81,3
10	4000	4500	7.312	109	2022	85,9
11	4500	5000	6.547	66	2088	88,7
12	5000	5500	6.007	60	2148	91,2
13	5500	6000	5.654	37	2185	92,8
14	6000	6500	5.469	24	2209	93,8
15	6500	7000	5.218	27	2236	95,0
16	7000	7500	5.092	24	2260	96,0
17	7500	8000	4.702	15	2275	96,6
18	8000	8500	4.506	13	2288	97,2

Copy-paste the data from the .txt file into a new Excel sheet. Make sure data and formatting are correct (*Lower* and *Upper* are in MapInfo the min/max values of the contour lines). Add the following columns:
sites_cumulative
sites_percentage

Calculate the cumulative number of sites by adding the newly enclosed sites to the previous summed ones (e.g.
Enter in E2: =D2
enter in E3: =E2+D3
enter in E4: =E3+D4 etc.)

Then calculate the percentage of sites from the cumulative sites in relation to the total number of sites (2354, e.g.
Enter in F2: =E2*100/2354
Enter in F3: =E3*100/2354 etc.)

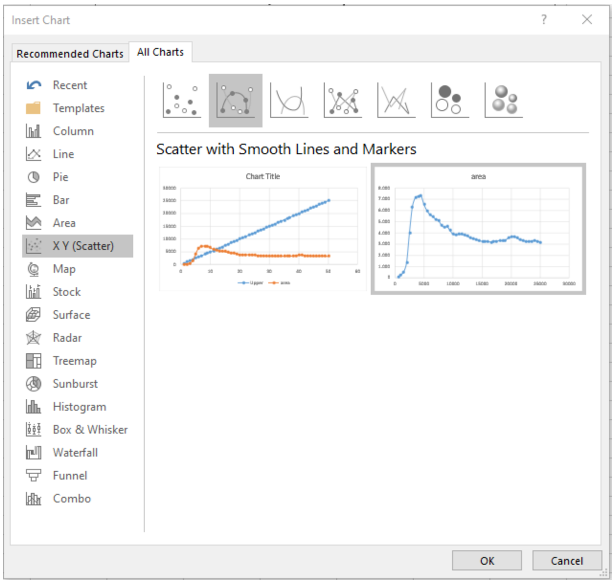

With */Insert /Diagram* we now create a graph that should make it easier for us to select the Optimally Describing Isoline based on the increase in area. Select the respective columns (e.g. *Upper* and *area*; or *Upper* and *site_percent* respectively) and chose *Scatter with Lines* as the *diagram type*. Press *Next*.

Step 12: Selection of the optimally describing Isoline

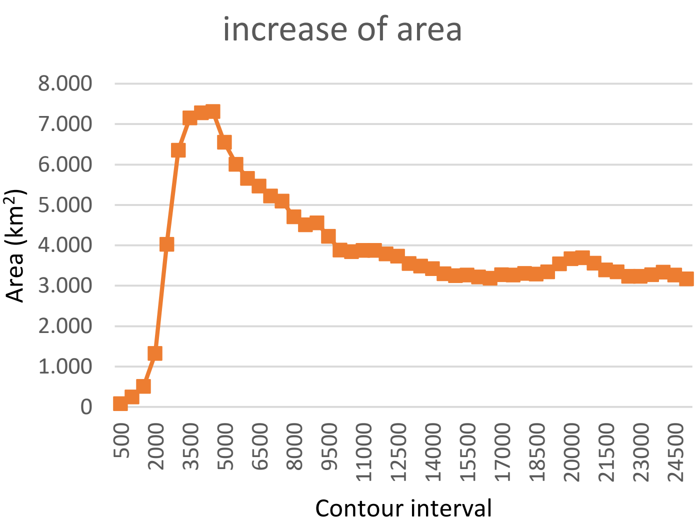

The diagram on *area* shows one maxima. Other maxima – which occur in a range that was not economically interesting for a settlement – are ignored. Also check the number of sites per contour, as well as the number of individual Core Areas (see R-Manual and Schmidt et al. 2020). If the curve rises steadily, the data-set is not suitable for defining Core Areas (*e.g. if too few sites are recorded in the map section*).

In our example, **the Optimally Describing Isoline is at the local maximum of 4 km**. The sites/settlements in this area are at maximum 8 km apart (*2 x radius of the LEC*). We now save the Excel file and leave the program.

Set into its original prehistoric economic context, half of this distance, i.e. the LEC radius of 4 km, does capture a reasonable walking distance from a settlement to a field.

Preparation for comparisons and graphical presentation

Now we return to MapInfo to create a corresponding map with the Core Areas of the sites, which is then ready for further evaluation. We keep the value of 4 km in mind.

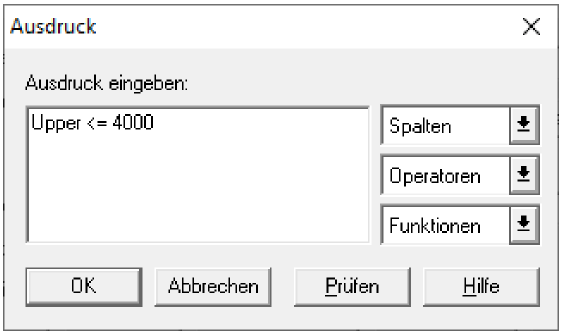

With */Query /Selection* we select the isolines from the MapInfo Table contour_sum: the upper limit of which is less than or equal to 4000 m (Upper <= 4000). With *OK* we start the selection and use */File /Save copy* to save the temporary MapInfo table as contour_sum_ODI_4km.

We load the newly created file and mark it as editable in the *Layer control* to perform some cosmetic operations. Firstly, we remove the different isoline intervals below 4000 m. With */Query /Selection* we select the MapInfo Table, remove all selection criteria and the check mark at *Show results*. Then we mark all isolines with the mouse or with */Query /Selection*. With */Objects /Summarize* the individual intervals will be summed into one object.

Preparation for comparisons and graphical presentation

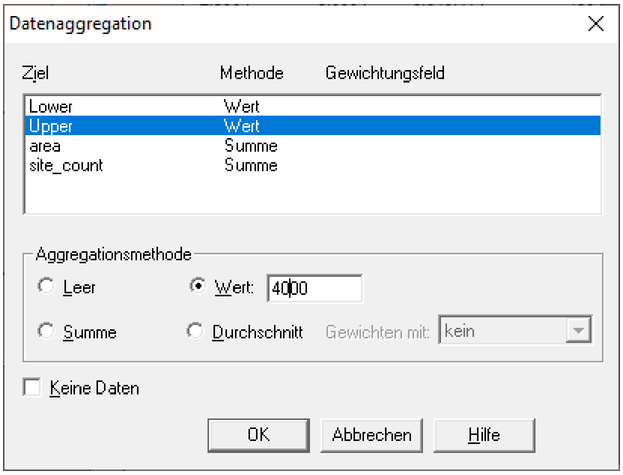

In the *Data Aggregation* dialogue, we set the *value* for *Lower* to the lowest interval limit (*here 6.649*) and the *value* for *Upper* to 4000. For *Area* the sum of the km² within the isoline shall be calculated. Press *OK* to start the process. The file must now be reloaded. The result is one object of all selected isolines. Single isoline areas cannot be addressed individually. Therefore there is only one data line in the table with the summed area (km²), all other lines are empty.

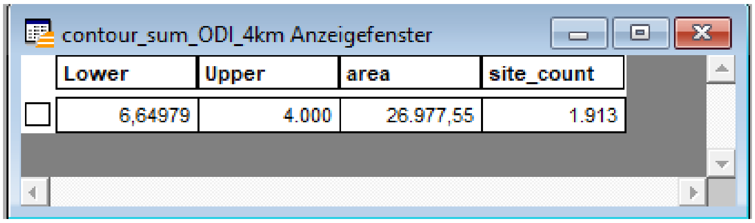

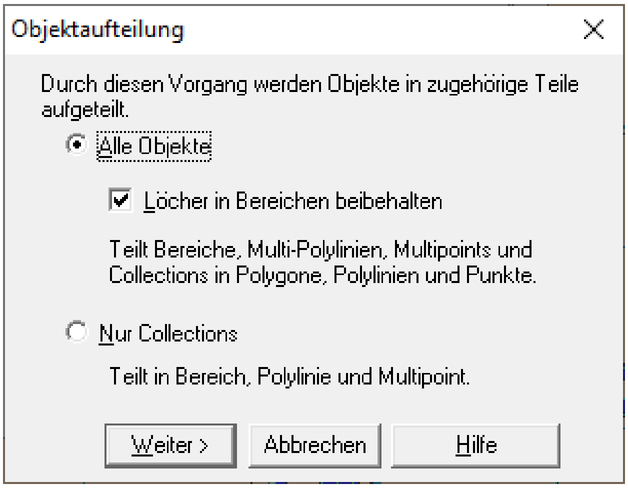

If you want to select Core Areas in the map section individually, they must again be divided into individual objects, but without internal structure. Select all Core Areas and proceed.

Open */Objects /Splitting*. In the new dialogue window the option *All objects* is selected. *Keep holes in areas*. With *Next* you return to data aggregation. For *Lower, Upper,* and *site_count: value* is entered, for *area*: *proportion*. With *Next* the individual settlement areas are separated and the proportionate areas are calculated.

Result

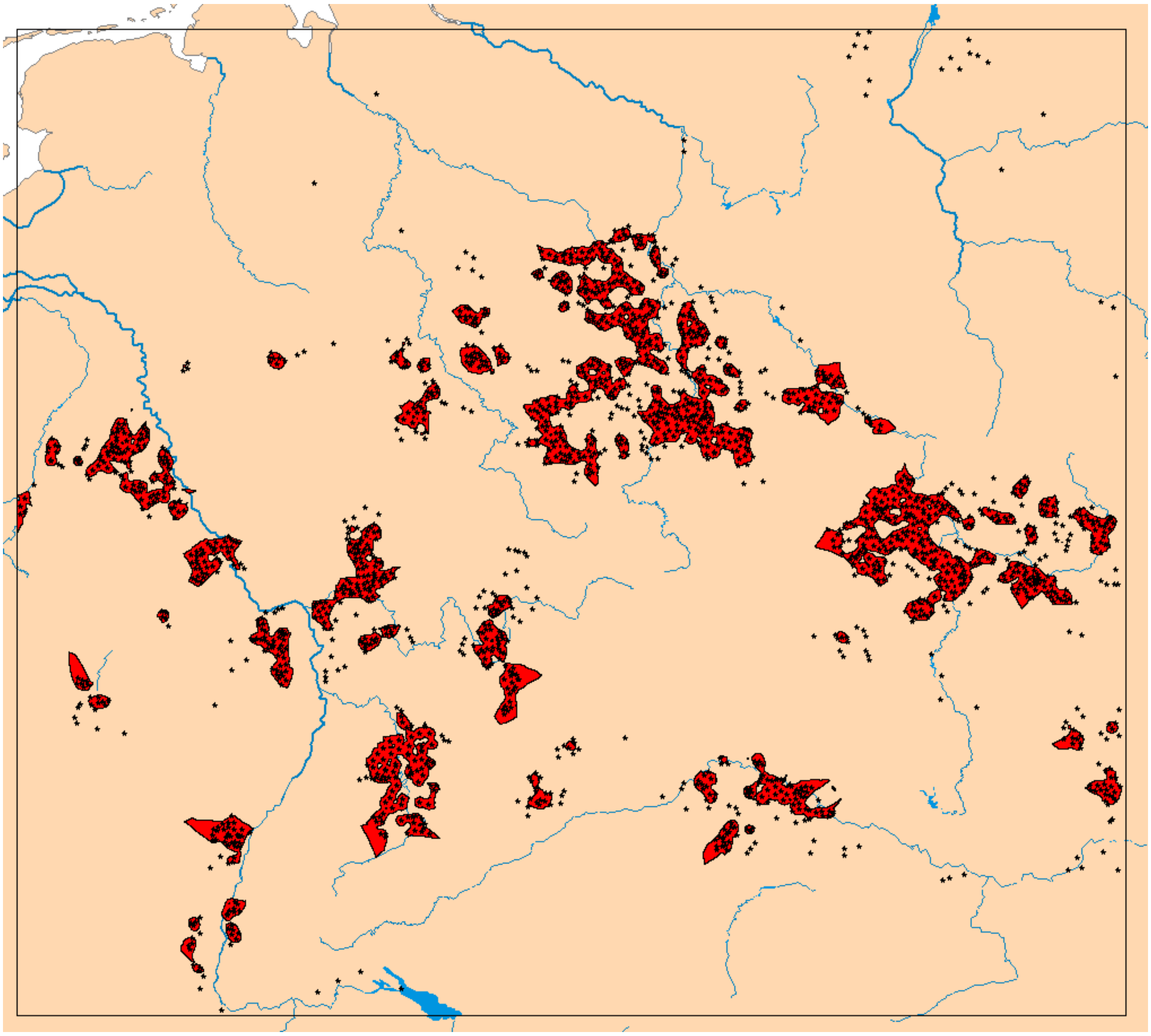

We now get a MapInfo Table with individually selectable Optimally describing Isolines, which all have the same lower limit in site density.

A final result could look like the map above.

Have fun and success trying it out

References:

Broich, M.; Peters, R. 2020: GitHub repository CologneProtocol-R: https://github.com/C-C-A-A/CologneProtocol-R.

Haas, A.G.; Viallix, J.R. 1976: Krigeage applied to geophysics. The answer to the problem of estimares and contouring. *Geophys. Prospecting* 24, 49–69.

Herzog, I. 2007: Testing GIS Methods by Means of Simulation Detecting and Describing Find Spot Densities. In: Magistrat d. Stadt Wien - MA 7, Stadtarchäologie (Hrsg.), *Archäologie und Computer 2006 : Workshop 11* (Wien 2007), Contribution on CD-Rom.

Hilpert, J.; Wendt, K.P.; Zimmermann A. 2007: Estimation of prehistoric population densities with GIS supported methods. In: Magistrat d. Stadt Wien - MA 7, Stadtarchäologie (Hrsg.), *Archäologie und Computer 2006 : Workshop 11* (Wien 2007), Contribution on CD-Rom.

Preparata, F.P.; Shamos, M.I. 1988: *Computational Geometry. An Introduction*. Springer, New York.

Preuss, J. (ed.), 1998: *Das Neolithikum in Mitteleuropa: Kulturen, Wirtschaft, Umwelt vom 6. bis 3. Jahrtausend v. u. Z., Übersichten zum Stand der Forschung*. Beier & Beran, Weissbach.

Zimmermann, A.; Richter, J.; Frank, Th.; Wendt, K.P. 2004; Landschaftsarchäologie II-Überlegungen zu Prinzipien einer Landschaftsarchäologie. *Bericht der Römisch-Germanischen Kommission* 85, 37–95.

Zimmermann, A.; Wendt, K.P.; Frank, T.; Hilpert, J. 2009. Landscape Archaeology in Central Europe. *Proceedings of the Prehistoric Society* 75, 1–53.

Schmidt, I.; Hilpert, J.; Kretschmer, I.; Peters, R.; Broich, M.; Schiesberg, S.; Vogels, O.; Wendt, K. P.; Zimmermann, A.; Maier, A. 2020: Approaching Prehistoric Demography: Proxies, Scales and Scope of the Cologne Protocol in European contexts. *Philosophical Transactions of the Royal Society B*.
