# Supplementary material for: Approaching prehistoric demography: proxies, scales and scope of the Cologne Protocol in European contexts
Source: Philos Trans R Soc Lond B Biol Sci. 2020 Nov 30;376(1816):20190714. doi: 10.1098/rstb.2019.0714 (PMC7741091; doi:10.1098/rstb.2019.0714)
Supplement: Manual S02: Manual and example application to model ‘Core-Areas’ (Optimally Describing Isolines) using ArcGIS [file rstb20190714supp3.pdf]

Supplementary file for:

Approaching Prehistoric Demography: Proxies, Scales and Scope of the Cologne Protocol in European contexts

Schmidt, Isabell; Hilpert, Johanna; Kretschmer, Inga; Peters, Robin; Broich, Manuel; Schiesberg, Sara; Vogels, Oliver; Wendt, Karl Peter; Zimmermann, Andreas; Maier, Andreas

Computing the Cologne Protocol in ArcGIS Desktop 10.X. A tutorial using an Early Neolithic data set from Central Europe

Oliver Vogels
oliver.vogels@uni-koeln.de
April 2020

Notation conventions:
Name of file, folder or directory
Colum- or row heading
/Menu / 1st submenu / 2. submenu / etc.; button
Option, value entries
(exemplification)
<default value>

Introduction

This tutorial describes the first two parts of the Cologne Protocol, GIS-analyses of site distribution and the identification of the so-called Core Areas. For a discussion of the theoretical background and the assessment of population densities please refer to the main text and the related suppl. Material. See also https://github.com/C-C-A-A/CologneProtocol-ArcGIS.

The goal of the manual is to compute the first two parts of the Cologne Protocol in ArcGIS Desktop 10.X (using the extensions Spatial Analyst and Geostatistical Analyst). The necessary steps are (after. *Schmidt et al. 2020, table S2*):

Steps:
1. Loading data (Shape-layer with sites as points)
2. Creating of Voronoi Diagram
3. Extraction of Vertices
4. Aggregation of Verticies
5. Defining the Radius of the “Largest Empty Circle”
6. Kriging - Preparation and Grid

7. Kriging - Semivariogram
8. Kriging - inspect and export raster output
9. Creating Contour Lines (Isolines)
10. Calculating the Area and Number of Sites per isoline
11. Data export
12. Selecting the “Optimally Describing Isoline”

Archaeological sites (Shapefile with sites as points)

In this tutorial, we use archaeological sites derived from map of the Early Neolithic Linear Pottery Culture (LBK) in Central Europe. The map is based on *Preuss (1998, Karte 1)* and available from the CRC 806 database (crc806db.uni-koeln.de). The original map included symbols for an agglomeration of five sites. The digital data set has been processed to resolve this issue by aggregating corresponding sites. Another prerequisite of the workflow of the Cologne Protocol is that distances can be computed in metres, kilometres etc., which is impossible in case the data set (sites) are projected in a geographic coordinate system. In the latter case it is necessary to project the date set to a projected coordinate system. Here, DHDN Gauss Krüger Zone 3 (EPSG: 31467) will be used.

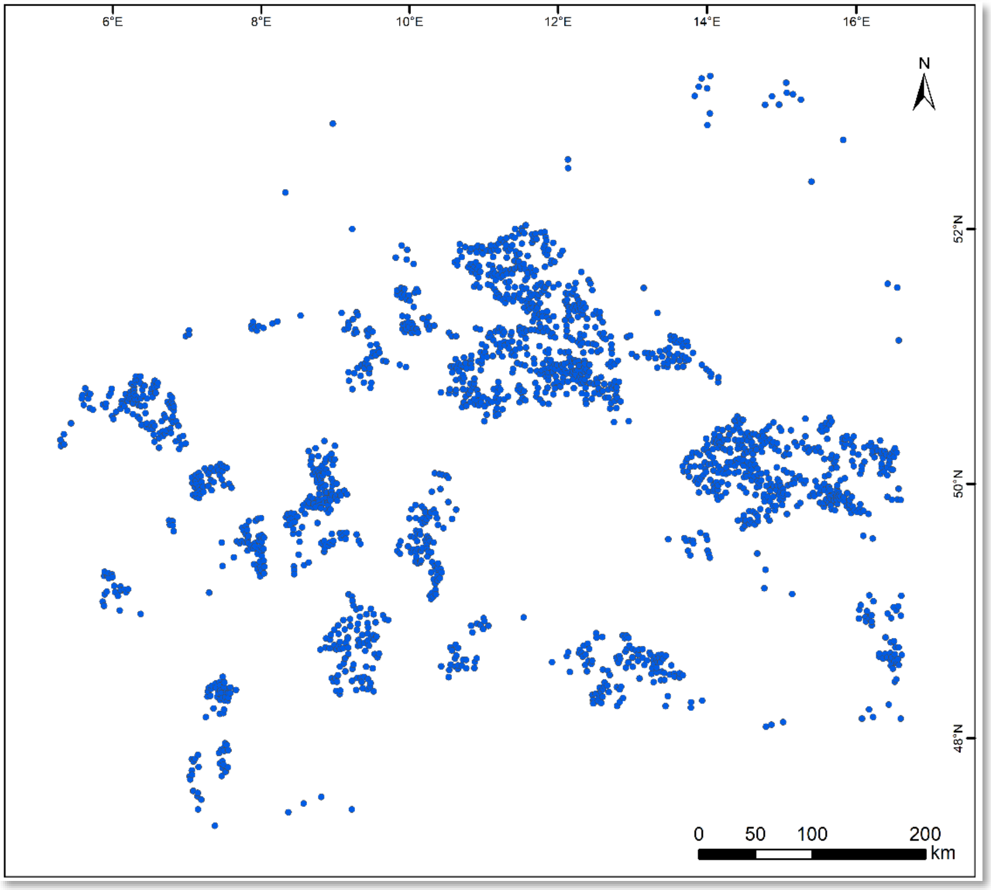

Largest Empty Circle

The “Largest Empty Circle” (LEC), or more precisely, the radius of a LEC is a measure of site distance. Every LEC has its centre at a vertex of a Thiessen polygon (e.g. Voronoi diagram) and exactly three sites are located on every circumference of a LEC. An illustration can be found at *Zimmermann et al. (2004, fig. 5)*. In conclusion, areas with larger site distances will be characterized by larger radii of LECs and, logically, areas with smaller site distances will be characterized by smaller radii of LECs.

Step 2: Creating Thiessen (Voronoi) Polygons

In the first computational step Thiessen-Polygons will be created (select the file, here Sites_GK3) using the tool *Analysis /Create Thiessen Polygons*. Define output directory and file name. Choose either all or only the Field ID in *Output Fields*. Press OK to proceed:

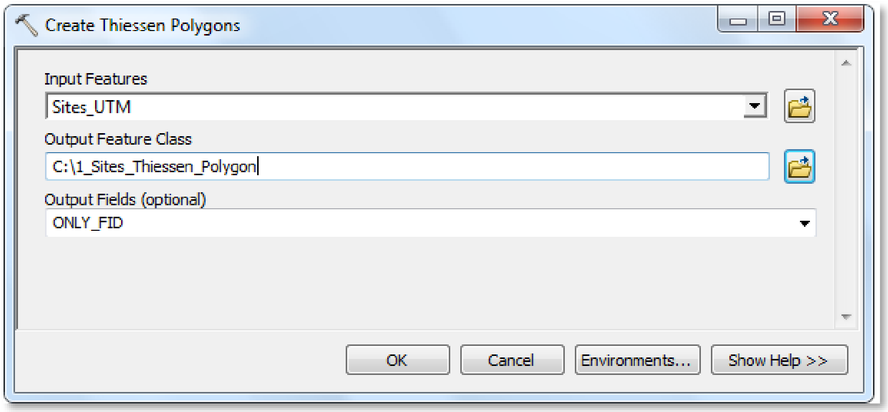

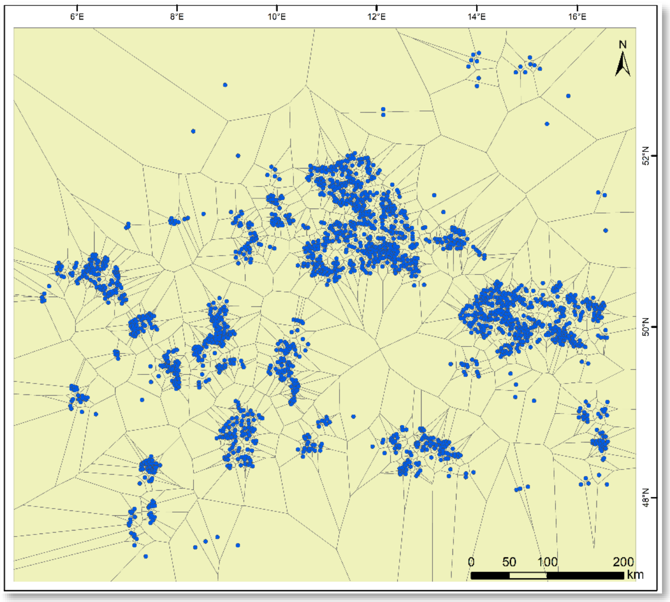

Step 3: Extraction of TP-node points (vertices)

To extract the nodes of the Thiessen-Polygons („vertices“) from the Thiessen-Polygon-Layer (here 01_Thiessen-Polygons) use */Data Management /Feature Vertices to Points*. In Dropdown *Point Type* choose *All*.

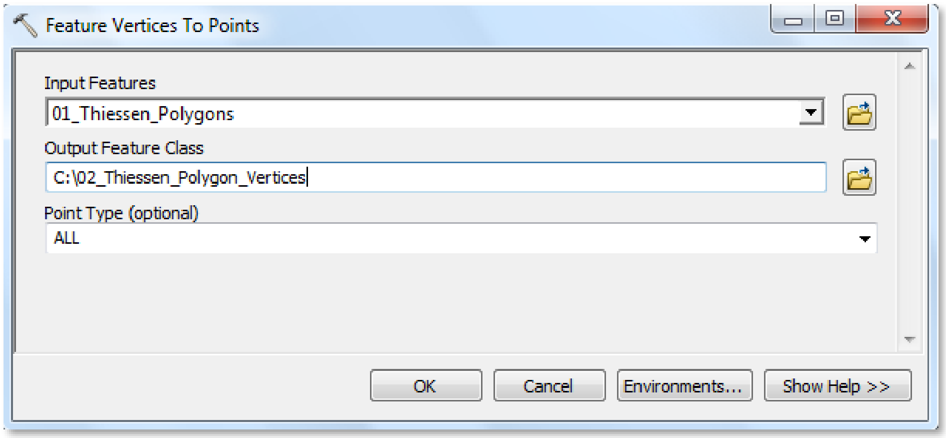

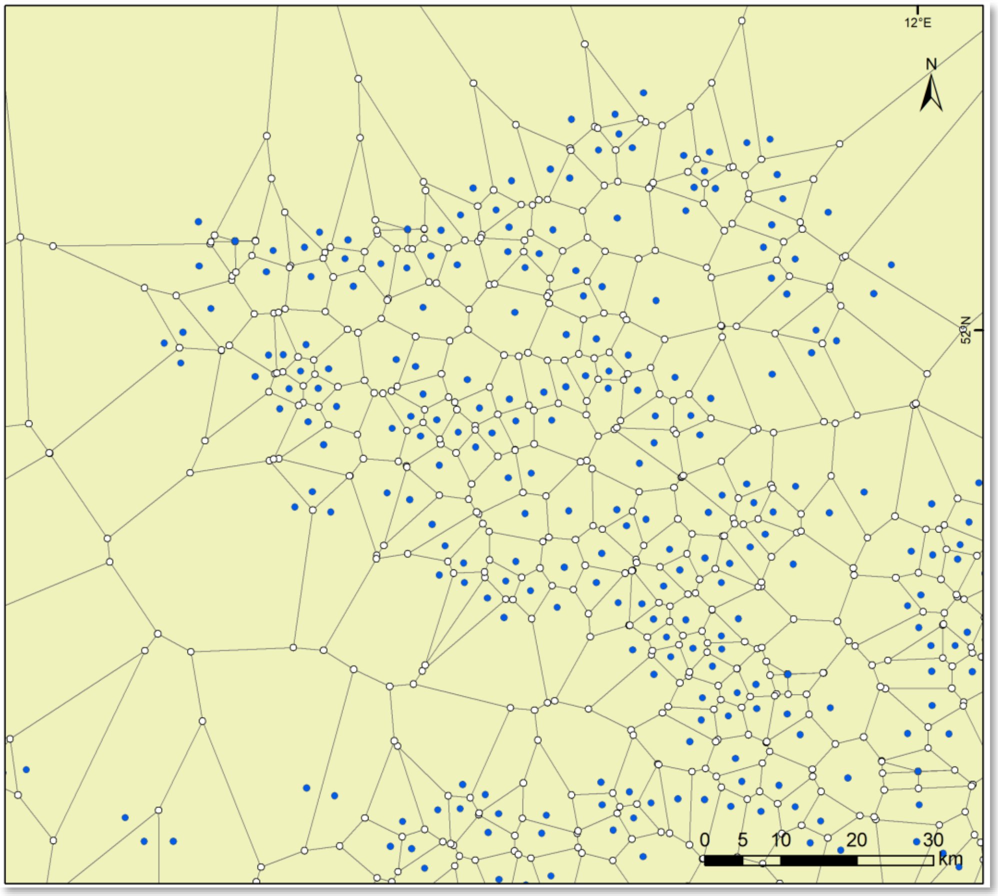

Step 4: Aggregation of vertices

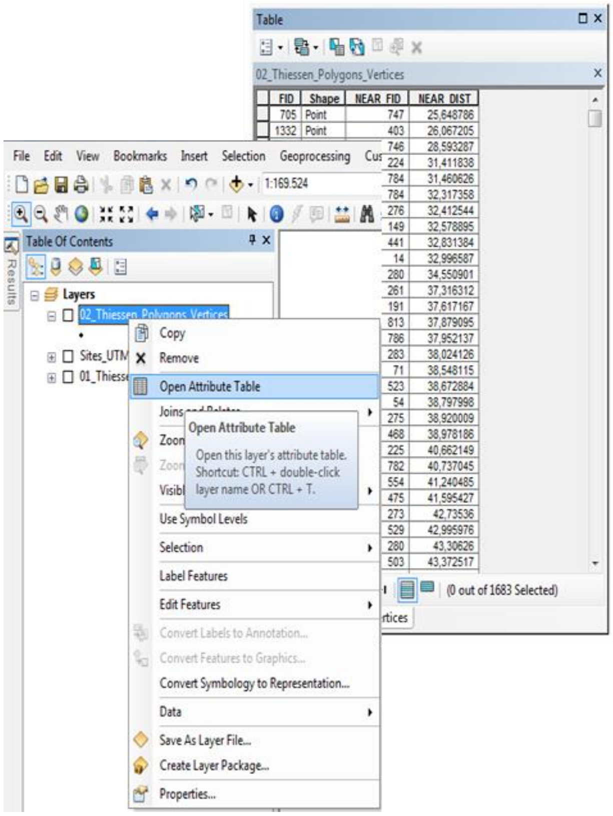

During step 3 we didn't take care of duplicate vertices. Furthermore, some of the vertices are located at the outer border of the working areas, so-called border points. These points create artificial site distances and it must be decided on a case-by-case basis whether the border points should be deleted. Here only the duplicates will be removed, not the border points.

This is accomplished by deleting vertex doublets with identical coordinates. First, coordinates need to be extracted. This is done using *<u>/Data Management /Add Geometry Attributes</u>*. Select the file 02_Thiessen-Polygon_Vertices as Input Feature and chose *<u>Point X Y Z M</u>* at *<u>Geometry-Properties</u>*. Since no further calculations are required, other fields can remain empty.

A look at the attribute table (right-click on the vertex layer -> open attribute table in the table of contents) shows whether two columns with the values *POINT X* and *POINT Y* have been entered in the table and whether this also contains data (not selected on screenshot).

Now we proceed with Layer 02_Thiessen-Polygons_Vertices by using the tool *<u>/Data Management /Delete Identical</u>* to delete double vertices. In the tool window check marks at "Point X" and "Point Y" are to be selected. Press OK to proceed.

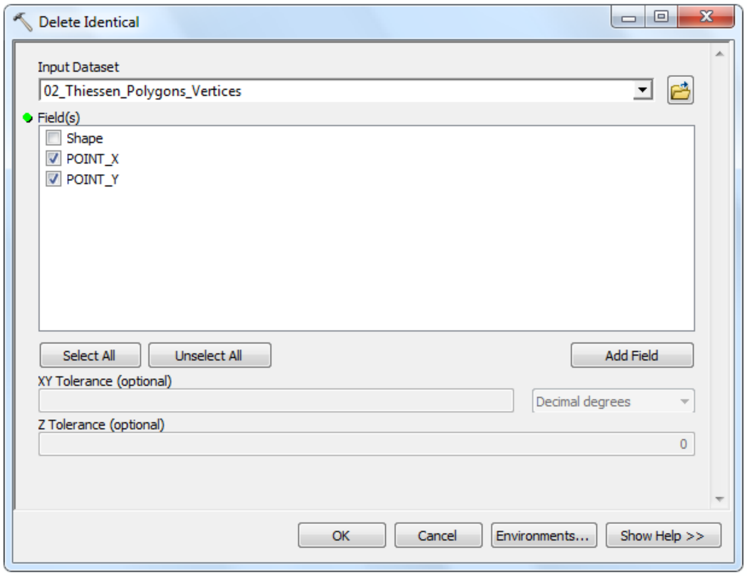

Step 5: Defining the radius of the "Largest Empty Circle"

Each of the vertices is by definition the centre point of a circle that passes through the three most proximate sites. Since the distance to each of the three sites is identical, the distance can be measured as the nearest neighbour, or as the radius of the „Largest Empty Circle" (LEC).

Now the LEC radius using can be determined using the tool *Analysis Near*. Select the aggregated layer of Vertices as "Input Feature" (e.g. 02_Thiessen-Polygons-Vertices) and the original layer of archaeological sites (Sites_GK3) as *Near Feature*. Search radius may remain empty, as *Method* we recommend *Planar*.

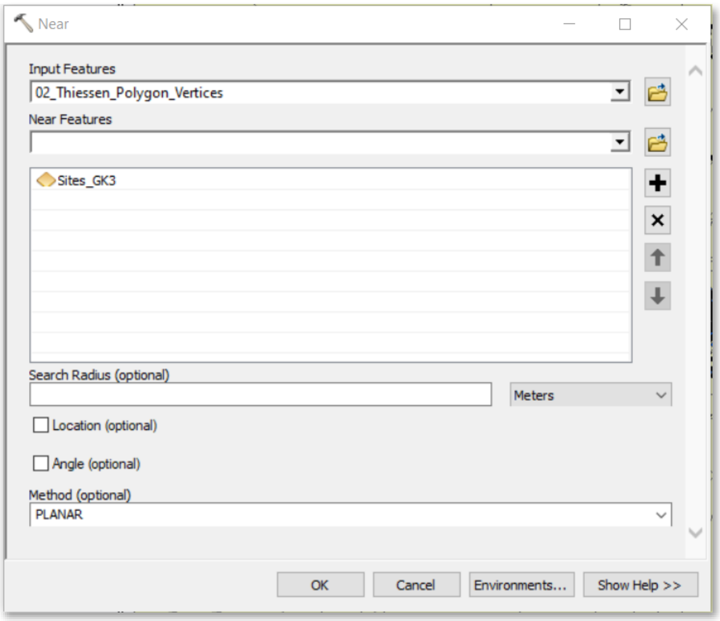

A look into the layer table of 02_Thiessen-Polygons-Vertices reveals two new colums: *Near FID* contains the ID of the next nearest site (from table Sites_GK3). *Near Dist* contains the distance to the next site (given in the distance measure defined in the data frame properties, usually metres). i.e. the radius of the LEC.

Excursion: The largest empty circles can be produced with the tool *Analysis Buffer*: using the file 02_Thiessen-Polygons-Vertices as *Input Features*, and the Field *Near_Dist* as *Buffer Distance.*

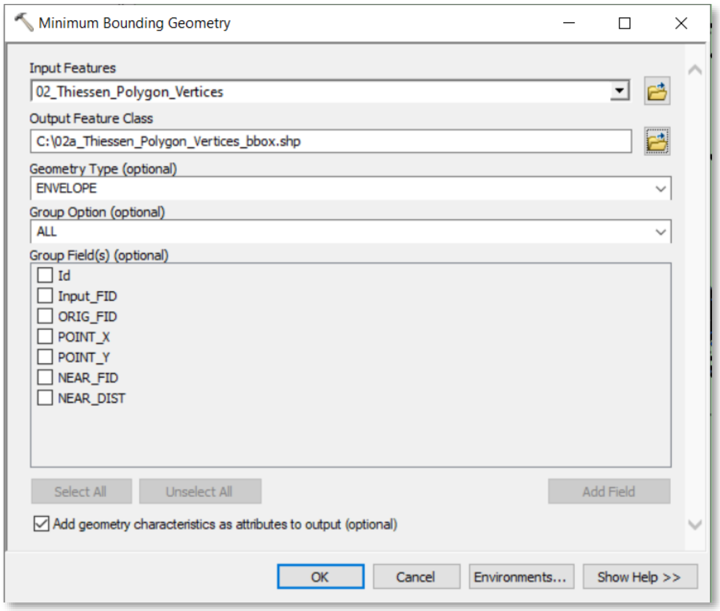

Kriging

Step 6: Preparation

In the next step, clustering of sites will be interpolated using kriging. The method has a variety of parameters which need to be estimated to fit the data (SuppMat Tab. S2). One of the parameters that needs to be given is the distance between each pair of locations, the so-called "lag-distance". Usually, ArcGIS estimates the lag-distance from the size of a given grid, i.e. a raster

file or map. But since our initial input layer is a vector layer of site locations it does not contain a grid size. Following the Cologne Protocol, lag distance is derived following a standard procedure in MapInfo, which is the diagonal of the bounding box of the data set divided by 250. A bounding box is a rectangular that includes any point feature in a given layer. First, the width and length of the layer 02_Thiessen-Polygons-Vertices must be computed using the tool *<u>/Data Management /Minimum Bounding Geometry</u>*.

In a second step, a new column must be created in the attribute table of the new layer (e.g. 02a_Thiessen_Polygon_bbox). Open the attribute table (right-mouse click on the layer in the table of contents -> *<u>Open Attribute Table</u>*). In the attribute table, open the menu in the top left corner and choose *<u>Add Field</u>*. Choose a name (e.g. *bbox_diag*) and choose *<u>Double</u>* as *<u>Type</u>*. Hit *OK* to create the new column.

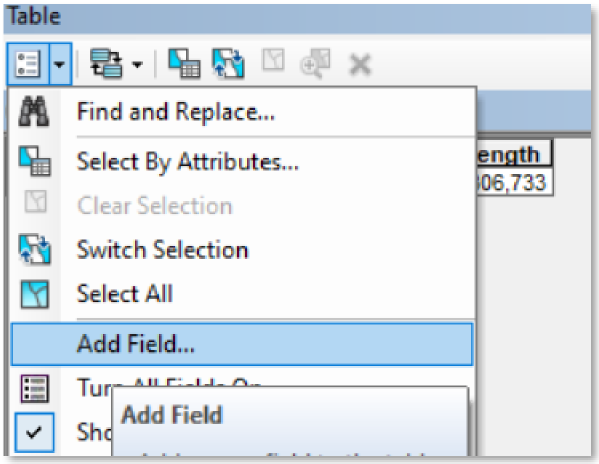

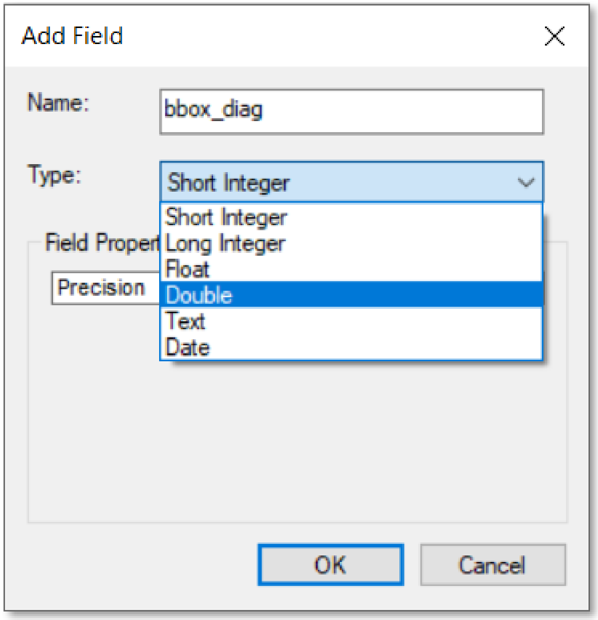

When the new column *bbox_diag* has been created hit the column header with a right-mouse click and choose *<u>Field Calculator</u>*. At the top, choose *<u>VB Script as Parser</u>*. The following line of code calculates the diagonal of the bounding box from its width and length:

```
Sqr ( [MBG_Width]^2 + [MBG_Length]^2 )
/ 250
```

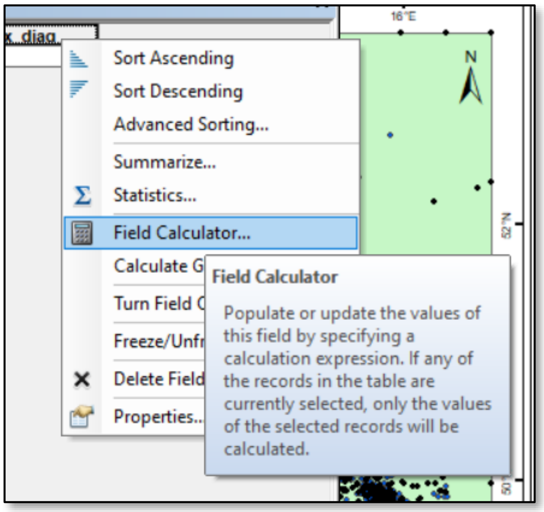

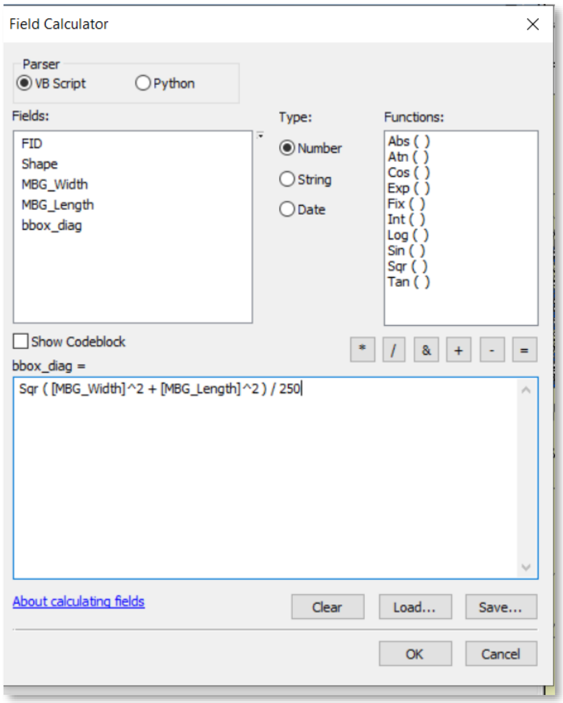

Here, the resulting lag distance (bounding box diagonal /250) is “4376.84” metres (ca. 4.3 km). In case the value is given in kilometres it might be worth checking the current main layer properties (right mouse button click on the top layer in the table of contents -> Properties). On the tab *General* map units can be verified.

Step 7: Semivariogram

Once the distance of the diagonal of the bounding box has been defined, kriging can be computed. In ArcGIS several Kriging tools exist: in case all statistical parameters (nugget, sill, major range, lag distance, search distance etc.) are known the tool */Spatial Analyst /Kriging* can be used. Recalculating the parameters automatically can be done by the tool /Geostatistical Analyst / Moving Window Kriging. If none of the values is known, the kriging model can be fitted automatically, using the */Geostatistical Analyst /Empirical Bayesian Kriging*. Finally, a semivariogram can be fitted manually, using the *Geostatistical Wizard*. Following the procedure of the Cologne Protocol, we will fit the model manually. Fortunately, in ArcMap the semivariogram is applied via a wizard that guides through several consecutive steps. The wizard can be started from the *Geostatistical Analyst* toolbar (*Customize -> Toolbars -> Geostatistical Analyst*). At the toolbar, the *Geostatistical Wizard* can be started either via a button or from a dropdown (a very interesting feature in ArcGIS is that any parameter can be changed even once the final interpolation map has been computed).

- In the first window, choose *Kriging/CoKriging* in the left pane, make sure that 02_Thiessen-Polygons_Vertices is selected as the *Source Dataset* in the right pane and *NEAR_DIST* (the LEC radii) in the dropdown *Data Field*.
- In the second window, choose *Ordinary* as the *Kriging Type* and *Prediction* as the *Output Surface Type* in the left pane and *none* as *Transformation Type* as well as *Order of trend removal* in the right pane.
- The third window is made of three parts, a graphical representation of the covariance (model fit) at top left, a covariance map at bottom left. Here, points that are closer together are less distant than points that are further apart. The semivariogram allows you to explore this distribution in your data set. The process of fitting a semivariogram model to capture the spatial relationships in the data is known as variography. The model can be changed by editing parameters in the right pane. Four values need to be edited here. First, change the model type to *Spherical*. Second, set *Calculate nugget* to *False* and enter a *Nugget* of “0” manually. Now it is worth optimizing the fit by hitting the button next to *Optimize model*. Finally, *Lag Size* should be set to the diagonal of the bounding box divided by 250 (here: 1094210.62681 / 250 = 4376.84; cf. step 6; This method for estimating the lag distance is derived from MapInfo). Finally, the *Number of Lags* (pairs of points taken into account) may be set between 20 and 40.

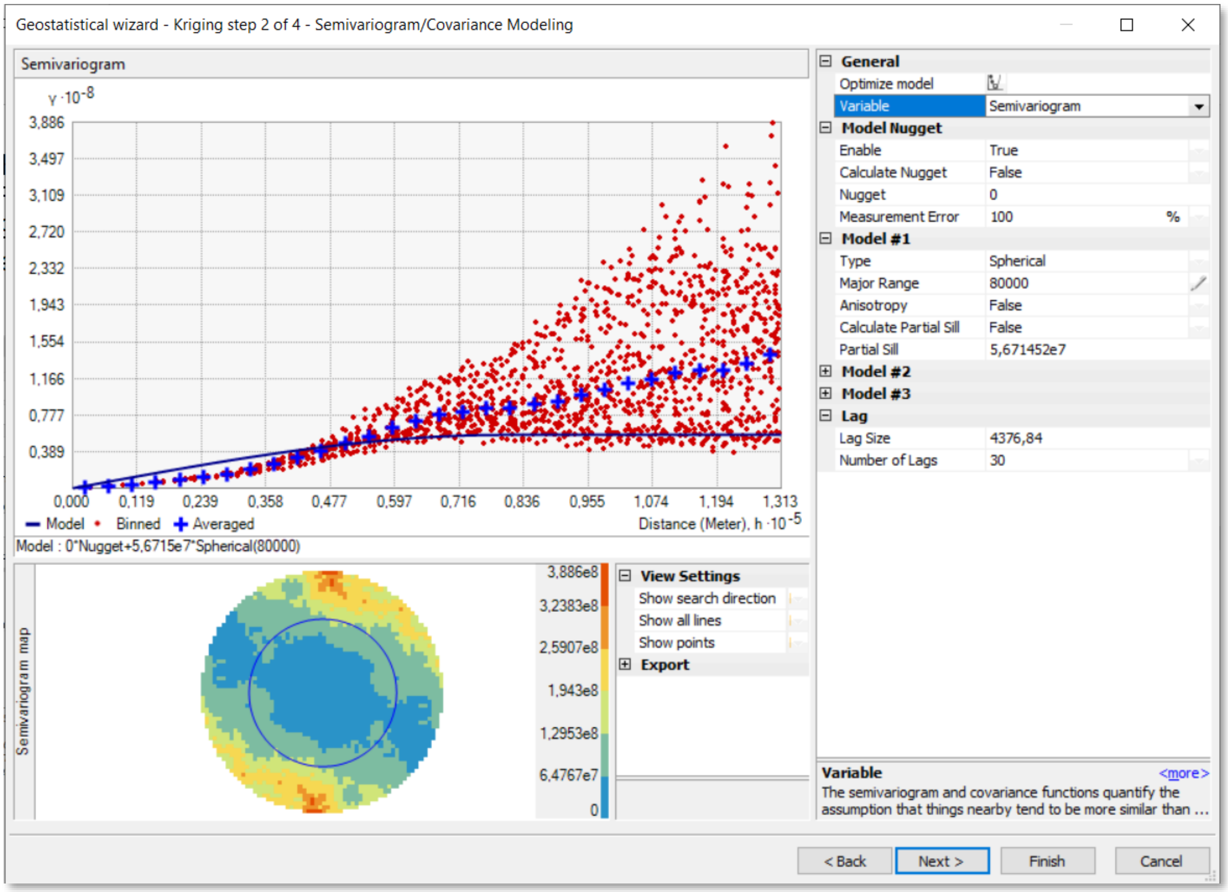

Remark: According to the Cologne Protocol the *Major range*, should be set to the first peak of the distribution of the variance. This, however, is not possible in ArcGIS since the map in the top left pane does not display variance (but either covariance or lag pairs). But the same effect can be modelled by setting the *Major Range* to a value at which the fit (black line) follows the lower bottom of the distribution. Here this effect is reached if *Major Range* is set to ca. 8000.

– In the next window, a preview of the Kriging model is given in the top left pane. In the right pane, two values need to be edited: *Maximum neighbours* should be set to "10"; *Minimum neighbours* should be set to "3".

– After pressing *Next*, a list of values is given with measurements, predictions and errors for the individual sites.

Press *Finish* to proceed.

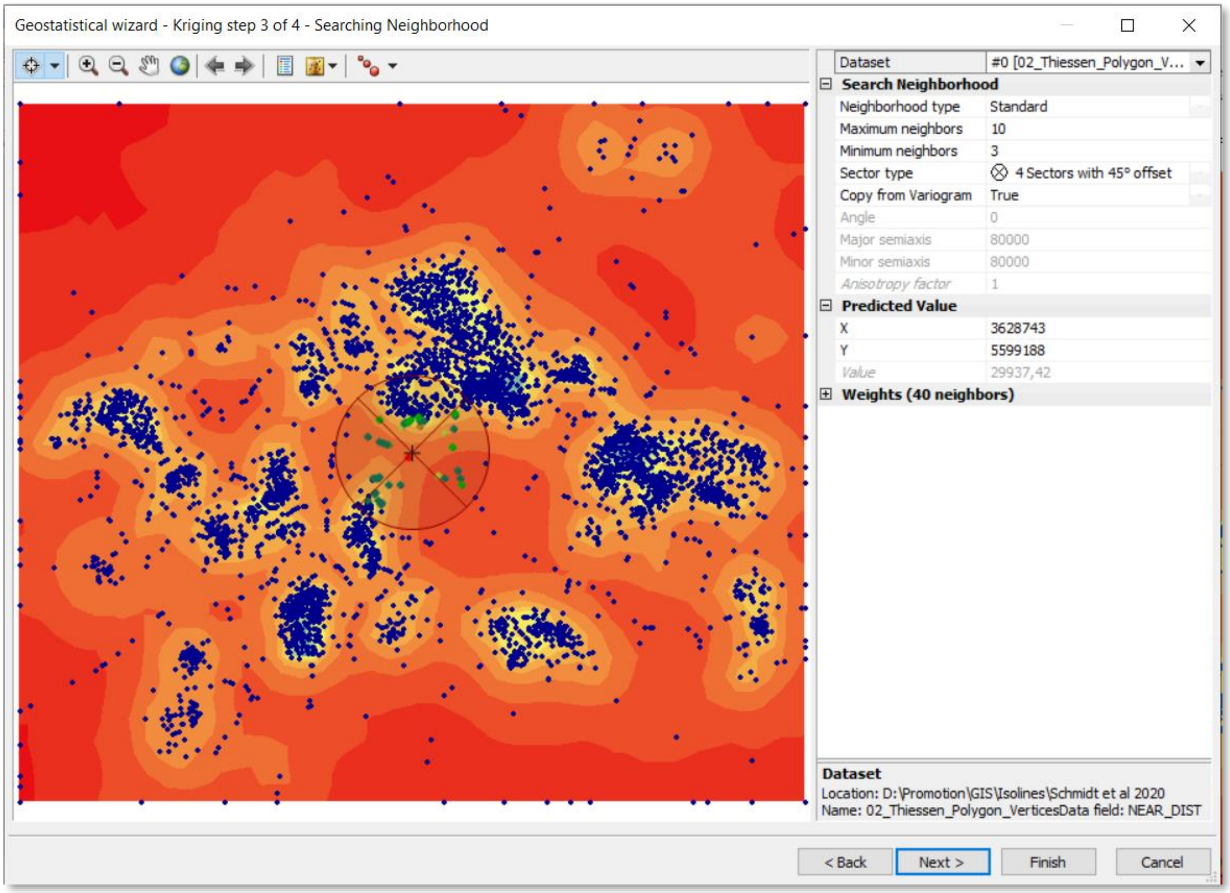

Step 8: Kriging - inspect and export raster output

It is worth taking a closer look at the GA Layer's options. Via a right mouse button click on the resulting Kriging map there are too more interesting options available in the dropdown menu. *Change Output to Prediction Standard Error* switches the map view (when done an option to *Change Output to Prediction* becomes available in the same place). The other interesting option is the second last entry in the dropdown menu: *Method Properties*. This entry enters the *Geostatistical Wizard* again and allows to modify the Kriging parameters.

The resulting Kriging interpolation map is a temporary "GA Layer" that should be saved as a grid file for further use. This can be done in two ways, either by using the tool */Geostatistical Analyst / GA Layer To Grid*, or via a right mouse button click on the Kriging interpolation map -> Data -> Export To Raster. While exporting, an *Output Cell Size* should be chosen that fits the envisaged distance of contour lines in the next step, i.e. if the resulting isolines should be in intervals of 500 m, choose cell size smaller than 500 m. In the following steps we assume the Kriging map is saved as a layer named kriging.tif (ArcGIS does not allow numbers at the beginning of filenames when exporting raster files).

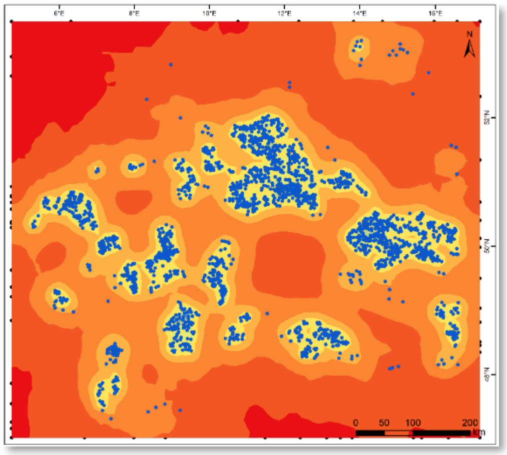

The Prediction map obtained by Kriging using the LEC radius values

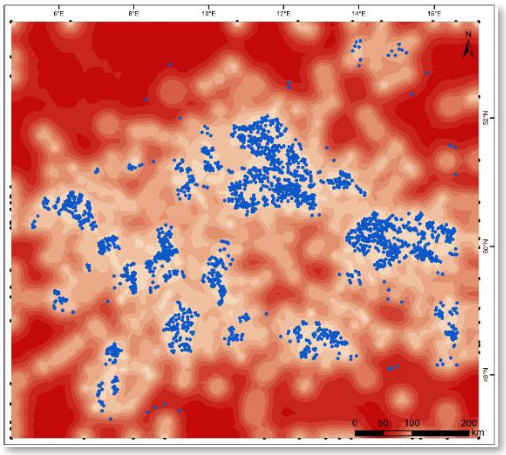

The same map switched to Standard Error view.

Optimally Describing Isolines

On the basis of the kriging results we will create isolines and we will select the “Optimally Describing Isoline” (ODI). The selection of the ODI is based upon several statistical parameters of the isolines (*Zimmermann et al. 2004, 53–55*).

Step 9: Creating Contour Lines (Isolines)

Isolines will be created between 500 m and 20 km with an interval of 500m using the tool *[/Spatial Analyst /Contour]*. The starting distance of 500 m is chosen to neglect clusters at smaller distances (0–500m). In the subsequent step 10 we will identify some characteristics of the different isolines, such as the volume of the area or the number of sites included. This task requires the isolines to be polygons. Another issue concerns the way polygons are created by the *Contour* tool. In ArcGIS 10.6 and above, the *Contour Type: CONTOUR_POLYGON* creates neighbouring polygons: 0 – 500; 500 – 1000 etc. But we need to calculate the increase in area volume and archaeological sites between the separate isolines, and hence overlapping polygons: 0 – 500; 0 – 1000 etc. This can be accomplished using *Contour Type: CONTOUR SHELL.*

9.a ArcGIS Desktop 10.6 and above

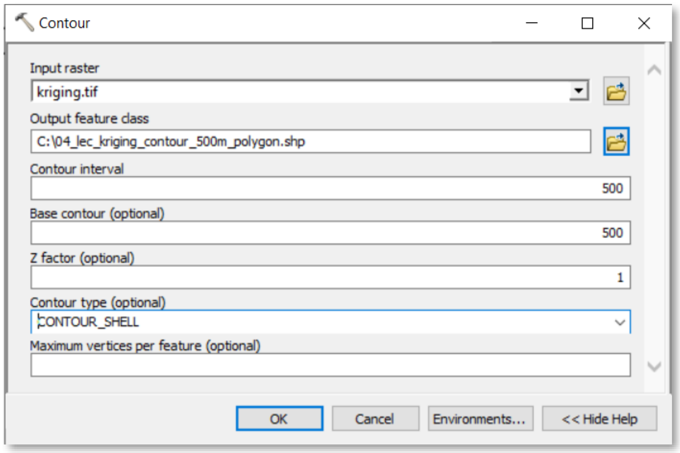

– *Input Raster* kriging.tif from the previous step 8.

– *Output feature class*: Use the browser to name and store the file (here 04_lec_kriging_contour_polygon_500 m). Note: specify the meter/km of the

interval, in case you calculate several sets.

– *Contour interval* to be chosen dependent on size of the study area and distances between sites. Here: 500 m

– *Base-Contour*, i.e. the starting contour line, we also choose *500* m.

– *Contour Type*: *CONTOUR SHELL*

9.b ArcGIS Desktop 10.5 and below

In case you are using ArcGIS 10.5 or earlier, the tool is only capable of producing polylines:

– *Input Raster:* kriging.tif from previous step 8.

– *Output feature class*: Use the browser to name and store the file (here 04_lec_kriging_contour_polyline_500m). Note: specify the meter/km of the interval, in case you calculate several sets.

– *Contour interval* to be chosen dependent on size of the study area and distances between sites. Here: *500* m.

– *Base-Contour*, i.e. the starting contour line, here *0*.

– *Contour Type*: *CONTOUR*

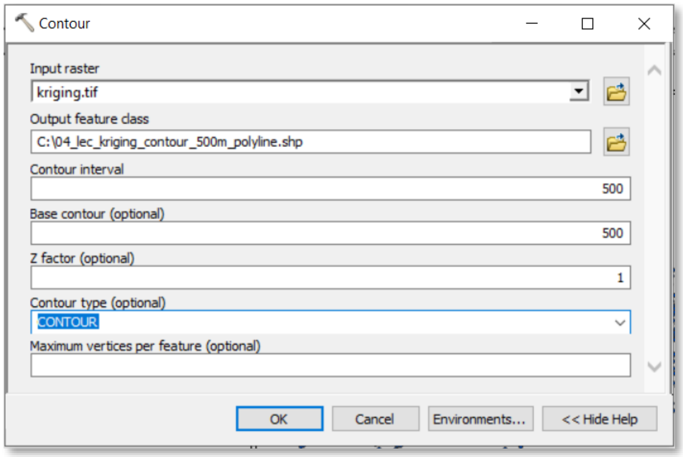

Since we need to calculate the area volume per contour line (isoline), the individual contour line features need to be transformed into polygons. Well, transforming polylines to polygons is a simple task with the tool */Data Management /Feature To Polygon*. However, for some reason the latter tool deletes all data columns in the resulting data set, even if the option *Preserve attributes (optional)* is ticked on. For this reason, the transformation has to be done for each contour line separately! To do this, we recommend to select single values in the Layer Table (e.g. select all rows where CONTOUR = 500, e.g. using the *Select by attributes* option in the attribute table and the formula *"Contour" = 500*) at once and to . After each export, it is recommended to open the attribute table of the resulting layer and add a new column named *Contour* with the value of the currently exported Contour value (e.g. *500*). Since this procedure may become highly time-consuming when the number of contour lines is high (as it is in this example), we recommend to automate this process using the Modelbuilder:

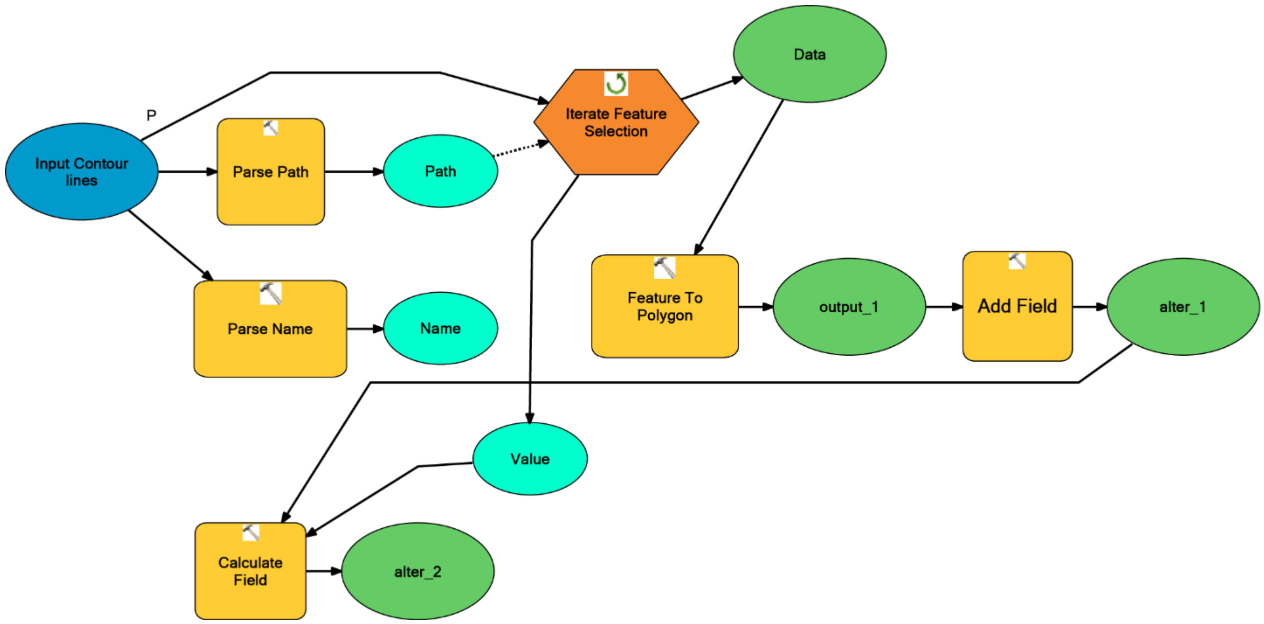

This process can be downloaded as an ArcGIS toolbox at Github. After running the script, one polygon feature class exists per contour value (500, 1000, 1500 etc.). Regardless whether you do this step manually or using the model builder, at the end you should have a series of shapefiles named 04_lec_kriging_contour_polygon_XXXXm.

All these files now must be merged to a single shapefile using the tool */Data Management /Merge*. Using the folder icon next to the *Input Datasets* dropdown allows you to choose all the contour line files at once. Pick an output file name (we assume 04a_lec_kriging_contour_polygon_merged) and start the process. Now you can delete the series of 04_lec_kriging_contour_polygon_XXXXm files (In case you used the Modelbuilder toolbox these files are also marked with "temp" in their filename).

If you check the resulting the layer 04a_lec_kriging_contour_polygon_merged you will realize that it still contains a series of rows per isoline value. But for the envisaged calculations in the next step, we need one row per isoline. Hence, we use */Data Management /Dissolve* to collapse identical isoline values into multipart features:

- *Input feature:* 04a_lec_kriging_contour_500m_polygon_merged
- *Output Feautre Class:* e.g. 04b_lec_kriging_contour_500m_polygon_dissolved
- *Dissolve Fields: Contour*

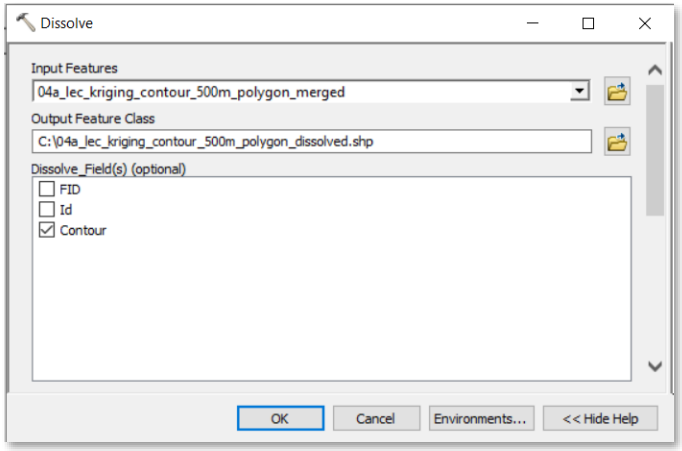

Step 10: Calculating the Area and Number of Sites per Isoline

n order to be able to select the optimally describing isoline (ODI), it makes sense to look at the statistical properties of the isolines. These are, for example, the number of archaeological sites within the isolines, their difference in number per interval, the number of distinct areas per isoline or the area increase per interval. Here, we will focus on two of these characteristics: the number of archaeological sites per isoline interval and the increase in area per isoline interval.

10 a counting sites per isoline

The number of sites per isoline can be computed via a spatial join of LEC polygons from step 9. Two ways exist, both making a spatial join between the polygons and the archaeological sites, either following this workflow, or using the tool */Analysis /Spatial Join*:

- *Target-Feature*: 04_lec_kriging_contour_polyline_500m (ArcGIS 10.6 and above) 04b_lec_kriging_contour_500m_polygon_dissolved (ArcGIS 10.5 and below)
- *Join-Feature*: Sites_GK3.
- *Output Feature Class*: 05_lec_kriging_contour_500m_polygon_site_counts.
- *Join operation*: *Join One_To_One*.
- Untick *Keep all target features* to delete polygons which do not contain any sites.
- *Field Map of Join Features*: Keep the columns *Contour*, and, if available, individual site names (here *_ID*) delete others to avoid nonsense data in the resulting data set.
- Check that the *Merge Rule* for your *Contour* / *ContourMax* s set to *First*, and for the columns with individual site names (here *_ID*) is set to *Count*.
- *Match Option*: *Contains*
- Other fields are left empty.

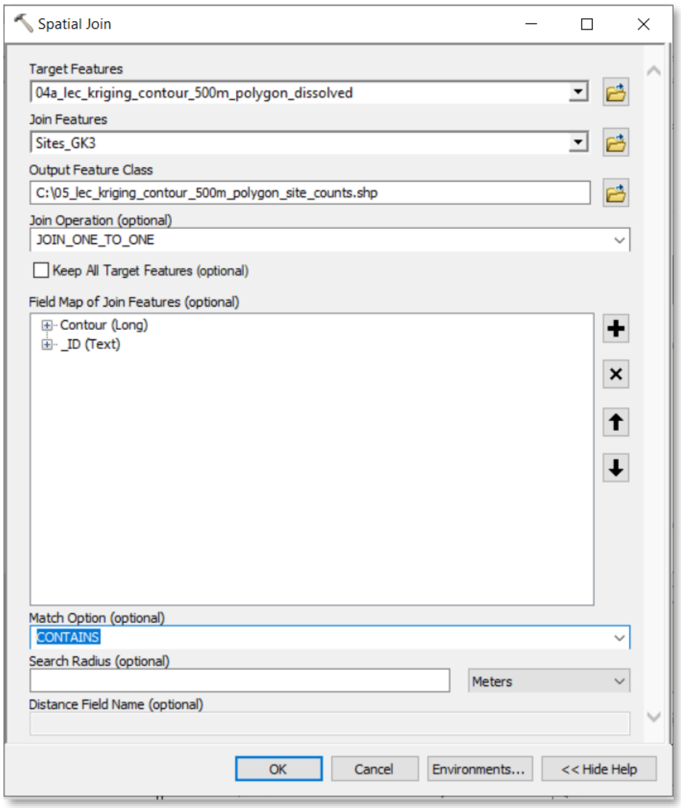

Hit *OK* to run the tool.

10 b area volume per isoline

Counting the area volume is a comparably easy task that is accomplished using again the tool /Data Management /Add Geometry Attributes:

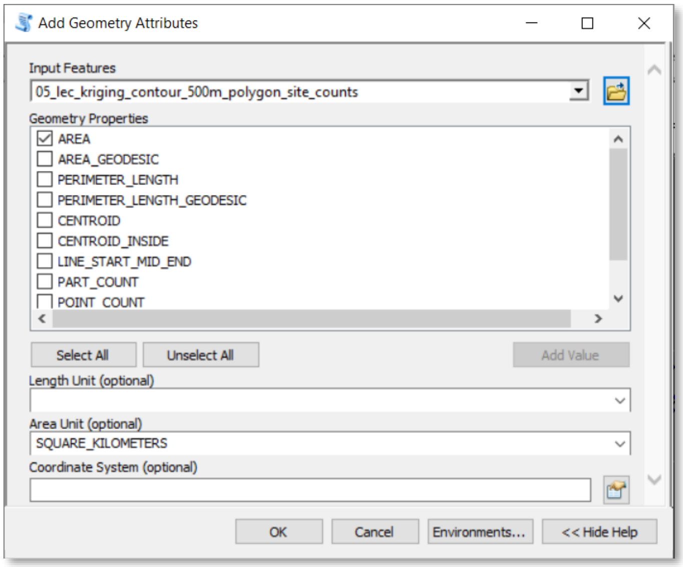

- *Input Feature*: 05_lec_kriging_contour_500m_polygon_site_counts.
- Since we have selected an area feature, we now can select under *Geometry Properties*: *Area*.
- Leave *Length Unit* may remain empty.
- *Area Unit* should contain a measure, either square meter or square kilometre.

After running the tool, area volume should be present in the Attribute table of 05_lec_kriging_contour_500m_polygon_site_counts:

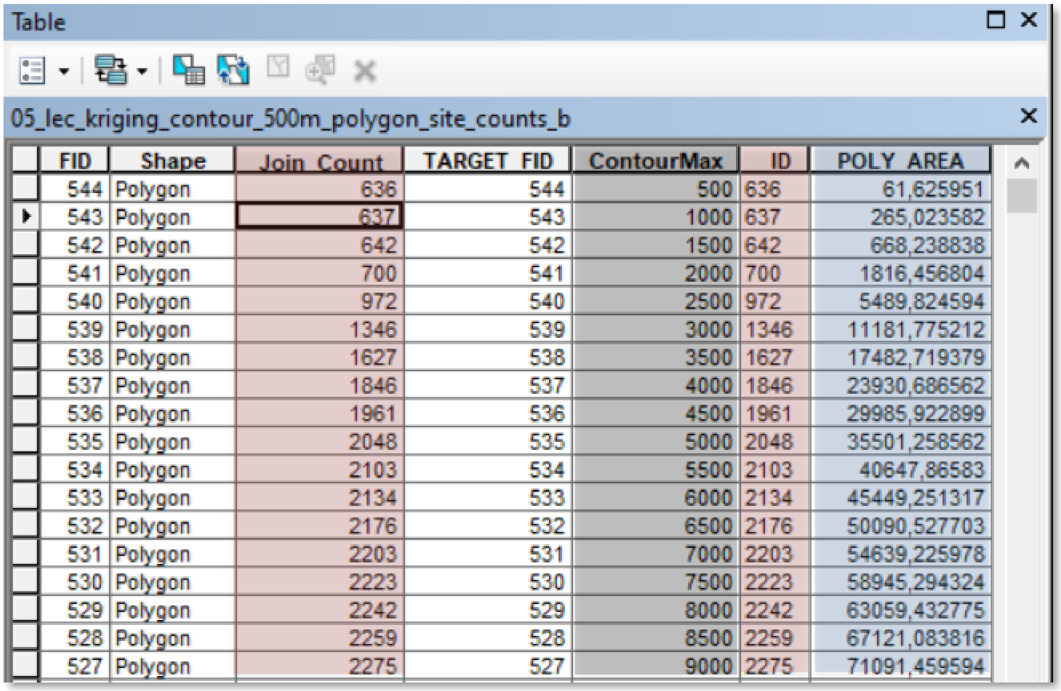
Table

05_lec_kriging_contour_500m_polygon_site_counts_b

FID	Shape	Join_Count	TARGET_FID	ContourMax	ID	POLY_AREA
544	Polygon	636	544	500	636	61,625951
543	Polygon	637	543	1000	637	265,023582
542	Polygon	642	542	1500	642	668,238838
541	Polygon	700	541	2000	700	1816,456804
540	Polygon	972	540	2500	972	5489,824594
539	Polygon	1346	539	3000	1346	11181,775212
538	Polygon	1627	538	3500	1627	17482,719379
537	Polygon	1846	537	4000	1846	23930,686562
536	Polygon	1961	536	4500	1961	29985,922899
535	Polygon	2048	535	5000	2048	35501,258562
534	Polygon	2103	534	5500	2103	40647,86583
533	Polygon	2134	533	6000	2134	45449,251317
532	Polygon	2176	532	6500	2176	50090,527703
531	Polygon	2203	531	7000	2203	54639,225978
530	Polygon	2223	530	7500	2223	58945,294324
529	Polygon	2242	529	8000	2242	63059,432775
528	Polygon	2259	528	8500	2259	67121,083816
527	Polygon	2275	527	9000	2275	71091,459594

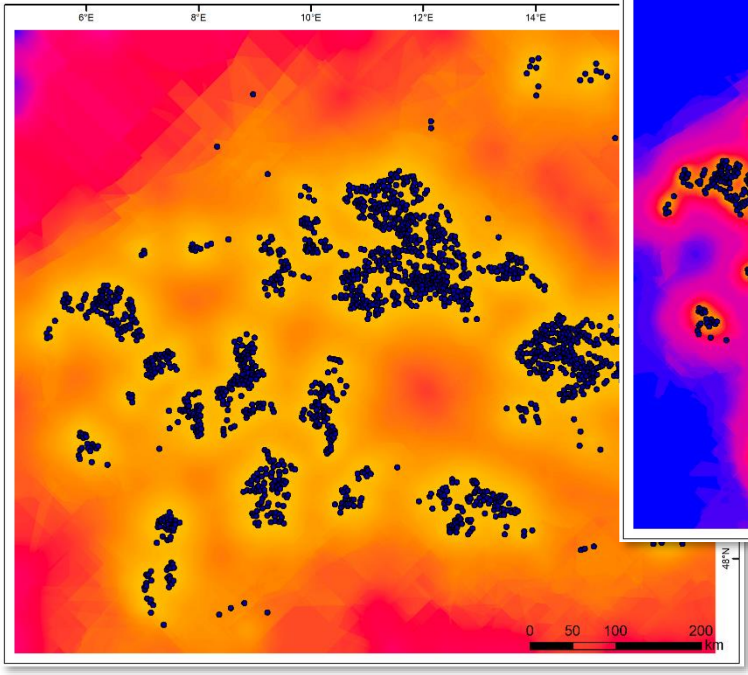

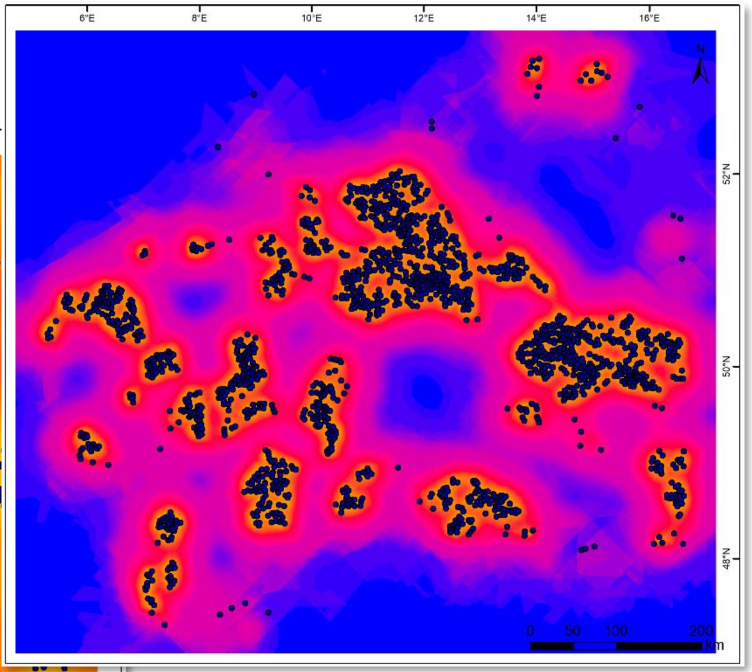

Same map, polygons coloured by the number of containing sites

Resulting polygons coloured by contour values

Step 11: Data export

The final table can be exported e.g. to an Excel spreadsheet using the tool *<u>/Conversion /Table to Excel.</u>*

Step 12: Selecting the Optimally Determining Isoline

Please refer to the workflow provided in the MapInfo manual. It is worth noting that the ODI should not be selected automatically based on a maximum value of included space because distinct archaeological cultures may display two maxima which need an

archaeological interpretation (e.g. patterns of fission and fusion in hunter/gatherer societies, cf. Kretschmer et al. 2016). Moreover, in some cases it is not possible to observe a maximum and instead with increasing distance between sites a continuous increase of space is to be observed. This would indicate a data set indicating a poor archaeological record (*cf. Zimmermann et al. 2009, 10*).

Bibliography

Hengel, T., **2007**. A Practical Guide to Geostatistical Mapping of Environmental Variables. (Luxembourg 2007).

Kretschmer, I., Maier, A., Schmidt, I., **2016**. Probleme und mögliche Lösungen bei der Schätzung von Bevölkerungsdichten im Paläolithikum. In: Ker, T., K. Nowak, G. Roth (Eds.), Alles was zählt. Feschrift für Andreas Zimmermann.

Preuss, J. (ed.), **1998**. Das Neolithikum in Mitteleuropa: Kulturen, Wirtschaft, Umwelt vom 6. bis 3. Jahrtausend v. u. Z., Übersichten zum Stand der Forschung (Weissbach 1998).

Schmidt, I., Hilpert, J., Kretschmer, I., Peters, R., Broich, M., Schiesberg, S., Vogels, O., Wendt, K. P., Zimmermann, A., Maier, A., **2020**. Approaching Prehistoric Demography: Proxies, Scales and Scope of the Cologne Protocol in European contexts. *Philosophical Transactions B*.

Zimmermann, A., Richter, J., Frank, T., Wendt, K.P., **2004**. Landschaftsarchäologie II. Überlegungen zu Prinzipien einer Landschaftsarchäologie. *Bericht der Römisch-Germanischen Kommission 85, 2004, 37-96*.

Zimmermann, A., Wendt, K.P., Frank, T., Hilpert, J., **2009**. Landscape Archaeology in Central Europe. *Proceedings of the Prehistoric Society 75, 2009, 1-53*.
