# Supplementary material for: Approaching prehistoric demography: proxies, scales and scope of the Cologne Protocol in European contexts
Source: Philos Trans R Soc Lond B Biol Sci. 2020 Nov 30;376(1816):20190714. doi: 10.1098/rstb.2019.0714 (PMC7741091; doi:10.1098/rstb.2019.0714)
Supplement: Manual S03: Manual and example application to model ‘Core-Areas’ (Optimally Describing Isolines) using QGIS/SAGA [file rstb20190714supp4.pdf]

Supplementary file for:

Approaching Prehistoric Demography: Proxies, Scales and Scope of the Cologne Protocol in European contexts

Schmidt, Isabell; Hilpert, Johanna; Kretschmer, Inga; Peters, Robin; Broich, Manuel; Schiesberg, Sara; Vogels, Oliver; Wendt, Karl Peter; Zimmermann, Andreas; Maier, Andreas

Manual and example application to model 'Core-Areas' (Optimally Describing Isolines) using QGIS/SAGA

Robin Peters
14. Mai 2020

Notation conventions:

Name of file, folder or directory
Column- or row heading
/Menu / 1st submenu / 2. submenu / etc.; button
Option, value entries
(exemplification)
<default value/>

Programs / Program Version:
- QGIS 3.10
- SAGA 2.3.1

This manual presents an example application of the **Cologne Protocol** using QGIS and SAGA. You can either execute the task stepwise by hand (see below) or use the semi-automatic QGIS Python/R scripts (*.py/*.rsx) available at the GitHub Repository (https://github.com/C-C-A-A/CologneProtocol-QGIS). The scripts are however experimental and manual and scripts come without any kind of guarantee, so please make sure to double-check your results. Regardless of what approach you choose, please read the manual and consult the publication and supplement (*Schmidt et al. 2020*).

The manual describes the first two parts of the Cologne Protocol, which are a GIS-analysis of site distribution and the identification of so-called Core Areas. The construction of Voronoi diagrams and "Largest Empty Circles" are conducted in QGIS. Kriging and converting the kriging results into isolines is done in SAGA. The

step of selecting the Optimally Describing Isoline is not explained in this manual and can be found in the R or the MapInfo manual. The aim of this manual is neither to explain the theoretical background nor the further steps of the Cologne Protocol. For these points please refer to the associated publication including the supplementary information.

Steps:

1. Shape-Layer with sites as points....................2
2. Creating Voronoi polygons....................5
3. Extraction of vertices....................6
4. Aggregation of vertices....................7
5. Defining the radius of the „Largest Empty Circle“....................9
6. Kriging – Preparations and Grid....................15
7. Kriging - Semivariogram....................18
8. Kriging - inspect and export raster output....................20
9. Creating contour lines (isolines)....................21
10. Calculating the area and the number of sites per isoline....................24
11. Data export....................27
12. Selecting the "Optimally Describing Isoline"....................28

1. Shape-Layer with sites as points

As case study we use part of a distribution map of Early Neolithic sites in Central Europe. The map is based on *Preuss (1998, Karte 1)* and available from the CRC 806 database (https://crc806db.uni-koeln.de/start/). Search for “Early Neolithic sites in Central Europe”. Besides point symbols representing single sites the original map also included symbols for an agglomeration of five sites. The digital data set has been processed to resolve this issue.
Make sure to download the files in Gauss-Krüger projection (13_earlyNeolithic_CE_sites_GK3.shp) not the WGS84 (Latitude/Longitude) files.

Start QGIS and load your or the example data set:

Layer / Add Layer / Add Vector Layer...

13_earlyNeolithic_CE_sites_GK3.shp

(formerly – and for the following screenshots:
13_earlyNeolithic_FRG_sites_GK3.shp)

Our example data set consists of 2378 early Neolithic sites.

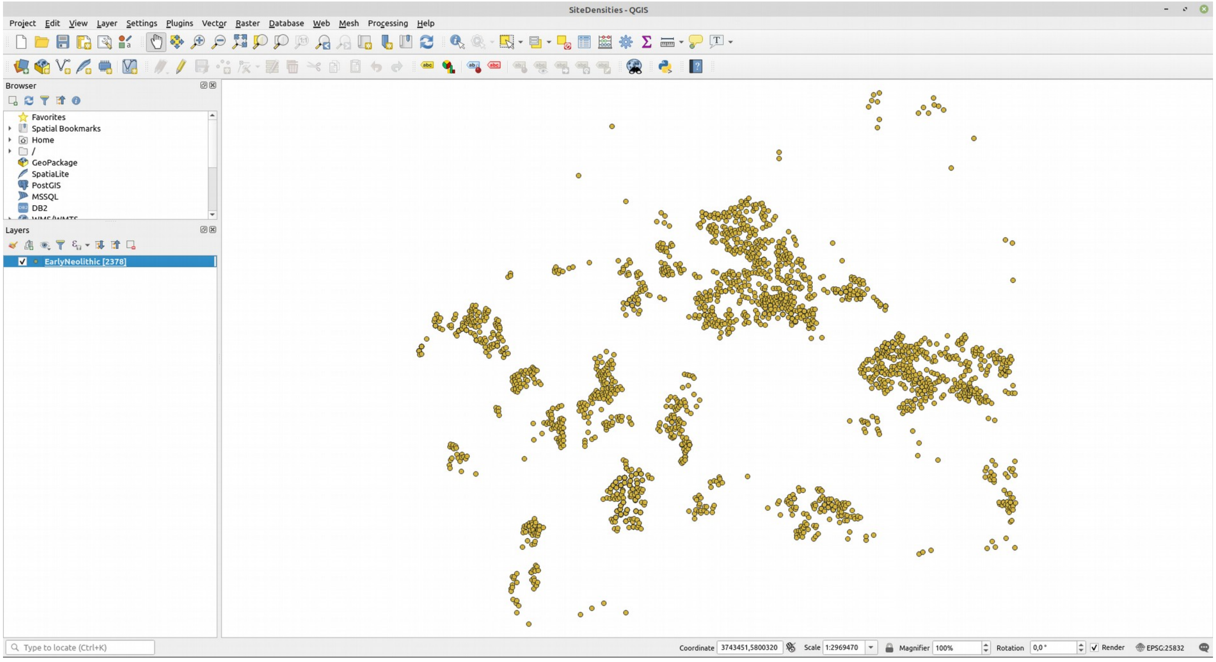

Check the projection system of your QGIS project and of the sites layer. Both should be EPSG 31467 (DHDN / 3-degree Gauss-Krüger zone 3). If they are not, change them accordingly.

Project / Properties / CRS…

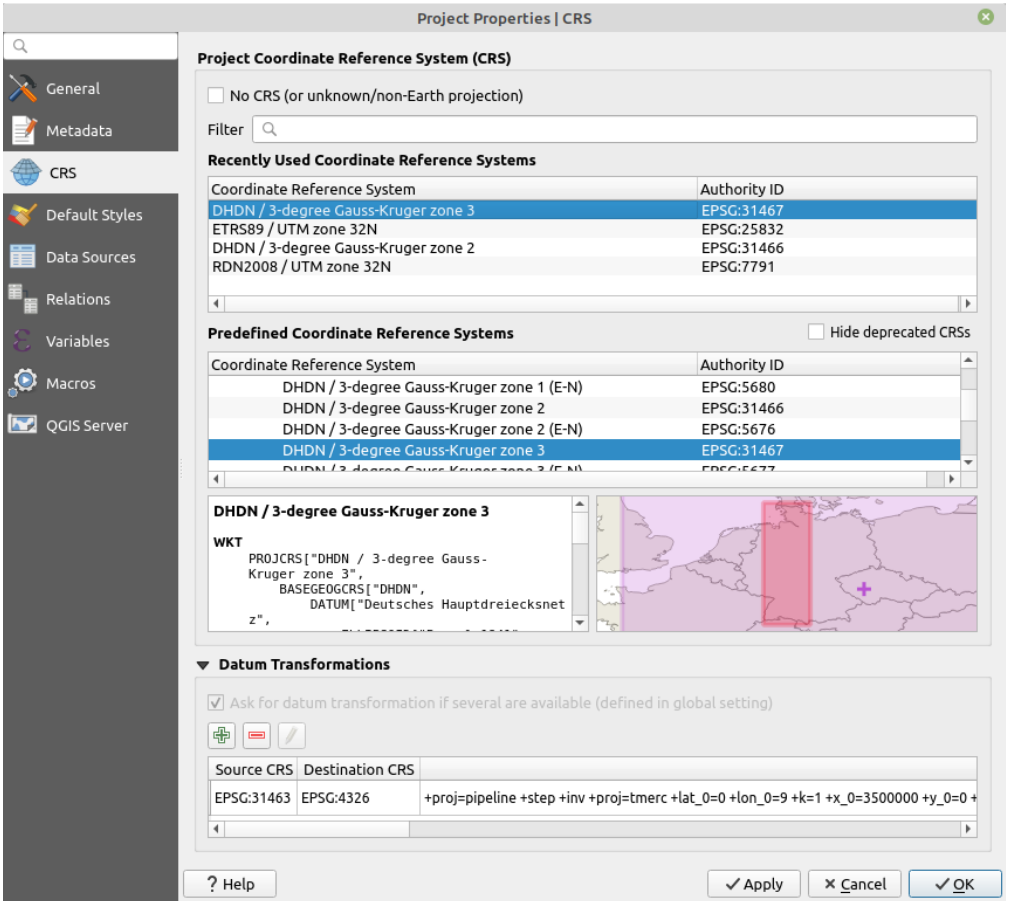

Right Click on Layer / Properties / Source...

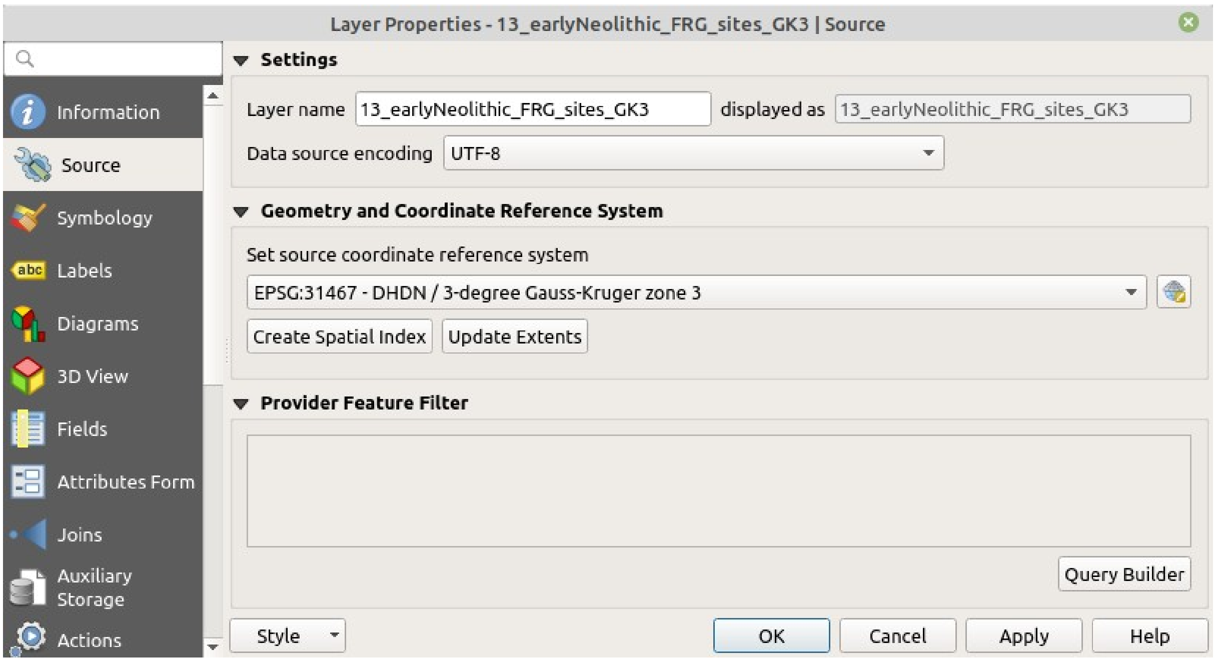

2. Creating Voronoi polygons

The Cologne Protocol uses the Largest Empty Circle (LEC) or more precisely the LEC radii to measure site distances. To locate the central points of the largest empty circles, voronoi diagrams are constructed and the voronoi vertices are extracted.

Vector / Geometry Tools / Voronoi Polygons…

<u>Input Layer:</u> 13_earlyNeolithic_FRG_sites_GK3.shp

<u>Buffer region (% of extent):</u> 0

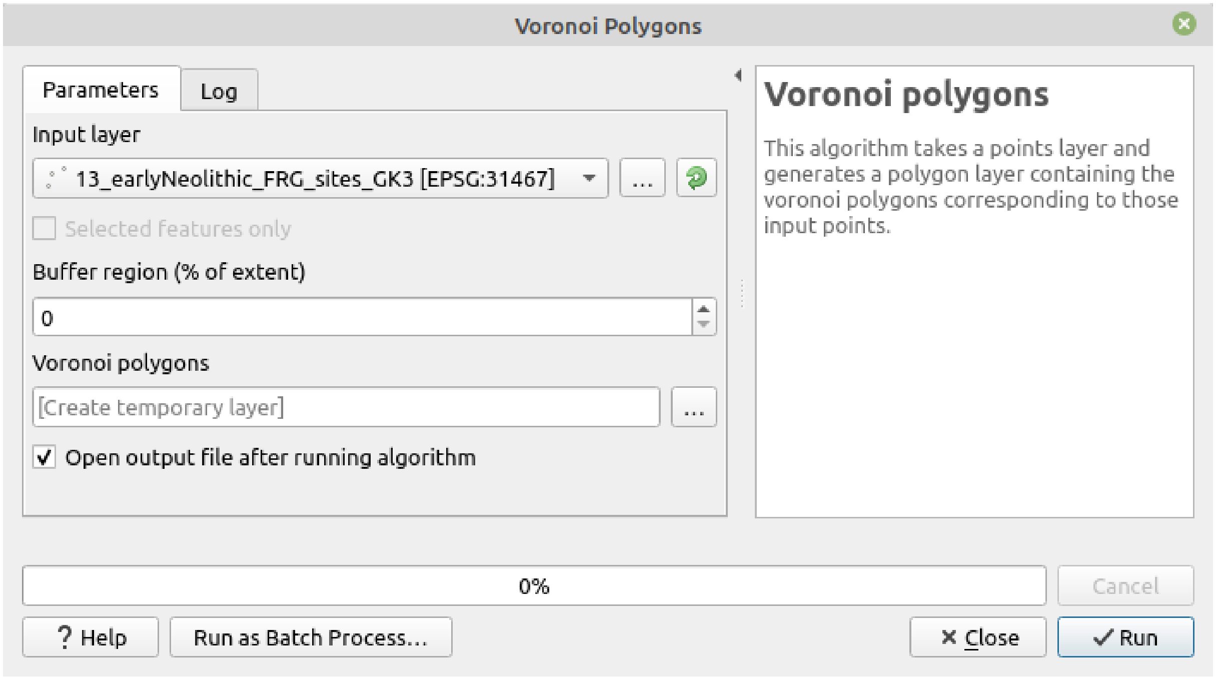

Result: Voronoi polygons.shp

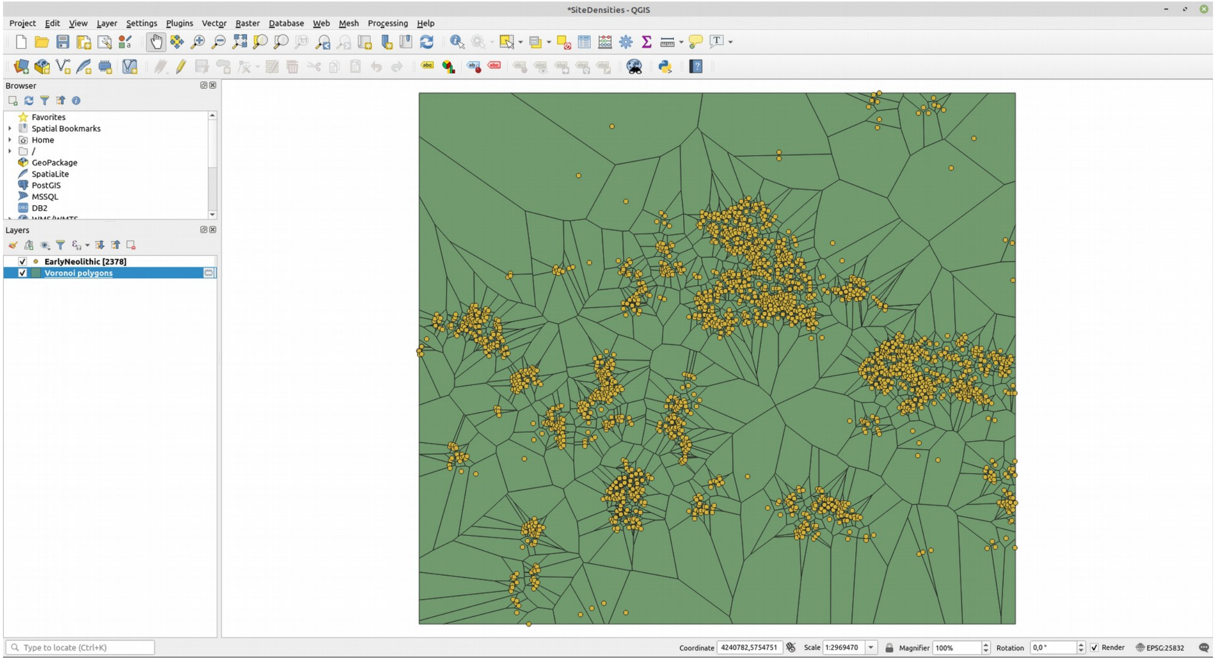

3. Extraction of vertices

Extract nodes/vertices of the voronoi diagrams.

Vector / Geometry Tools / Extract Vertices

<u>Input layer:</u> Voronoi polygons

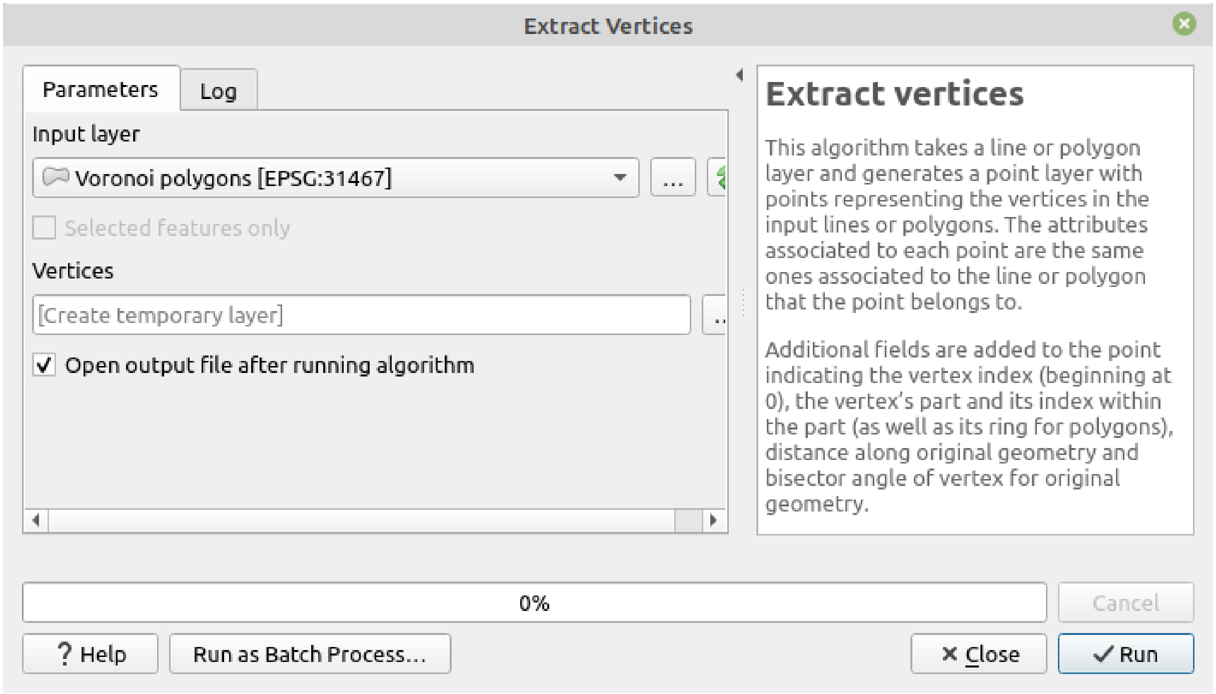

Result: Vertices.shp

Close-up with sites as red dots, voronoi diagrams as black lines and nodes/vertices as white dots. Exactly three sites are located on every circumference of a LEC.

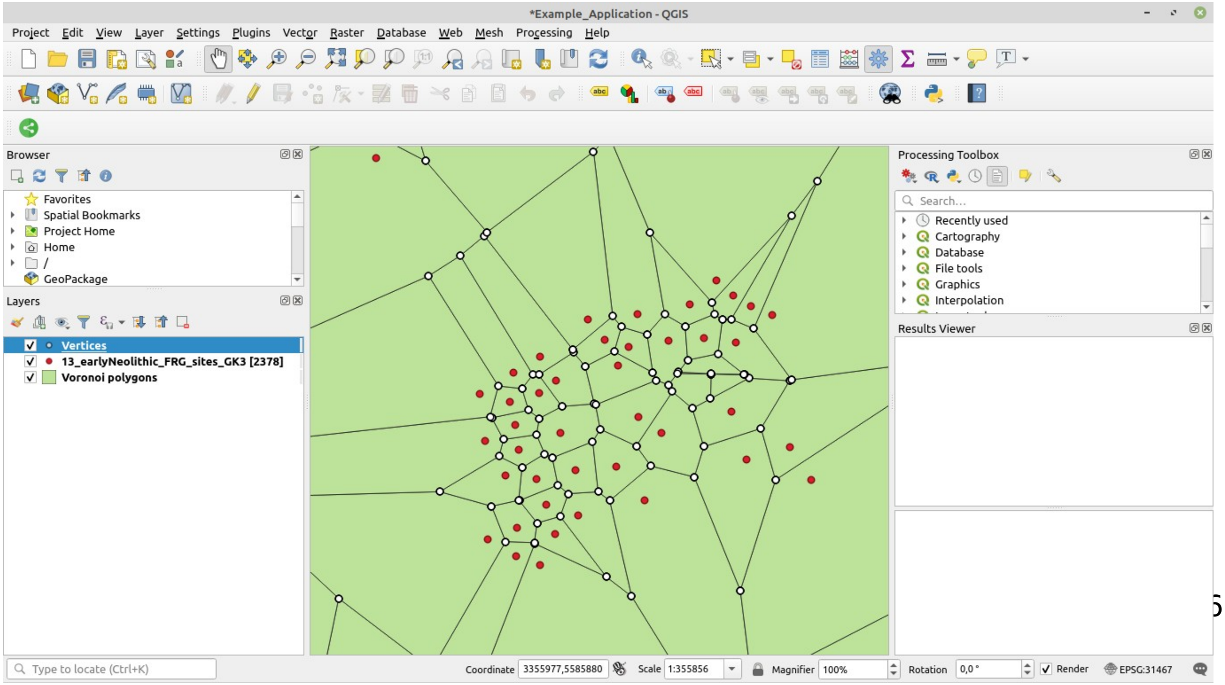

4. Aggregation of vertices

At the vertices/nodes several polygons meet, therefore extracting the nodes has led to duplicate vertices. Those have to be deleted. One possibility for deleting duplicates in QGIS is:

Open Processing Toolbox (Ctrl+Alt+T):

/Processing Toolbox / Vector general / Delete duplicate geometries...
Search for: *Delete duplicate geometries*

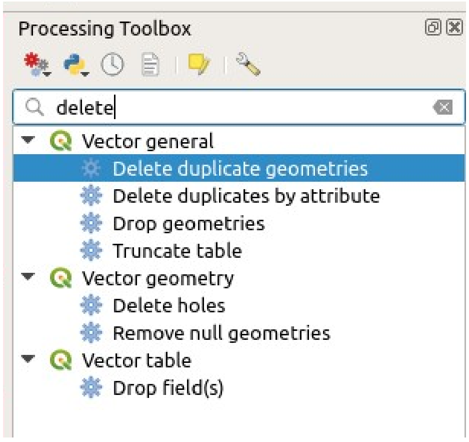

Input layer: Vertices

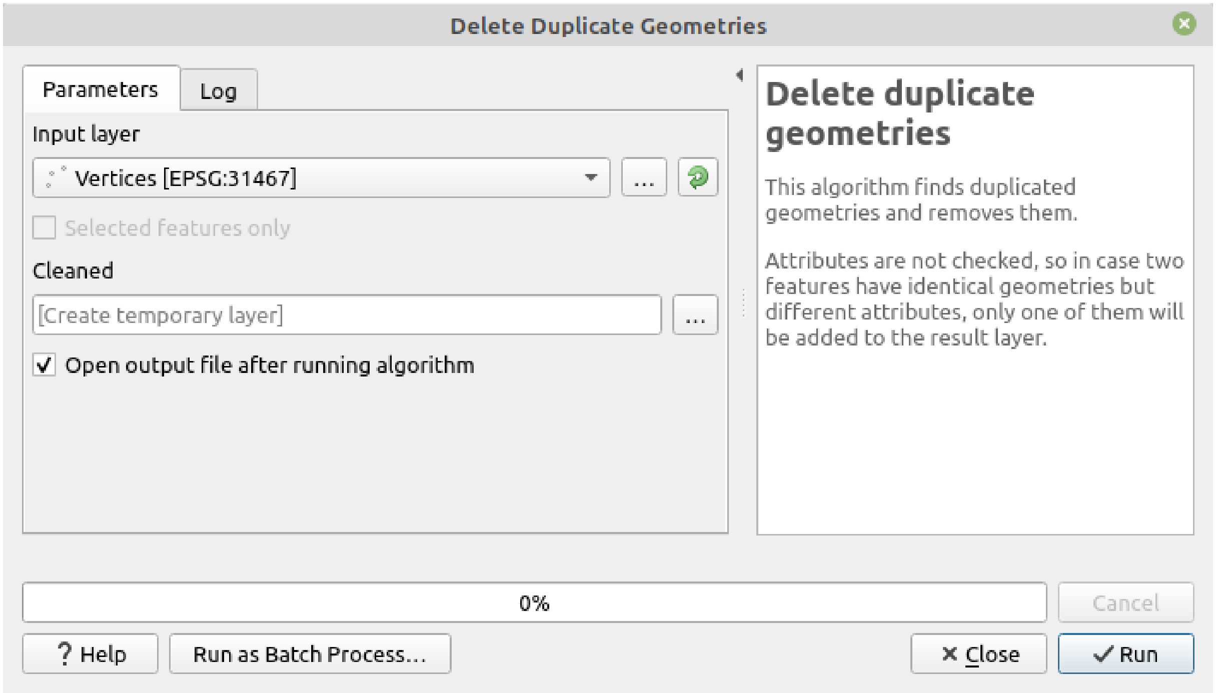

Result: Cleaned.shp

Using the example data you should end up with about 4758 vertices (duplicates deleted). You can add the number of features to the Layers panel by right clicking on the layer and selecting “Show feature count”.

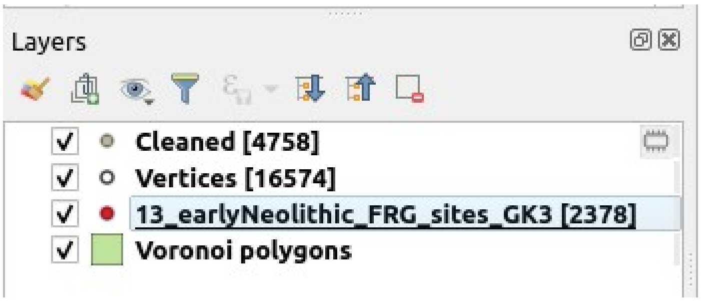

5. Defining the radius of the „Largest Empty Circle“

The distance between vertex/node and the nearest measurement (site) is equal to the radius of the Largest Empty Circle (LEC). We will now measure the distance between vertices and sites (measurements/observations).

/ Processing Toolbox / Vector analysis / Distance to nearest hub (points)...

Source points layer: Shape-File with Vertices (Cleaned)

Destination hubs layer: Shape-File with sites (13_earlyNeolithic...)

Hub layer name attribute: ID Column (_ID)

Measurement unit: Meters

Important: the unit of measurement has to be equal to the project unit of measurement (in our case meter).

This time it is vital to export the results, a temporary layer won’t do because we will be using the LEC shp-File in SAGA. Save results as:

LEC.shp

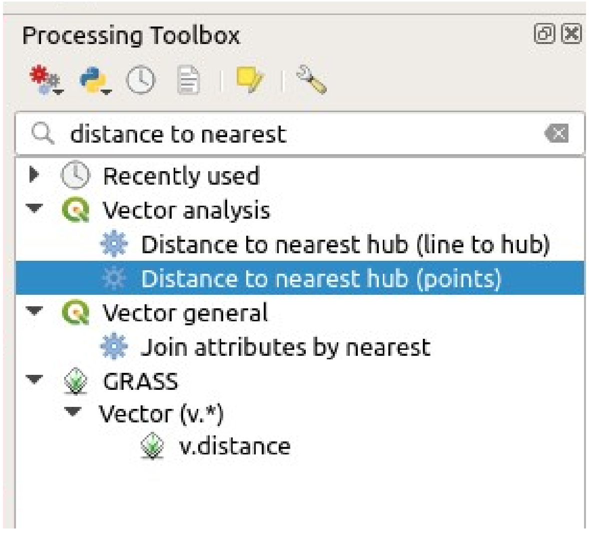

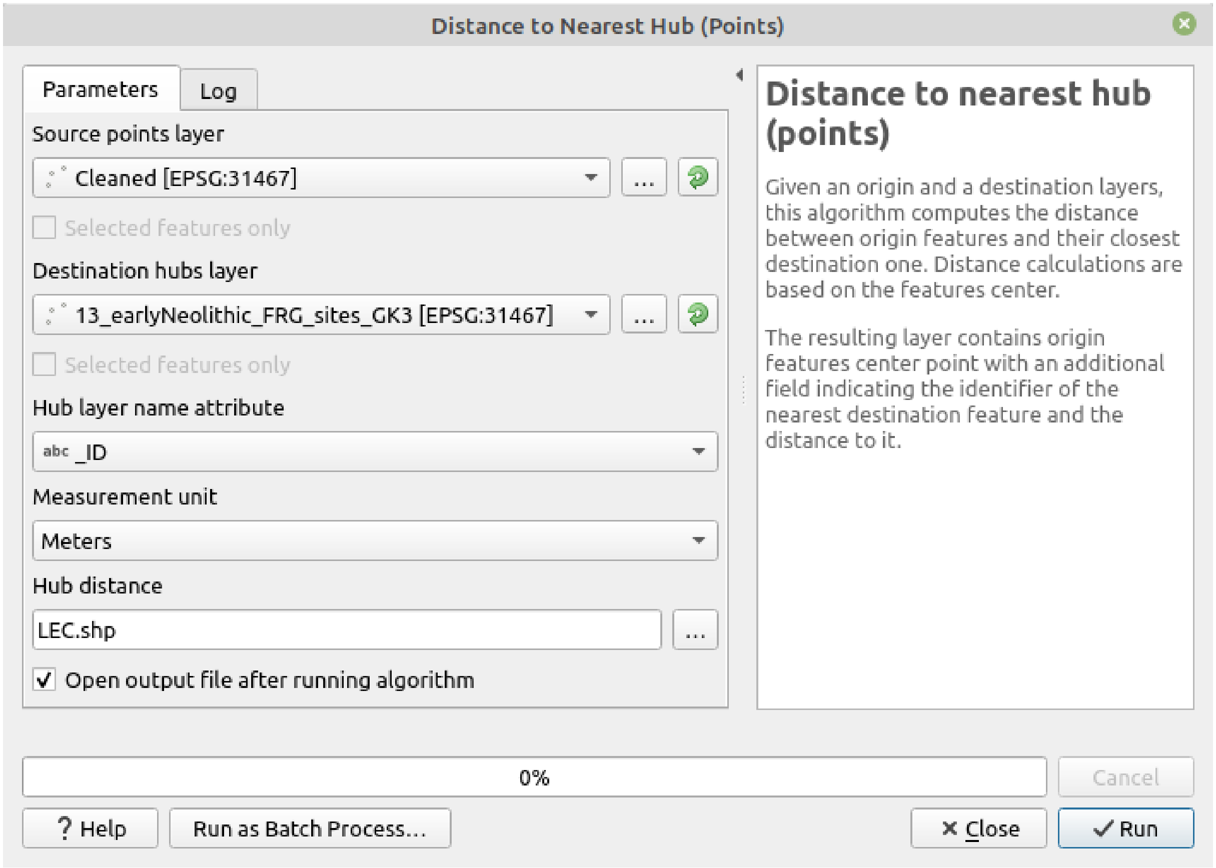

Have a look at the resulting Shape-File:

Choose Layer / Layer / Open Attribute Table...

Among others, you should find following columns:

HubName	ID of the nearest measurement (site)
HubDist	distance between node and measurement (LEC radius)

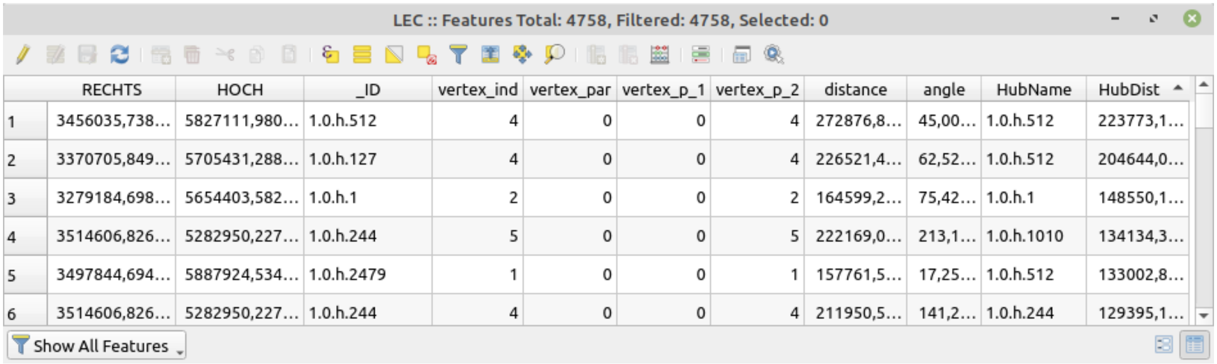

	RECHTS	HOCH	_ID	vertex_ind	vertex_par	vertex_p_1	vertex_p_2	distance	angle	HubName	HubDist
1	3456035,738...	5827111,980...	1.0.h.512	4	0	0	4	272876,8...	45,00...	1.0.h.512	223773,1...
2	3370705,849...	5705431,288...	1.0.h.127	4	0	0	4	226521,4...	62,52...	1.0.h.512	204644,0...
3	3279184,698...	5654403,582...	1.0.h.1	2	0	0	2	164599,2...	75,42...	1.0.h.1	148550,1...
4	3514606,826...	5282950,227...	1.0.h.244	5	0	0	5	222169,0...	213,1...	1.0.h.1010	134134,3...
5	3497844,694...	5887924,534...	1.0.h.2479	1	0	0	1	157761,5...	17,25...	1.0.h.512	133002,8...
6	3514606,826...	5282950,227...	1.0.h.244	4	0	0	4	211950,5...	141,2...	1.0.h.244	129395,1...

The maximum “HubDist” (LEC radius) for our dataset is ca. 223773 m.

To get basic Statistics for LEC-radii:

Vector / Analysis Tools / Basic Statistics for Fields...

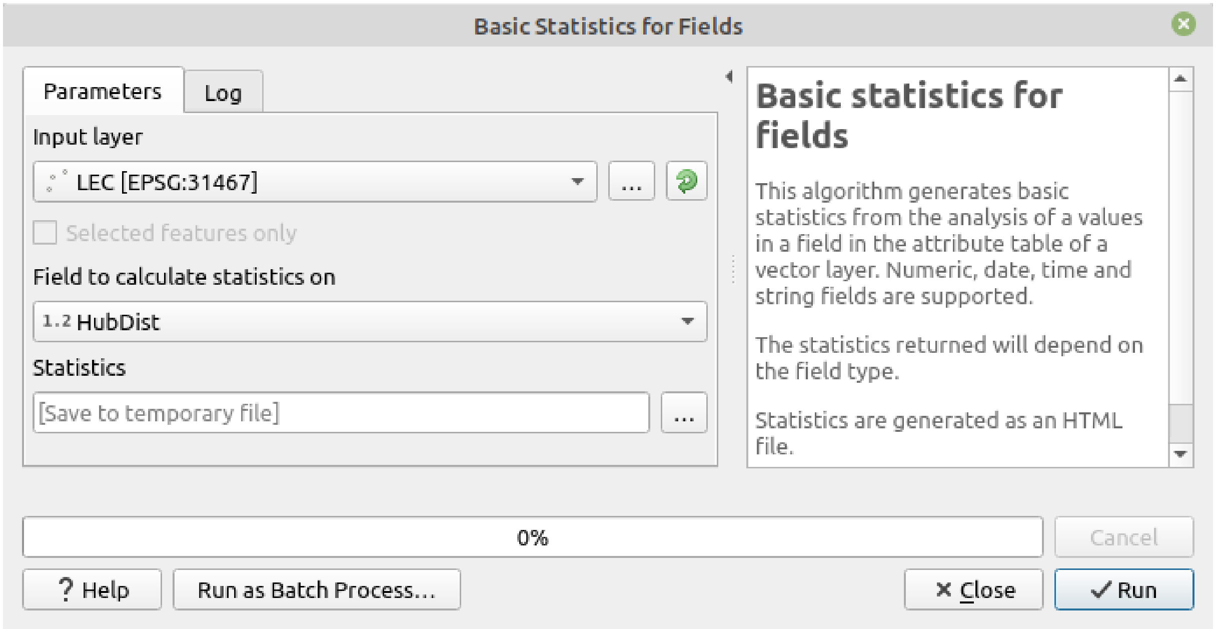
Basic Statistics for Fields
Parameters
Log
Input layer
LEC [EPSG:31467]
...
Selected features only
Field to calculate statistics on
1.2 HubDist
Statistics
[Save to temporary file]
...
Basic statistics for fields
This algorithm generates basic statistics from the analysis of a values in a field in the attribute table of a vector layer. Numeric, date, time and string fields are supported.
The statistics returned will depend on the field type.
Statistics are generated as an HTML file.
0%
Cancel
? Help
Run as Batch Process…
Close
Run

5a Calculate Statistics

The Kriging procedure requires the variables lag distance and maximum search distance. We will calculate these parameters in QGIS and then move on to SAGA to build a semivariogram and compute the interpolation. For the maximum search distance (MaxSearchDist) the diagonal of the LEC bounding box is divided by two. To estimate lag distance (LagDist) we divide the bounding box diagonal by 250. Both values will be used later in SAGA.

First, create the bounding box for the LEC/vertices:

Processing Toolbox / Vector Geometry / Minimum bounding geometry...

Input Layer:	LEC.shp
Field:	“”
Geometry Type:	Envelope (Bounding Box)

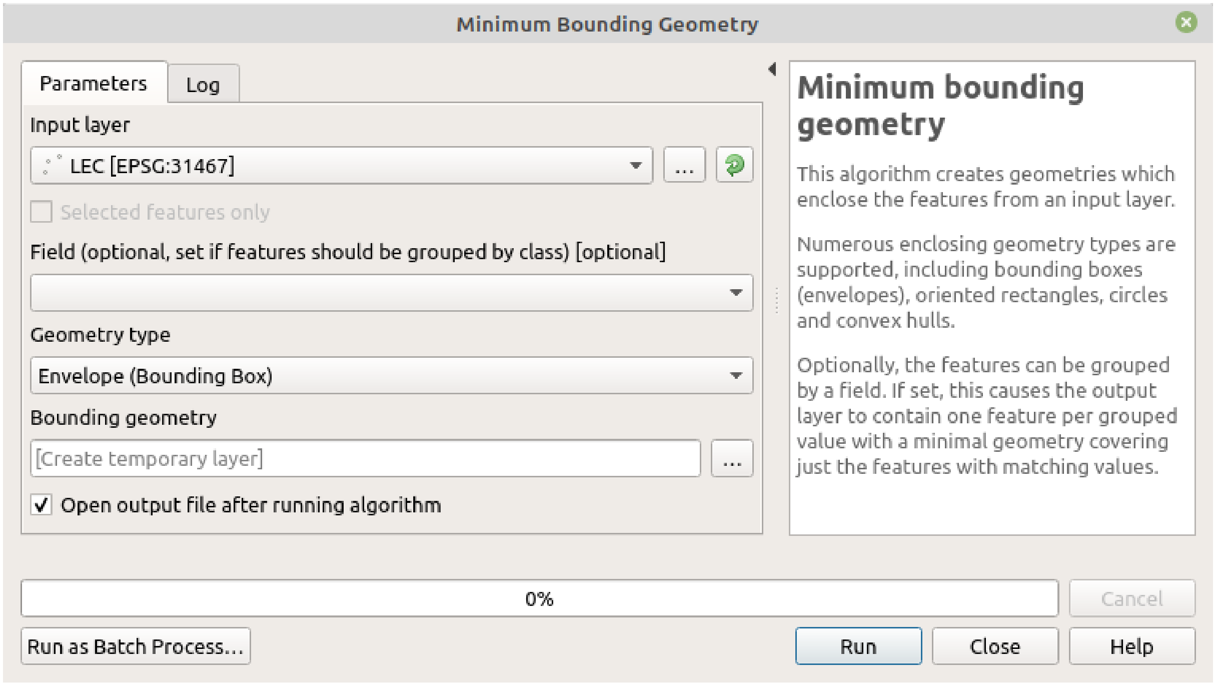

Open Attribute Table of new layer “Bounding geometry” (*Right Click on Layer / Open Attribute Table*) and open Field Calculator (Ctrl+*I*)

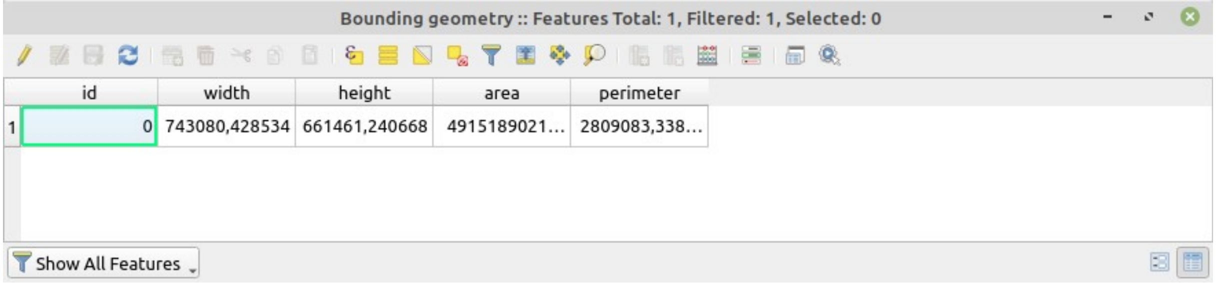

Check “Create a new field”

<u>Output field name:</u> MaxSearchDist
<u>Output field type:</u> Decimal number (real)
<u>Output field length:</u> 10
<u>Precision:</u> 3
<u>Formula:</u> sqrt("width" ^ 2+ "height"^2)/2

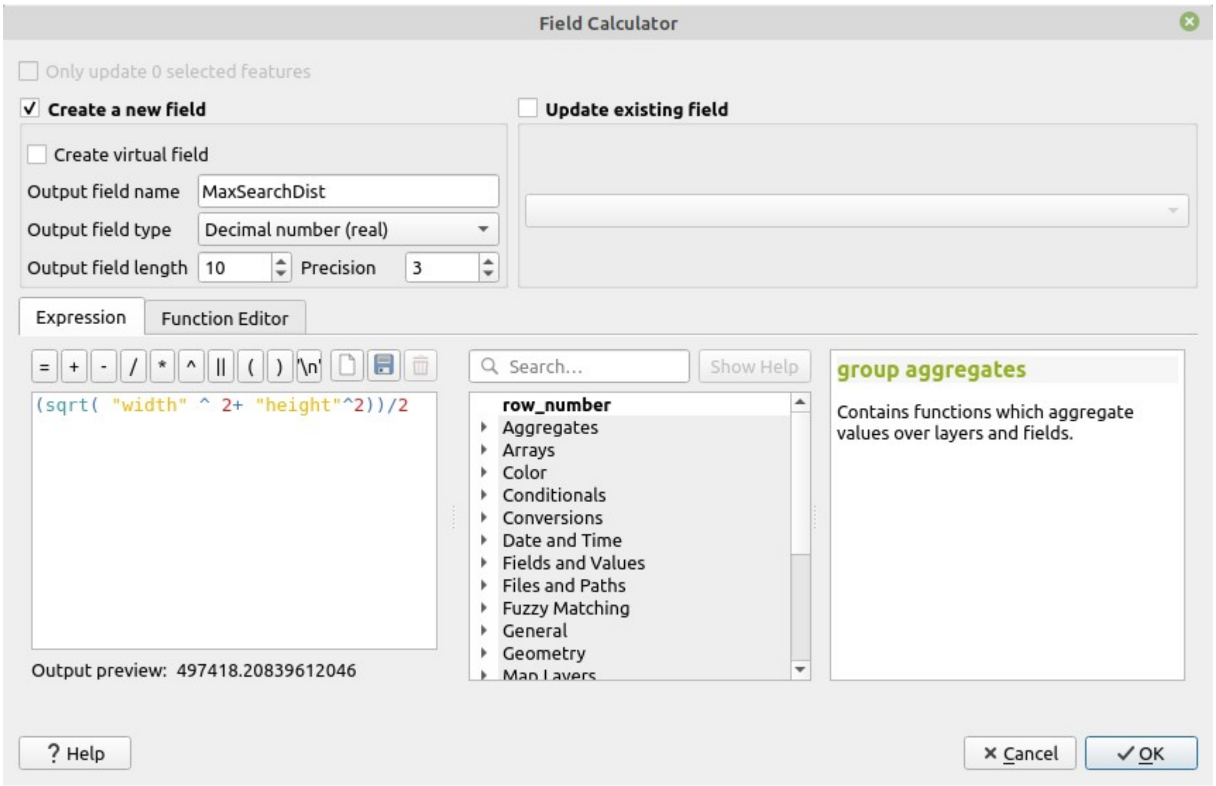

And you should also calculate the Lag Distance to be used in the Semivariogram:

Check "Create a new field"

<u>Output field name:</u> LagDist
<u>Output field type:</u> Decimal number (real)
<u>Output field length:</u> 10
<u>Precision:</u> 3
<u>Formula:</u> *sqrt("width" ^ 2+ "height"^2)/250*

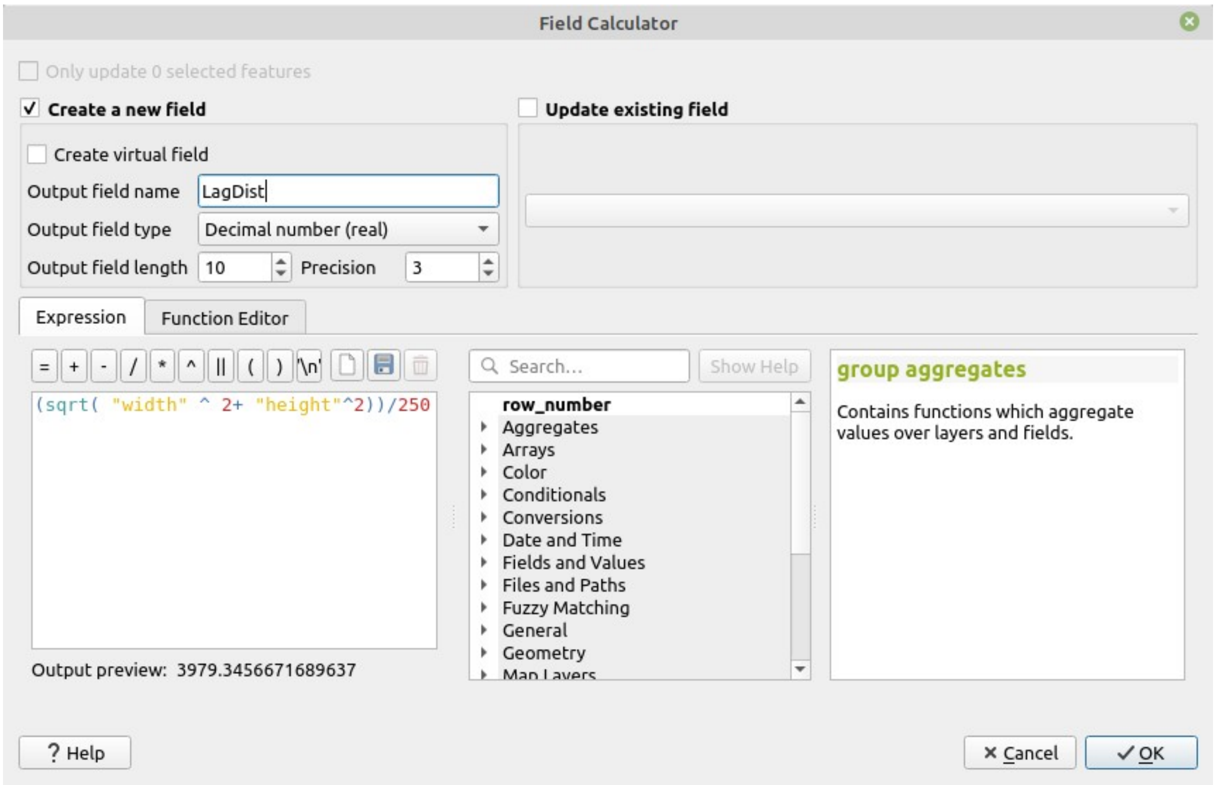
Field Calculator
Only update 0 selected features
Create a new field
Update existing field
Create virtual field
Output field name LagDist
Output field type Decimal number (real)
Output field length 10 Precision 3
Expression
Function Editor
(sqrt("width" ^ 2+ "height"^2))/250
Search...
Show Help
row_number
Aggregates
Arrays
Color
Conditionals
Conversions
Date and Time
Fields and Values
Files and Paths
Fuzzy Matching
General
Geometry
Map Layers
group aggregates
Contains functions which aggregate values over layers and fields.
Output preview: 3979.3456671689637
Help
Cancel
OK

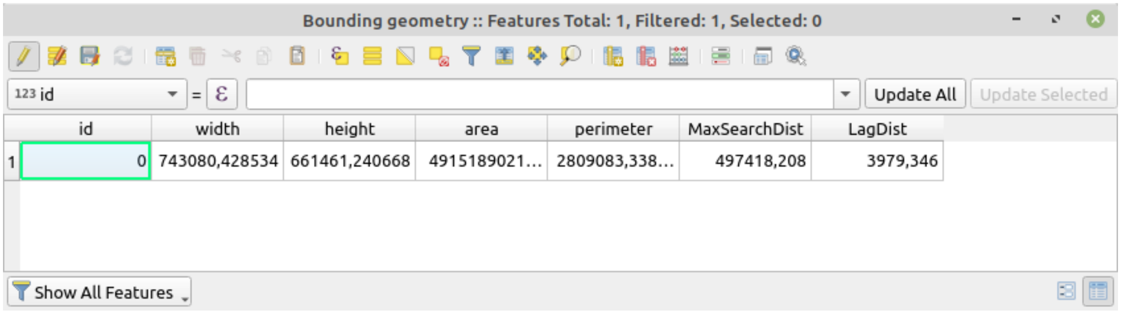
Bounding geometry :: Features Total: 1, Filtered: 1, Selected: 0
123 id
Update All
Update Selected
id
width
height
area
perimeter
MaxSearchDist
LagDist
1
0
743080,428534
661461,240668
4915189021...
2809083,338...
497418,208
3979,346
Show All Features

6. Kriging – Preparations and Grid

Save your results, close QGIS and start SAGA. Open the layers with the LEC-Radii and the layer with initial measurements (sites):

File / Open…

13_earlyNeolithic_FRG_sites_GK3.shp
LEC.shp

Double-clicking on the layer will add the points to a map.

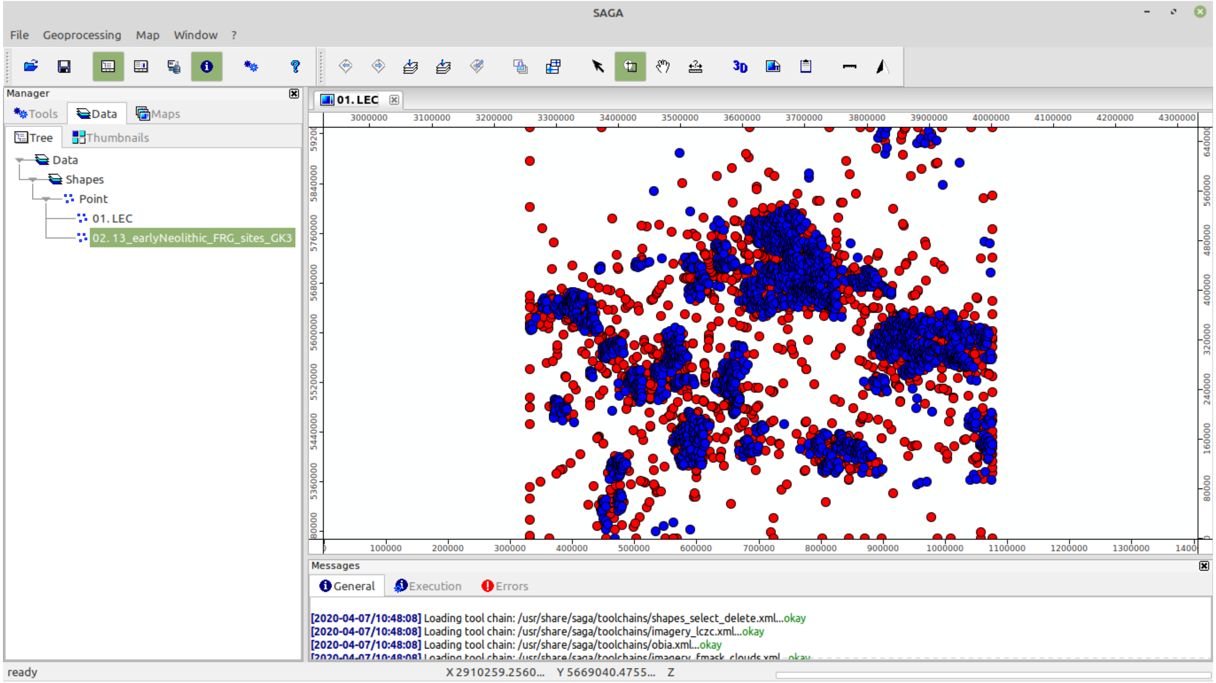

For interpolation, we will be using the Ordinary Kriging Module from SAGA. The parameters for building the semivariogram and for Kriging have to be entered first. For kriging we need a grid of evenly distributed points. The interpolation algorithm will estimate the site distance (radius of LEC) at every point of the grid, based upon the available vertices and their values for the radius of the LEC. For the current example we will create a grid with a spacing of 1000 m between each point. That means a raster file with 1 × 1 km pixels is created.

Geoprocessing / Spatial and Geostatistics / Kriging / Ordinary Kriging…

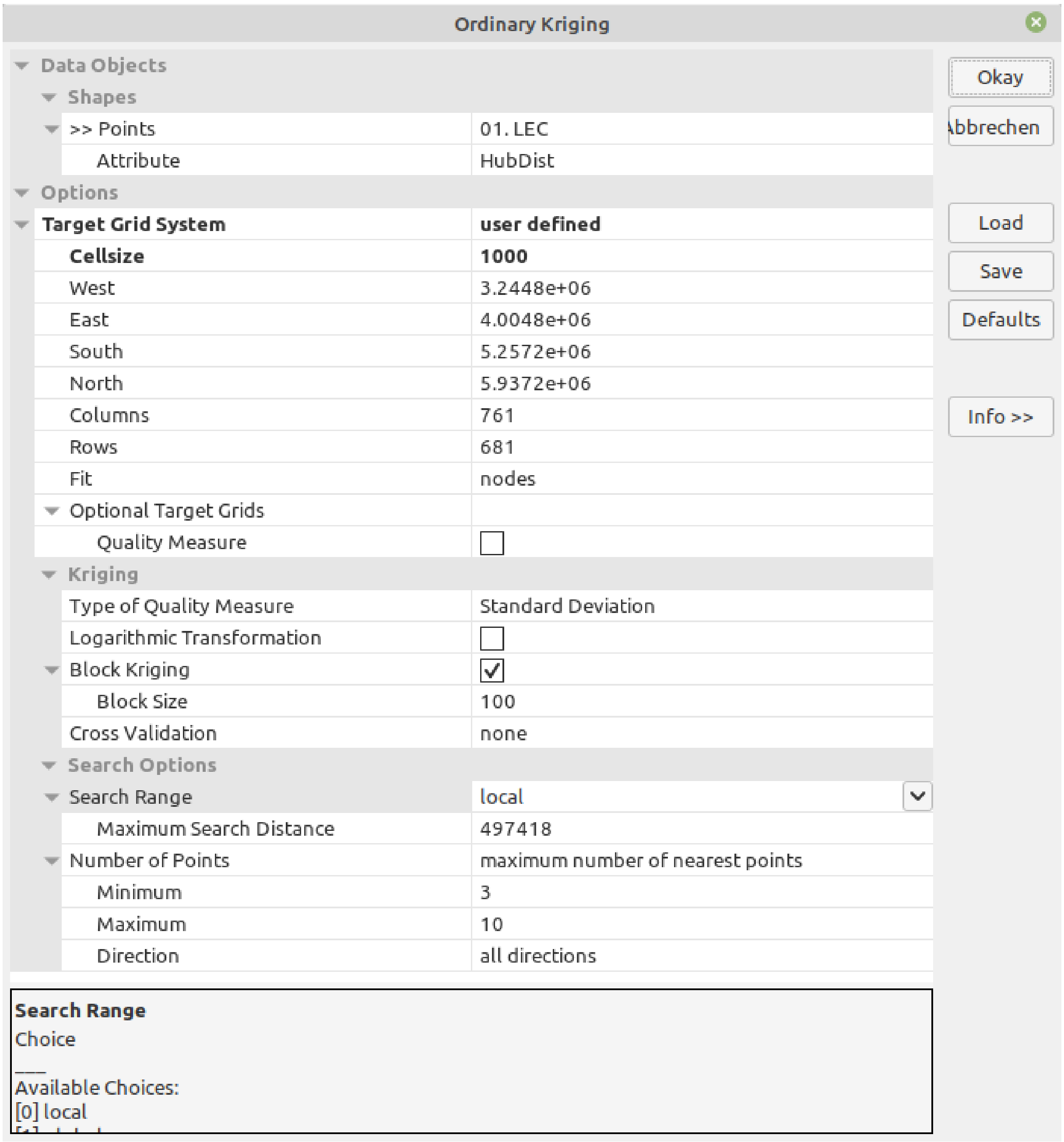
Ordinary Kriging
Data Objects
Shapes
>> Points
01. LEC
Attribute
HubDist
Options
Target Grid System
user defined
Cellsize
1000
West
3.2448e+06
East
4.0048e+06
South
5.2572e+06
North
5.9372e+06
Columns
761
Rows
681
Fit
nodes
Optional Target Grids
Quality Measure
Kriging
Type of Quality Measure
Standard Deviation
Logarithmic Transformation
Block Kriging
Block Size
100
Cross Validation
none
Search Options
Search Range
local
Maximum Search Distance
497418
Number of Points
maximum number of nearest points
Minimum
3
Maximum
10
Direction
all directions
Search Range
Choice
Available Choices:
[0] local
Okay
Abbrechen
Load
Save
Defaults
Info >>

Points:	Layer we want to krige (LEC.shp)
Attribute:	HubDist
Target Grid system:	user defined
Cellsize:	1000
Search Range:	local
Maximum Search Distance:	497418
Number of Points Minimum:	3
Maximum:	10
Direction:	all directions

Check “Quality Measure” to get plot of the variance for checking the quality of the interpolation. You can also activate “Block Kriging” (Block Size: 100) for smoothing. Changing blocksize or not using block kriging won’t alter the results dramatically.

Press Okay to compute the sample semivariogram.

7. Kriging - Semivariogram

The kriging procedure requires a theoretical semivariogram, which is used to estimate the radii of the LEC at every point of the grid. To compute this theoretical semivariogram, we need to explore the experimental semivariogram first. The Semivariogram displays the ratio of distance between pairs of points to the similarity (difference) of points in distance classes (lags).

To adjust lag distance and enter a theoretical model (formula) click:

Settings / Variogram Settings...

Choose one of the following functions, either a

linear regression/exponential model:

a + b * x + c * x ^2

or as an alternative, the power model:

_a + b * x ^c_

Variables explained:
a= nugget
b = sill
c = range

The spherical model doesn’t seem to work, at least using our data set. Make sure to set nugget to zero (a = 0). For example in the exponential model: “0 + b * x + c * x^2” or using the power model “0 + b * x ^c”. Or simply:

Linear regression/exponential model:

b * x + c * x ^2

or as an alternative, the power model:

_b * x ^c_

<u>Skip:</u>	1
<u>Lag Distance:</u>	3979 (calculated in QGIS)
<u>Maximum Distance:</u>	497418 (= MaxSearchDist, calculated in QGIS)
<u>Model:</u>	b * x ^c<br>_or_<br>b * x + c * x ^2

Enter the model of your choice in “Model” and click “Okay” to return to the semivariogram.

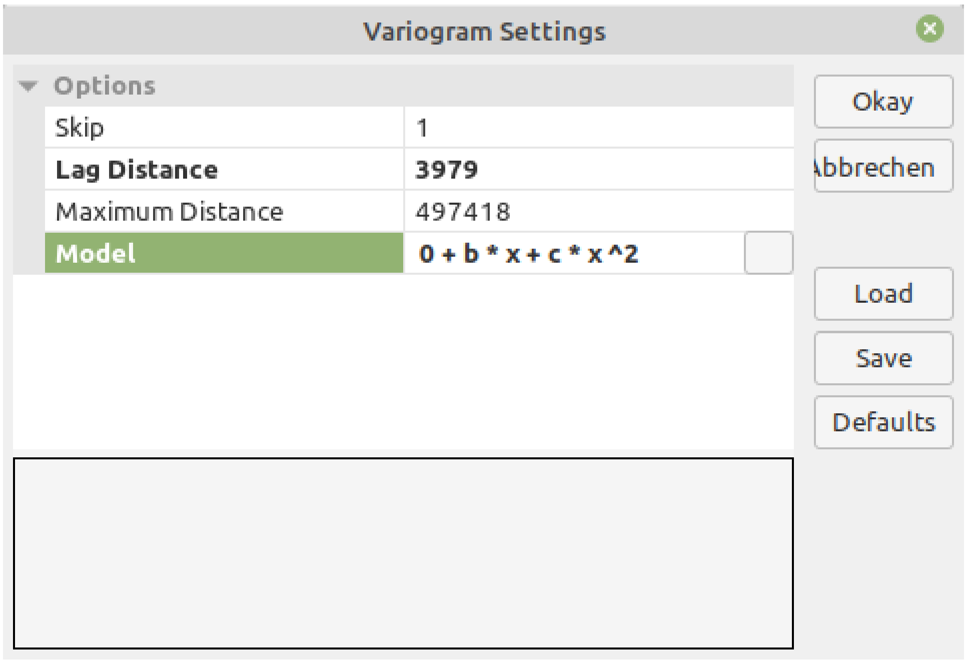

Adjust the fitting range to the first peak or plateau of the distribution by using the slider “Function Fitting Range” on the left side of the panel. In our case study it is at ca. 79586 on the x-axis (Fitting range = 79586.9). Of course, a decision has to be made on a case-by-case basis.

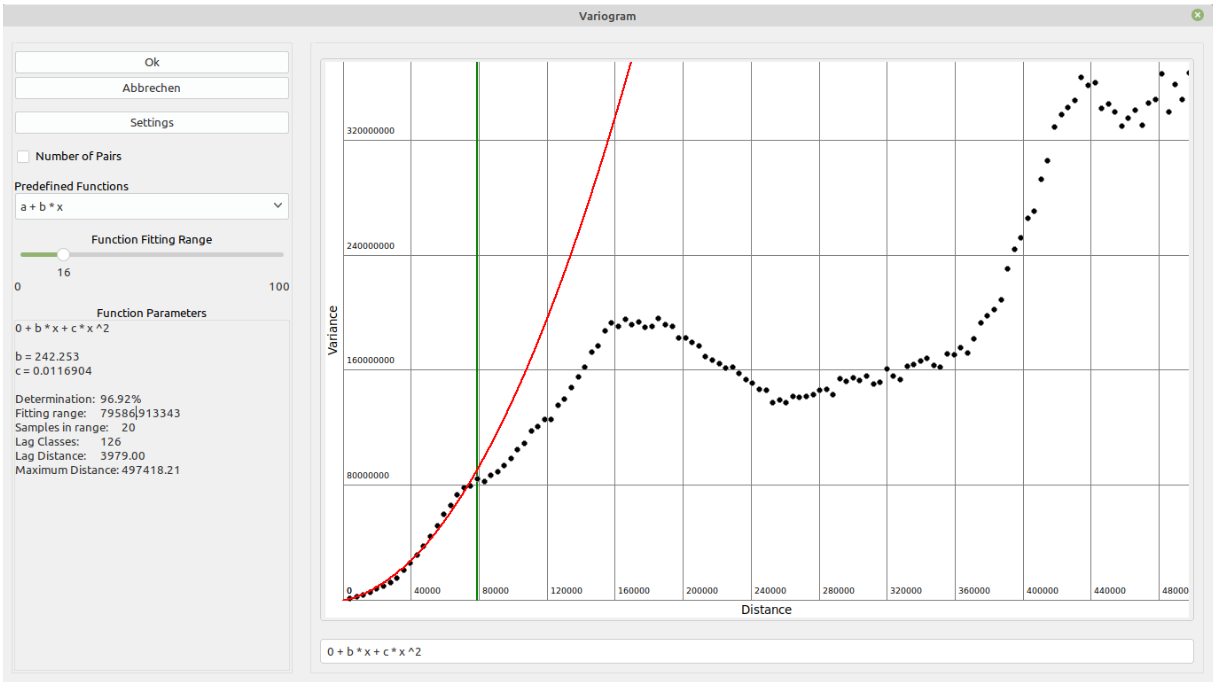

Click OK to start the Kriging process. Computing will take some while depending on your computer (processor, ram, hard disk).

8. Kriging - inspect and export raster output

The result of the kriging interpolation is a raster map of the prediction and, if you checked “Quality Measure”, the variance or standard deviation of the results. Double-click on the new grid to add it to a map.

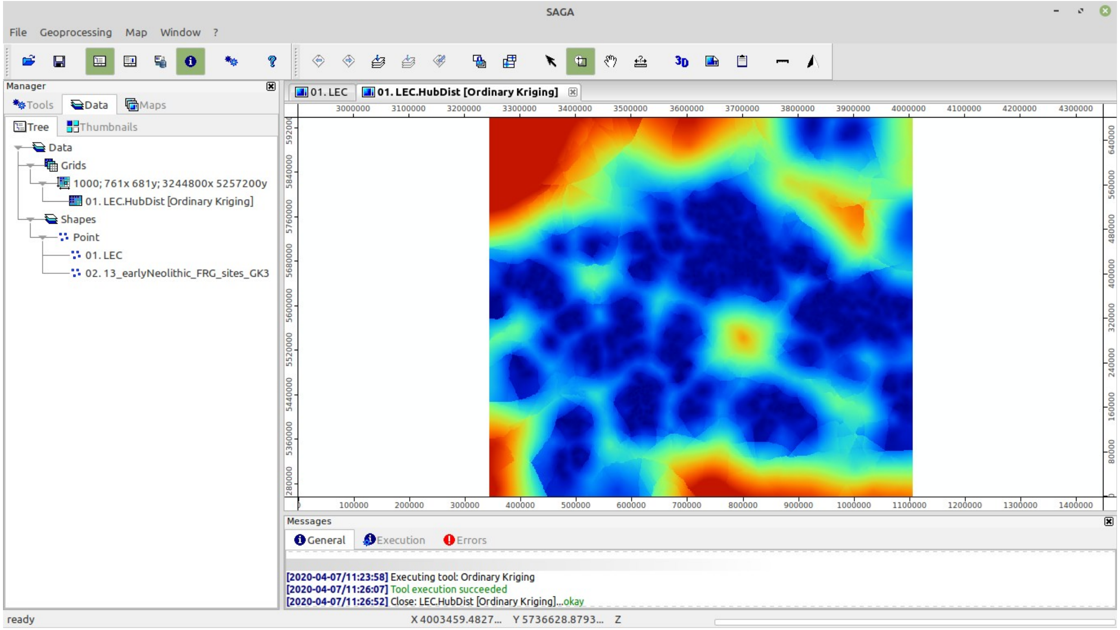

You can export the raster as Surfer Grid so you can import it in QGIS later.

Geoprocessing / File / Grid / Export / Export Surfer Grid...

9. Creating contour lines (isolines)

Using the kriging raster we will create isolines, convert them to polygons and compute the area and number of sites per isoline. Number of sites and isoline area are important for selecting the “Optimally Describing Isoline” (*Zimmermann et al. (2004, 53-55)*).

First we will extract the isolines or contour lines from the raster. The easiest way to find functions in SAGA is to search for them:

Geoprocessing / Find and run tool / Contour Lines from Grid...

or

Geoprocessing / Shapes / Grid / Vectorization / Contour Lines from Grid...

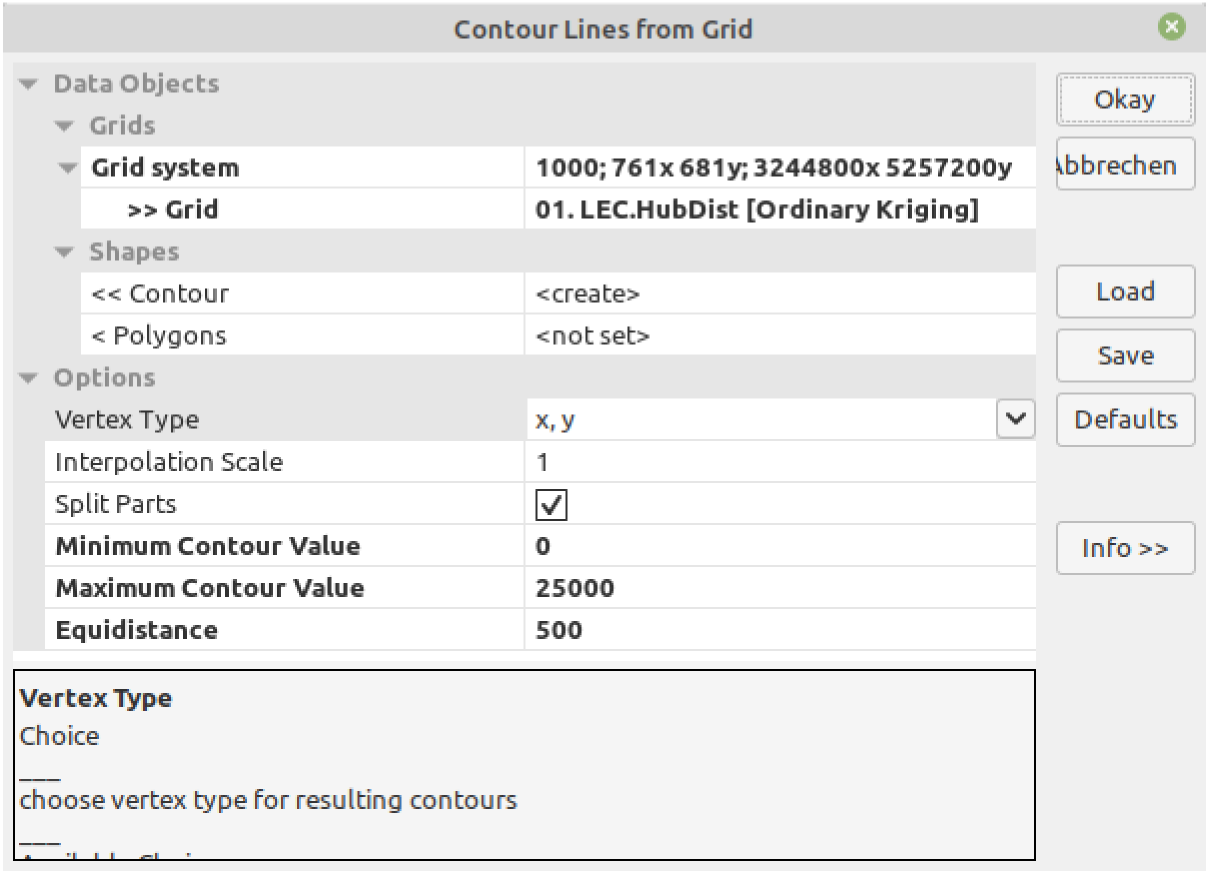

In our case study we need contour lines starting at 0 and ending at 25000 m with an equidistance of 500 m. It is advised to calculate as few isolines as possible.

Grid:	our new Kriging grid (the original not the export)
Contour:	<create>
Polygons:	<not set> (we want lines not polygons yet)
Vertex Type:	x, y
Interpolation Scale:	1
Split Parts:	checked
Minimum Contour Value:	0

Maximum Contour Value: 25000
Equidistance: 500

Geoprocessing / Shapes / Conversion / Convert Lines to Polygons…

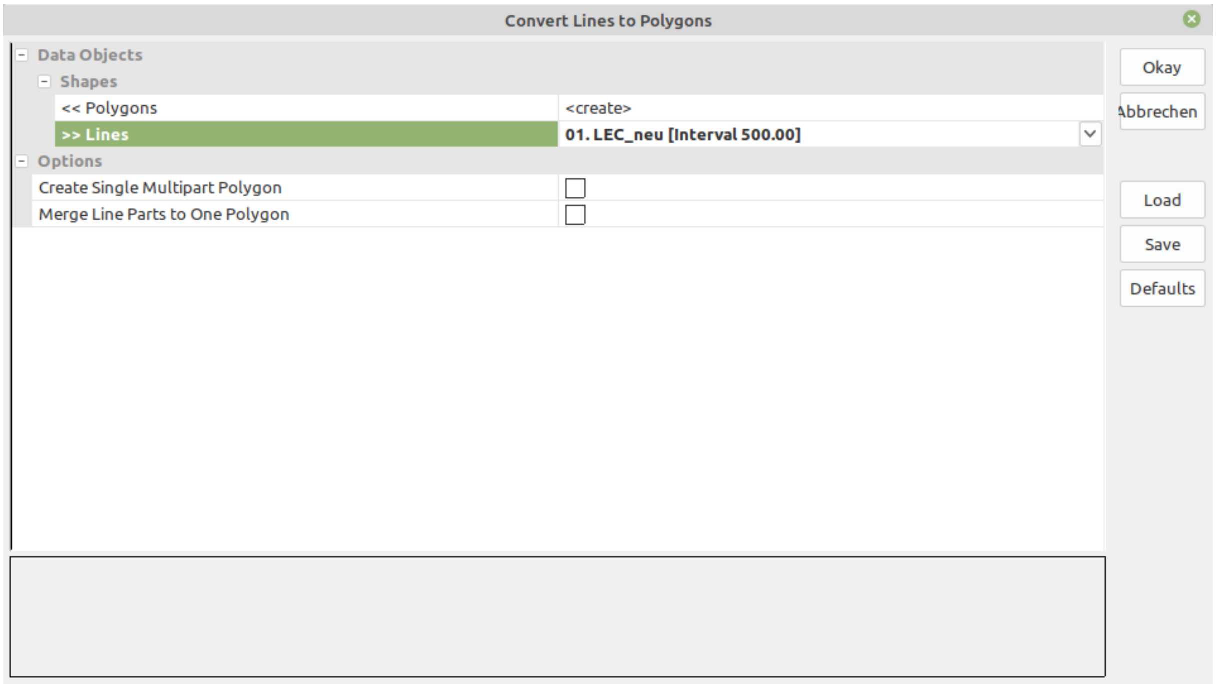

Polygons:	<create>
Lines:	the contour line layer created above
Create Single Multipart Polygon:	unchecked
Merge Line Parts to One Polygon:	unchecked

Dissolve Polygons using the iso-value (Z):

Geoprocessing / Shapes / Polygons / Polygon Dissolve...

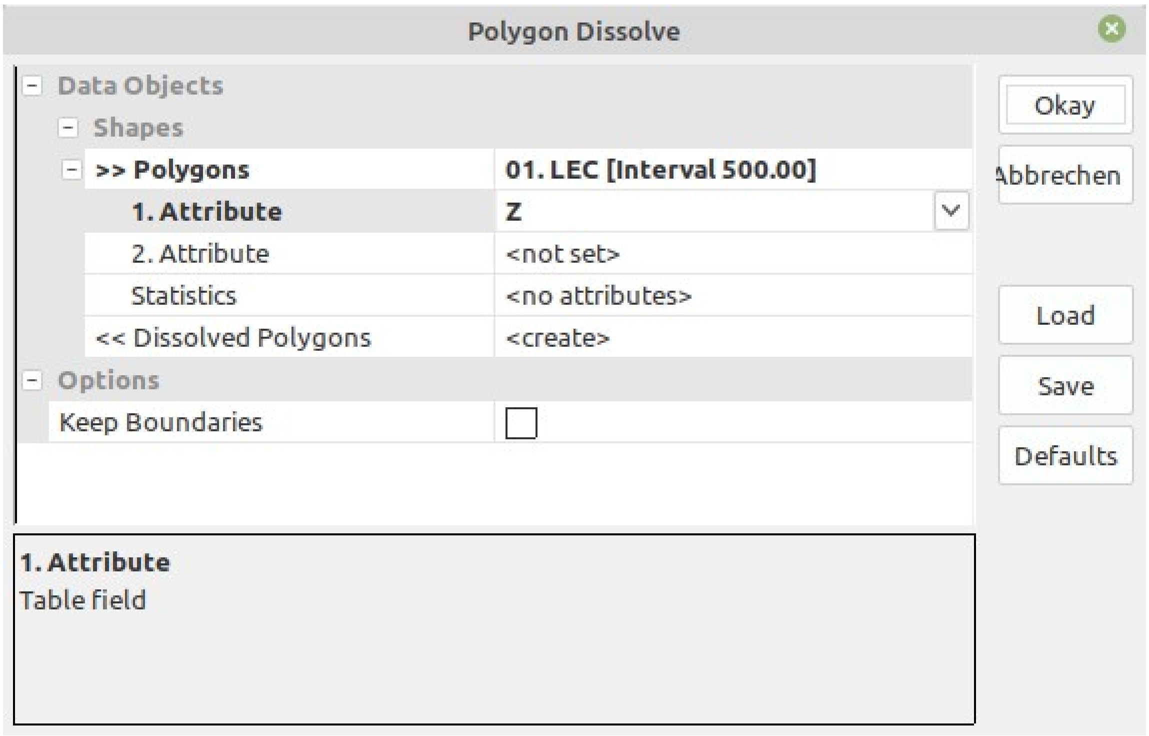

<u>Polygons:</u>	Polygon layer created above
<u>1. Attribute:</u>	Z
<u>2. Attribute:</u>	<not set>
<u>Statistics:</u>	<no attributes>
<u><<Dissolved Polygons:</u>	<create>
<u>Keep Boundaries:</u>	unchecked

10. Calculating the area and the number of sites per isoline

Use the newly created dissolved isoline polygon layer and check the box “Area” and “Number of Parts”

Geoprocessing / Shapes / Polygons / Polygon Properties...

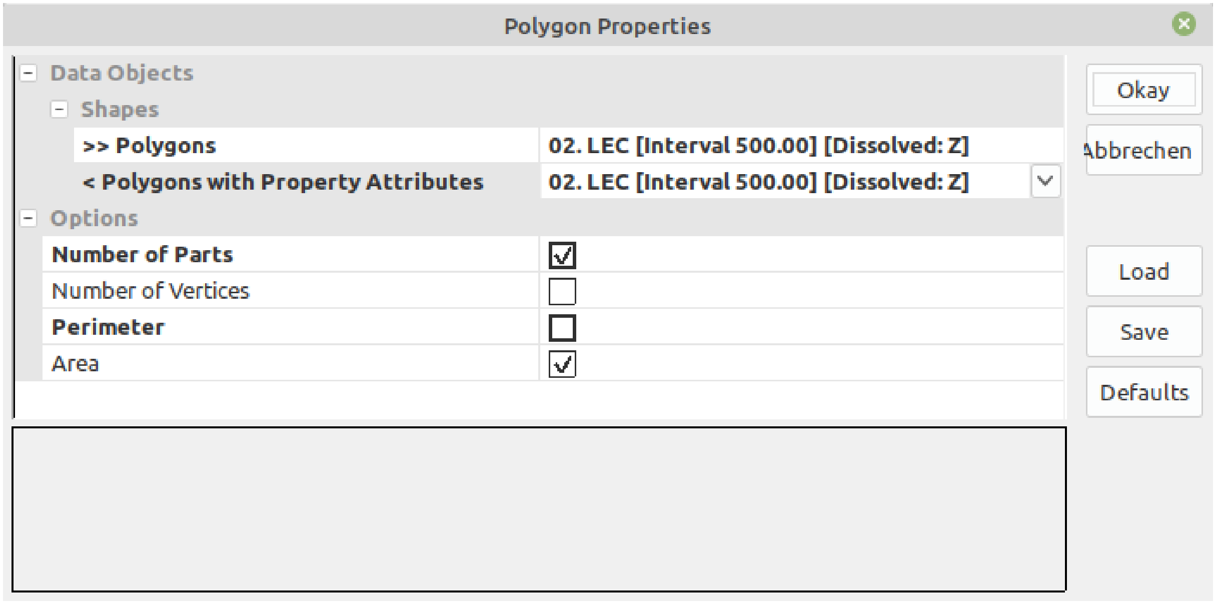

Polygons:	Dissolved polygon layer created above
Polygons with Property Attributes:	Dissolved polygon layer created above
Number of Parts:	checked
Number of Vertices:	unchecked
Perimeter:	unchecked
Area:	checked

The calculated areas will be in your map unit (in our case study: m^2). If we want km^2 we have to:

Processing / Table / Calculus / Table Calculator...

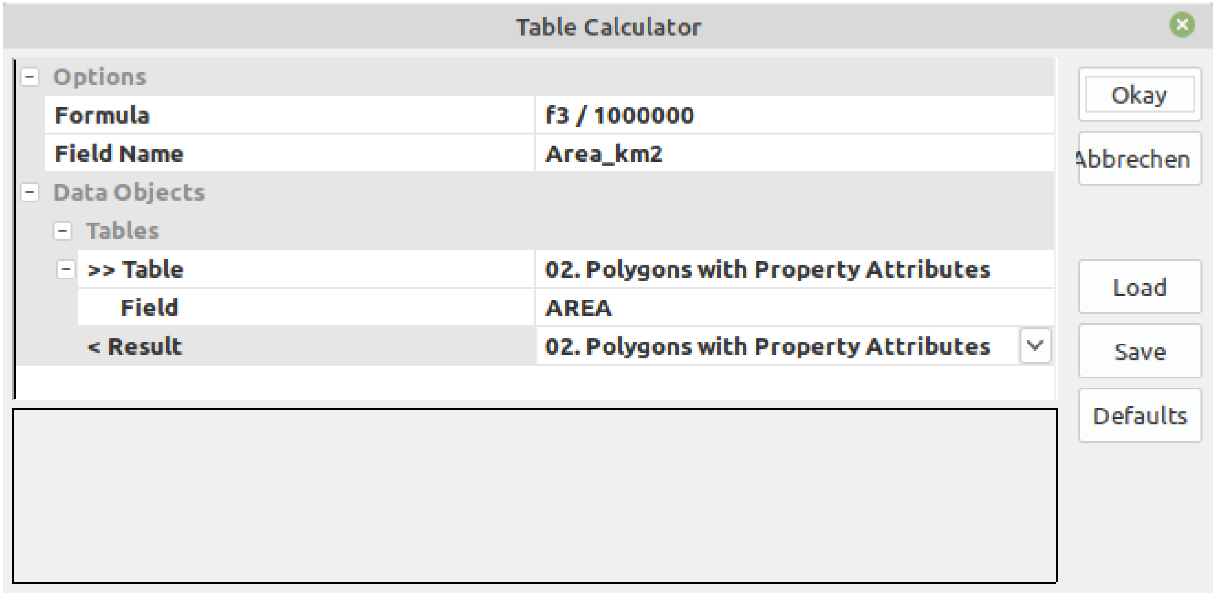

Formula: f3 / 1000000 (third field of the table)
Field Name: Area_km2 (name of field to be created)
>>Table: Polygon layer with attributes
Field: AREA (field to be divided)
<Result: Polygon layer with attributes (update layer)

Geoprocessing / Shapes / Points / Points Count Points in Polygons

Now we count the number of sites within each isoline. “Points” is the original layer with measurements (sites) and “Polygons” is the dissolved Isoline polygon layer (might be called “Polygons with Property Attributes” or “Result”).

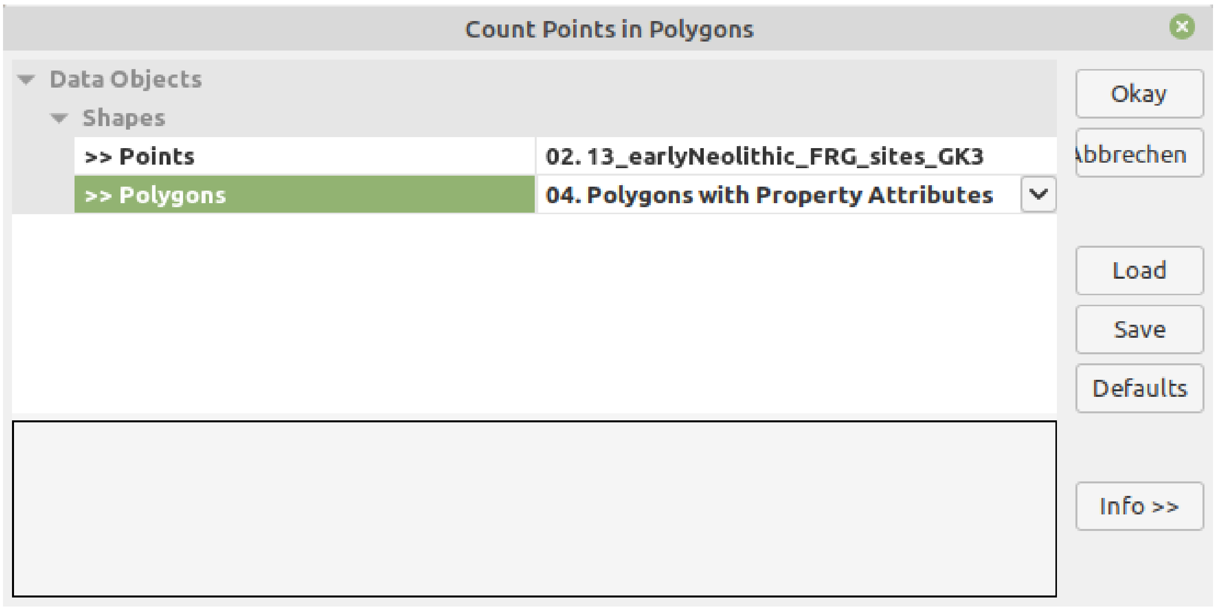

Have a look at the attributes:

Right Click on Layer > Attributes > Show

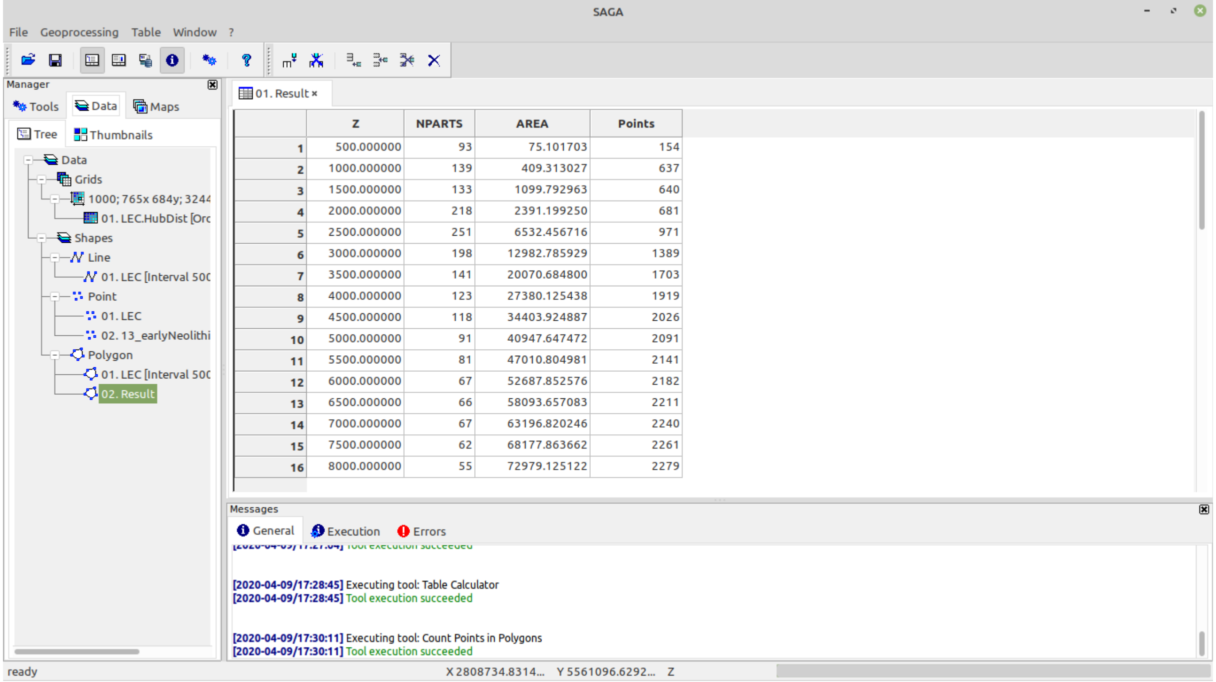

You should end up with a shape-file of polygons and columns for each isoline value (“Z”), the number of parts the isolines consist of (“NPARTS”), the area per isoline and the number of sites (“Points”) within each isoline. The figure above shows the results using the first formula (linear regression/exponential model).

11. Data export

Export the results (might be called “Polygons with Property Attributes” or “Result”) as comma-separated values (*.csv):

Geoprocessing / File / Tables / Export / Export Text Table...

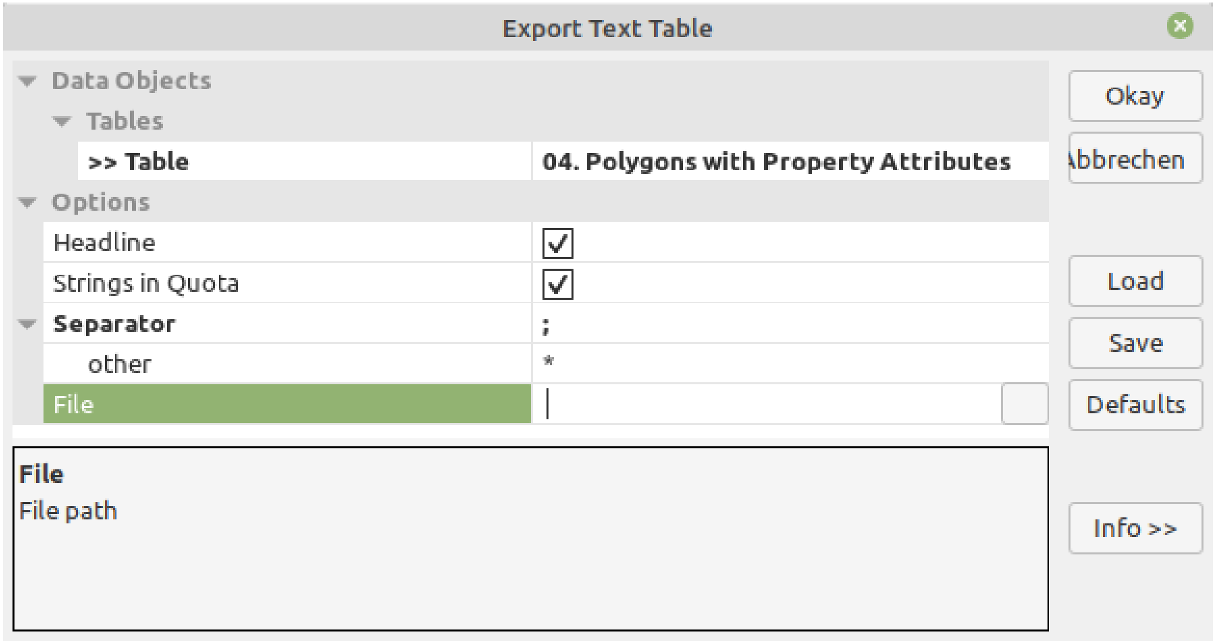

and as shape-File:

Geoprocessing / File / Shapes / Export / Export Shapes

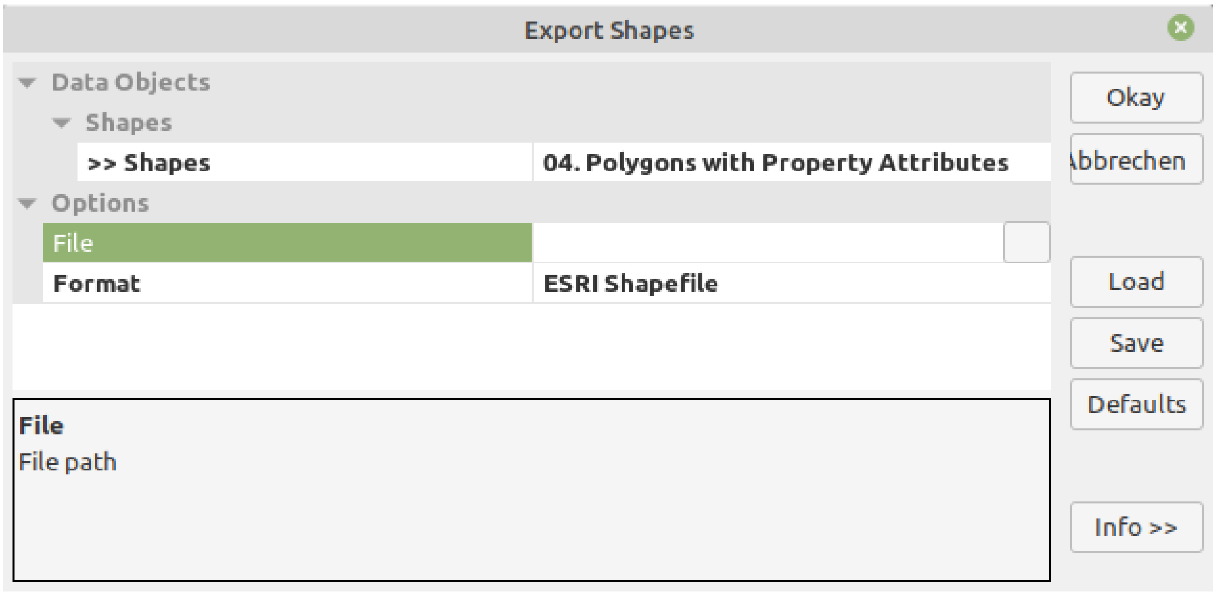

12. Selecting the "Optimally Describing Isoline"

The step of selecting the Optimally Describing Isoline is not described in this manual. You can do this in a spreadsheet program (e.g. Microsoft Excel). Please refer to the R or the MapInfo manual.

Bibliography

Preuss, J. (ed.), **1998**. Das Neolithikum in Mitteleuropa: Kulturen, Wirtschaft, Umwelt vom 6. bis 3. Jahrtausend v. u. Z., Übersichten zum Stand der Forschung. Beier & Beran: Weissbach.

Schmidt, I., Hilpert, J., Kretschmer, I., Peters, R., Broich, M., Schiesberg, S., Vogels, O., Wendt, K. P., Zimmermann, A., Maier, A., **2020**. Approaching Prehistoric Demography: Proxies, Scales and Scope of the Cologne Protocol in European contexts. *Philosophical Transactions of the Royal Society B*.

Zimmermann, A., Richter, J., Frank, T., Wendt, K.P., **2004**. Landschaftsarchäologie II. Überlegungen zu Prinzipien einer Landschaftsarchäologie. *Bericht der Römisch-Germanischen Kommission* 85, 37–96.

Zimmermann, A., Wendt, K.P., Frank, T., Hilpert, J., **2009**. Landscape Archaeology in Central Europe. *Proceedings of the Prehistoric Society* 75, 1-53.
