# Supplementary material for: Approaching prehistoric demography: proxies, scales and scope of the Cologne Protocol in European contexts
Source: Philos Trans R Soc Lond B Biol Sci. 2020 Nov 30;376(1816):20190714. doi: 10.1098/rstb.2019.0714 (PMC7741091; doi:10.1098/rstb.2019.0714)
Supplement: Manual S04: Script to model ‘Core-Areas’ (Optimally Describing Isolines) in R [file rstb20190714supp5.pdf]

Supplementary file for:

Approaching Prehistoric Demography: Proxies, Scales and Scope of the Cologne Protocol in European contexts

Schmidt, Isabell; Hilpert, Johanna; Kretschmer, Inga; Peters, Robin; Broich, Manuel; Schiesberg, Sara; Vogels, Oliver; Wendt, Karl Peter; Zimmermann, Andreas; Maier, Andreas

Script and example application to model 'Core-Areas' (Optimally Describing Isolines) using R

Manual Broich & Robin Peters
15 Mai 2020

Introduction

This manual presents an example application of the **Cologne Protocol**. It is based upon the `.R` files which are in the `code\` directory of the GitHub repository CologneProtocol-R (https://github.com/C-C-A-A/CologneProtocol-R). If you want to apply the Cologne Protocol on your own data, we recommend you to use these files, as it is possible to individually adjust certain variables at the beginning of the `00_LEC.R` file. However, the code chunks shown in this manual originate from the mentioned `.R` files. It is possible that the files of the GitHub repository have changed, due to improvements.

This modelling approach constitutes the first of two successive tasks within the 'Cologne Protocol' to estimate past population sizes and densities, described in more detail elsewhere (Schmidt et al. 2020: S2.1. and S2.2.). The manual outlines the technical implementation of working steps 1 to 12 (see Schmidt et al. 2020: Table S2): Firstly a GIS-analysis of site distributions and secondly the identification of the ODI. The working steps include the construction of Voronoi diagrams and "Largest Empty Circles", kriging, converting the kriging results into isolines and finally calculating the criteria to select the ODI.

The goal of the script is to carry out all steps of the first two parts of the Cologne Protocol in R. These are (cf. *Schmidt et al. 2020, table S2*):

Working steps:
1. Loading data (Shape-layer with sites as points)
2. Creating Voronoi Diagram
3. Extraction of Vertices
4. Aggregation of Vertices
5. Defining the Radius of the “Largest Empty Circle”
6. Kriging - Preparation and Grid
7. Kriging - Semivariogram
8. Kriging - inspect and export raster output
9. Creating Contour Lines (Isolines)
10. Calculating the Area and Number of Sites per isoline
11. Data export
12. Selecting the “Optimally Describing Isoline”

Dependencies

To run the code of the script several packages are needed. These dependencies are stored in the `deps.yaml` file. It is possible to load them manually or to use `automagic::install_deps_file()` function. If the package `automagic` is not installed, it is possible to install it with `install.packages("automagic")`.
Furthermore, the script was developed under R version 3.6.3.

Working step 1: Loading data (Shape-layer with sites as points)

For this exemplary application, we use a distribution map of the Early Neolithic Linear Pottery Culture (LBK) in Central Europe. The map is based on *Preuss (1998, Karte 1)* and available from the CRC 806 database (https://crc806db.uni-koeln.de/start/). Besides point symbols representing single sites the original map also included symbols for an agglomeration of five sites. The digital data set has been processed to resolve this issue.

The distribution map can be loaded into R via an URL:

```
# url_link of distribution map of Linear Pottery Culture
url_link <- "http://sfb806srv.uni-koeln.de/owsproxy.php?
service=WFS&version=1.0.0&request=GetFeature&typeNames=geonode
%3A_13_earlyneolithic_ce_sites_wgs84&outputFormat=csv"

# load date as a data.frame
sites <- read.csv(url(url_link))

# Conversion into SPDF
sites <- sp::SpatialPointsDataFrame(sp::SpatialPoints(
  cbind(sites$RECHTS, sites$HOCH)),
  sites,
  proj4string = CRS("+init=epsg:31467"))

# This is not a reprojection, but some functions cause problems if the
```

```
projection is not defined in this way
sp::proj4string(sites) <- sp::CRS("+init=epsg:31467")
```

It is important to note, that cartesian coordinates (planar coordinates) have to be used, because `rgeos::gDistance` will not accept elliptical coordinates. Especially Lat/Long-coordinates will cause a problem.

We can also plot the data:

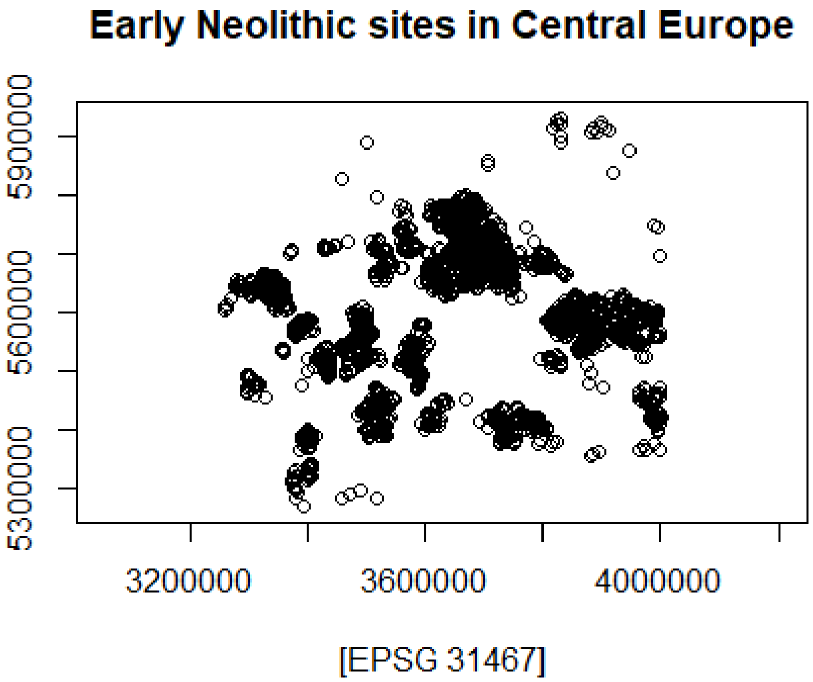

Largest Empty Circle

The “Largest Empty Circle” (LEC) or more precisely the radius of a LEC is a measure of site distance. Every LEC has its center at a vertex of a Thiessen polygon (e.g. Voronoi diagram) and exactly three sites are located on every circumference of a LEC. An illustration can be found at *Zimmermann et al. (2004, Abb.5)*.
To summarize, areas with larger site distances will be characterized by larger radii of LECs and, logically, areas with smaller site distances will be characterized by smaller radii of LECs.

Working steps 2 and 3: Creating Voronoi diagrams and Extraction of vertices

The first step is to calculate the Voronoi diagram and to extract the vertices:

```
# calculate vertices of voronoi diagram
voronoi_vertices <- deldir::deldir(sites@coords[, 1],
                                   sites@coords[, 2],
```

```
                                   rw=c(t(sites@bbox)[1,1],
                                        t(sites@bbox)[2,1],
                                        t(sites@bbox)[1,2],
                                        t(sites@bbox)[2,2])) %$%
  # extraction of vertices
  dirsgs
```

After extracting the vertices from the Voronoi diagram, they have to be prepared to enable a transformation into an `SpatialPointsDataFrame` (spdf):

```
# rearrange voronoi_vertices in preparation for transformation into an spdf
voronoi_vertices <- rbind(setNames(voronoi_vertices[,c(1:2, 5, 7, 9)],
                                   c("x", "y", "ind", "bp", "thirdv")),
                          setNames(voronoi_vertices[,c(3:4, 6, 8, 10)],
                                   c("x", "y", "ind", "bp", "thirdv")))
```

Finally, it is possible to transform the vertices into a spdf. Please note that the projection is explicitly assigned to the newly created `vertices_spdf`, since we have found that otherwise problems can arise:

```
# transformation of voronoi_vertices into an spdf
vertices_spdf <- sp::SpatialPointsDataFrame(coords = voronoi_vertices[1:2],
                                            data = voronoi_vertices[, 3:5])

# Ignore warining! It's not a reprojection. But some functions need this step.
sp::proj4string(vertices_spdf) <- sp::CRS("+init=epsg:31467")
```

Working step 4: Aggregation of vertices

During the step of extracting vertices we didn't take care of vertices duplicates. In addition, some of the vertices are located at the border of the working area, the so-called border points. These points create artificial site distances and it must be decided on a case-by-case basis whether the border points should be deleted or not.
In the present example application we have to remove the duplicates, but we will keep the border points.

```
remove_border_points <- FALSE # Normally, this variable is defined in the
header of 00_LEC.R

# If Condition wether border points are removed or not
if(remove_border_points == TRUE){
# remove dublicates and border points
vertices_spdf <- sp::remove.duplicates(vertices_spdf) %>%
  {.[.[[2]] == FALSE, ]}
} else {
  # just remove dublicates
  vertices_spdf <- sp::remove.duplicates(vertices_spdf)
}
```

Working step 5: Defining the radius of the "Largest Empty Circle"

In the following step we calculate the distance between a vertex of the Voronoi diagram and its nearest site. This distance is the radius of a LEC and will be assigned to the corresponding vertex.

```
# calculate radius of LEC and add this information to vertices_spdf
vertices_spdf@data$radiusLEC <- rgeos::gDistance(sites,
                                                  vertices_spdf,
                                                  byid = TRUE) %>%
  apply(1, min)
```

Below you see a plot of the archaeological sites and the Voronoi diagram with their vertices. The problem of the border points becomes visible in this figure. They are created at the edge of the working area and thus do not reflect actual site distances.

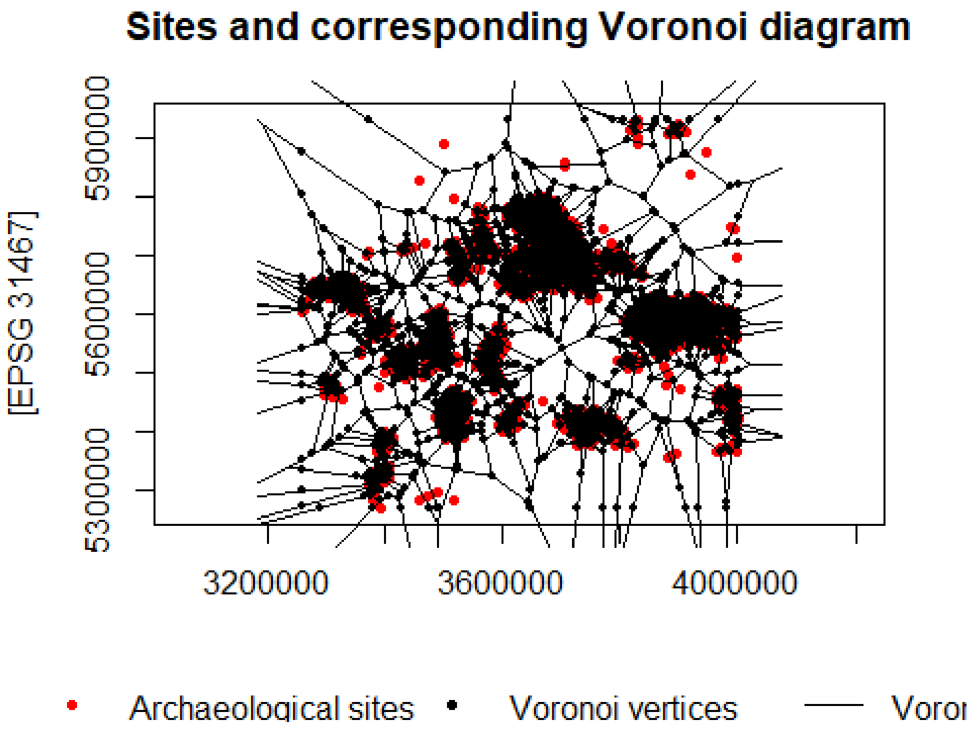

Kriging

The next part of this document will describe the used Kriging interpolation to estimate site distances on a regular spaced grid within the working area. These estimated site distances are a prerequisite for the creation of isolines.

Working step 6: Preparation and Grid

For kriging we need a grid of evenly distributed points. The interpolation algorithm will estimate the site distance (radius of LEC) at every point of the

grid, based upon the available vertices and their values for the radius of the LEC. A general introduction to kriging can be found at *Hengel (2007)* including kriging in R.
The grid spacing to be selected depends on various factors. The size of the working area or the regular distances between the archaeological sites are an important factor. In addition, the selected grid spacing will significantly influence the computing time required, because a shorter grid distance will create more points.
For the current example we will create a grid with a spacing of 1000 m between each point. This is a comparable coarse spacing but the working area is spanning most of Central Europe, which is a quite large area.

```
# Create a grid for kriging
grid <- expand.grid(x = seq(as.integer(range(vertices_spdf@coords[, 1]))[1],
                            as.integer(range(vertices_spdf@coords[, 1]))[2],
                            by = 1000),
                    y = seq(as.integer(range(vertices_spdf@coords[, 2]))[1],
                            as.integer(range(vertices_spdf@coords[, 2]))[2],
                            by = 1000)) %>%
  {sp::SpatialPoints(coords = .[1:2], proj4string =
sp::CRS("+init=epsg:31467"))}
```

The generated grid consists of 492528 points, at which the radius of the LEC is estimated by kriging.

Working step 7: Semivariogram

The kriging procedure requires a theoretical semivariogram, which is used to estimate the radii of the LEC at every point of the grid. To compute this theoretical semivariogram, we need to explore first the experimental semivariogram. At this point we will refer again to *Hengel (2007)* as we will give no introduction to kriging in general.
Before we can inspect the experimental semivariogram we have to define a lag distance. We divide the bounding box diagonal by 250, the default lag distance used in the Cologne Protocol:

```
# Define Bounding Box Diagonal
bbox_diag <- sp::spDists(t(vertices_spdf@bbox))[1, 2]

# Lagdistance = Bounding Box Diagonal / 250
lagdist <- bbox_diag/250
```

Now it is possible to compute the experimental semivariogram:

```
# Sample variogram
vertices_vario <- gstat::variogram(radiusLEC~1,
                                   vertices_spdf,
                                   width = lagdist)
```

```
## Registered S3 method overwritten by 'xts':
##   method     from
##   as.zoo.xts zoo
```

With the help of the experimental semivariogram, which you can see below, we are able to compute a theoretical semivariogram.

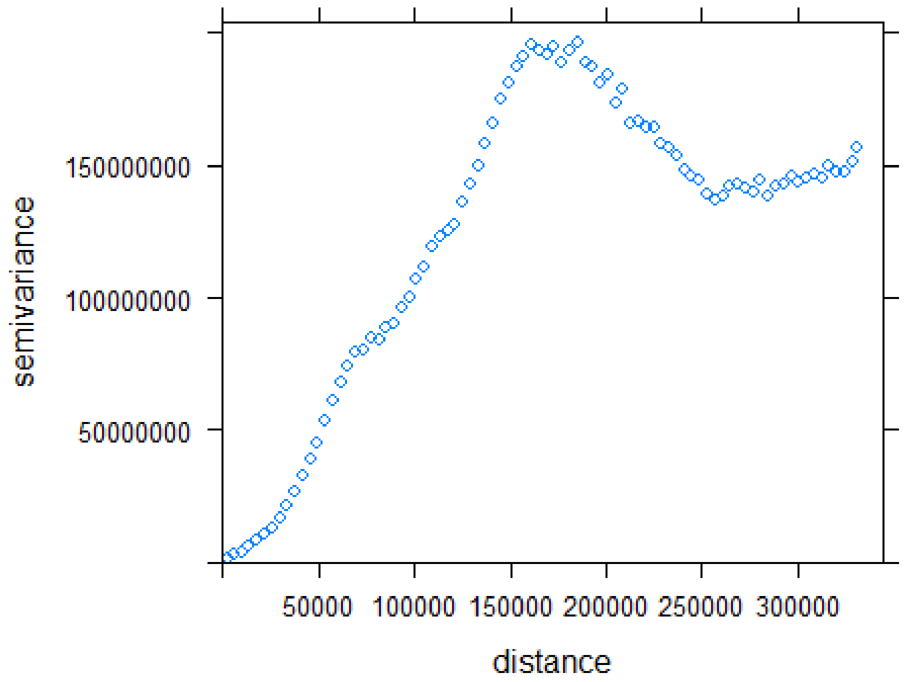

Doing so, we will need to identify the first plateau of the experimental semivariogram. According to *Zimmermann et al. (2004, 52)* the sill and range value of the theoretical semivariogram should be fitted to the first plateau of the experimental semivariogram. Additionally, the nugget value should be set to zero.
In the current example we will identify the first plateau automatically. Of course, it is also possible to choose these values by hand. And a decision has to be made on a case-by-case basis.

```
# Identify first plateau for fitting theoretical variogram
range.plateau <- vertices_vario %$%
  gamma %>%
  diff() %>%
  {vertices_vario[2][which.max(./.[1] < 0.1), ]}

sill.plateau <- vertices_vario$gamma[vertices_vario$dist == range.plateau]
```

Finally, we have to choose a model for the fitting of the theoretical semivariogram. The function `gstat::show.vgm()` will show you all available models. We, however, recommend the exponential “Exp” or spherical “Sph”

model.
In the current example we will use a spherical Model:

```
# Fitting theoretical variogram
vertices_vario_fit <- gstat::fit.variogram(vertices_vario,
                                           gstat::vgm(nugget = 0,
                                                      model  = "Sph",
                                                      psill  = sill.plateau,
                                                      range  = range.plateau),
                                           fit.sills = FALSE,
                                           fit.ranges = FALSE)
```

Comparing the experimental and the theoretical variogram shows that we more or less archived a good fit.

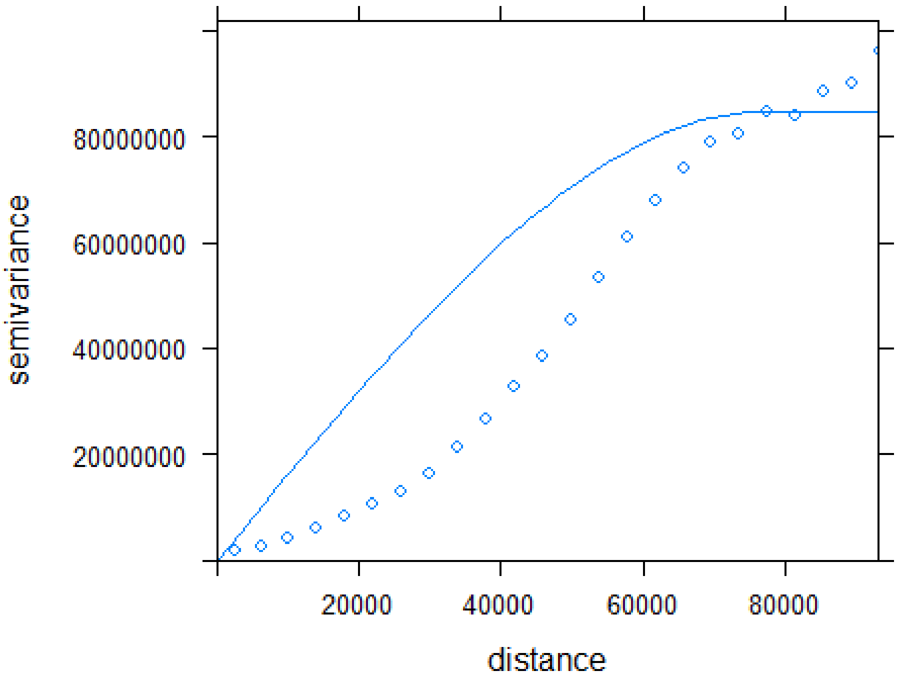

Kriging

Now we have every information for the kriging procedure. The only three variables we have to define are `nmin`, `nmax` and `maxdist` in the `gstat::krige()` function. `nmin` and `nmax` are in our case the minimum and maximum number of Voronoi vertices, which will be considered by the kriging algorithm, so we are using local Kriging. Furthermore, only observations (vertices) within a range of half of the bounding box diagonal will be used (`maxdist`). These are the default values for the Cologne Protocol (*cf. Schmidt et al. 2020*).

```
# Kriging
LEC_kriged <- gstat::krige(radiusLEC~1,
                           vertices_spdf,
                           grid,
```

```
                       model = vertices_vario_fit,
                       nmin = 3,
                       nmax = 10,
                       maxdist = bbox_diag/2,
                       debug.level = 0)
```

Working step 8: Kriging - inspect and export raster output

The results of the kriging procedure can be visualized by two plots. The first one shows the estimated radii of the LECs at every point of the created grid - the prediction:

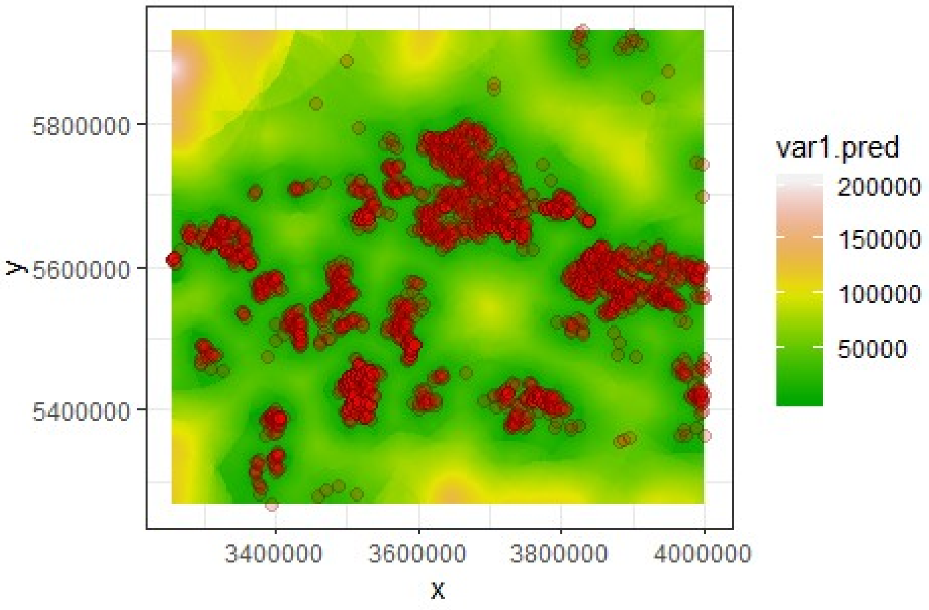

The second one shows the variance of the kriging results, which is a quality measure:

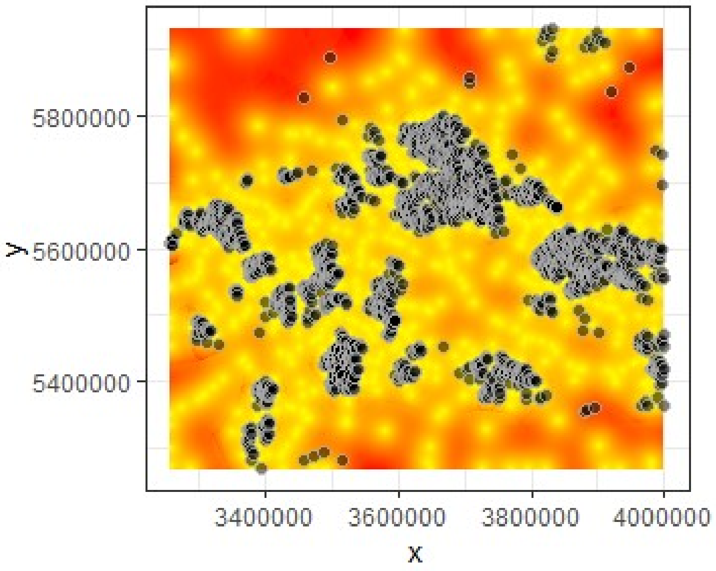

How to export the Kriging results as GeoTiff and grd-file:

```
# Write raster files as GeoTiff and grd-File for use in GIS-Programms like
QGIS

  # Kriging-Results
  r <- raster::rasterFromXYZ(data.frame(x = sp::coordinates(LEC_kriged)[,1],
                                        y = sp::coordinates(LEC_kriged)[,2],
                                        z = LEC_kriged$var1.pred),
                             crs = sp::CRS("+init=epsg:31467"))



  raster::writeRaster(r, "output/Kriging_raster.tif", format="GTiff",
overwrite=T)
  raster::writeRaster(r, "output/Kriging_raster.grd",format="raster",
overwrite=T)


  # Variance (Quality Measure)
  v <- raster::rasterFromXYZ(data.frame(x = sp::coordinates(LEC_kriged)[,1],
                                        y = sp::coordinates(LEC_kriged)[,2],
                                        z = LEC_kriged$var1.var),
                             crs = sp::CRS("+init=epsg:31467"))



  raster::writeRaster(v, "output/Variance_raster.tif", format="GTiff",
overwrite=T, prj=T)
  raster::writeRaster(v, "output/Variance_raster.grd",format="raster",
overwrite=T, prj=T)
```

Optimally Describing Isolines

On the basis of the kriging results we will create isolines and we will select an “Optimally Describing Isoline” (ODI). The selection of the ODI is based upon several statistical parameters of the isolines (*Zimmermann et al. (2004, 53-55)*).

Working step 9: Creating Contour Lines (Isolines)

In order to create isolines we need to transform the output of the `gstat::krige()` function, which is a raster, into `SpatialPolygonsDataFrame`. This has the advantage that statistics, like the number of archaeological sites in an isoline, can easily be calculated. One disadvantage is, that this transformation does take time.
To speed up this working step we transform the raster first into a `SpatialGridDataFrame` and afterwards use the function `inlmisc::Grid2Polygons()`:

```
isoline_polygons <- LEC_kriged %>%
  {raster::rasterFromXYZ(data.frame(x = sp::coordinates(.)[, 1],
                                    y = sp::coordinates(.)[, 2],
                                    z = .[[1]]),
                         crs = sites@proj4string)} %>%
  as("SpatialGridDataFrame") %>%
  inlmisc::Grid2Polygons(level = TRUE, at = seq(0, 20000, 500))

# This is not a reprojection!
sp::proj4string(isoline_polygons) <- sp::CRS("+init=epsg:31467")
```

In our example application isolines will be created starting at 0,5 km and ending up at 20 km with an equidistance of 0,5 km. When finished, we have to rename the isoline_polygons, because the function `inlmisc::Grid2Polygons()` does name them with reference to the mean value of each step, e.g. the isoline between 1500 m and 2000 m is named “1750” instead of “2000” in the current example.

```
# Rename the isolines because Grid2Polygon names them with the middle value
isoline_polygons@data[, 1] <- seq(0, 20000, 500)
[2:c(length(isoline_polygons@data[, 1])+1)]
```

Working step 10: Calculating the Area and Number of Sites per Isoline

In order to be able to select the ODI, it makes sense to look at the statistical properties of the isolines. These are, for example, the number of archaeological sites within the isolines, their difference in growth per equidistance, the number of distinct areas per isoline or the area increase per equidistance. In total, we will calculate eight different statistical properties and we will store them in a `data.frame`:

```
# Initialize data.frame
Isolines_stats <- data.frame(km_isoline = integer(),
                             number_Area = integer(),
                             number_Sites = integer(),
                             percent_Sites = integer(),
                             Area = integer(),
                             increase_Sites = integer(),
                             diff_Sites = integer(),
                             increase_Area = integer(),
                             diff_Area = integer(),
                             stringsAsFactors = FALSE)
```

What now follows is the code to fill the above mentioned `data.frame`. We will start with the number of distinct areas per equidistance:

```
# Counting the numbers of distinct areas per isoline
for (i in 1:length(isoline_polygons)) {
  Isolines_stats[i,2] <- length(isoline_polygons@polygons[[i]]@Polygons) -
    sum(sapply(isoline_polygons@polygons[[i]]@Polygons,
function(x) {sum(isTRUE(x@hole), na.rm = TRUE)}))
}
```

Now we will fill the `data.frame` with the name of the isolines:

```
# Insert name of isolines
Isolines_stats[, 1] <- isoline_polygons@data[, 1]
```

The next three calculations consider the number of sites within a LEC radius (certain site distance) and its percentage as well as the enclosed area:

```
# Calculate number of sites within a certain site distance
sites_n <- sapply(sp::over(isoline_polygons, sites, returnList = TRUE), nrow)
Isolines_stats$number_Sites <- cumsum(sites_n)

# Calculate the percentage increase in the nummber of site per isoline
Isolines_stats$percent_Sites <- (Isolines_stats[, 3] * 100) / length(sites)

# Calculate area enclosed by each isoline
iso_area <- raster::area(isoline_polygons)/1000000
Isolines_stats$Area <- cumsum(iso_area)
```

Based upon the statistical properties above, it is possible to calculate the increase in number of sites and area per equidistance:

```
# Calculate increase in numbers of sites per equidistance
Isolines_stats$increase_Sites <- c(NA, sites_n[-1])

# Calculate increase in area of polygon per equidistance
Isolines_stats$increase_Area <- c(NA, iso_area[-1])
```

Lastly, we will calculate the difference in increase of sites and area per equidistance:

```
# Calculate difference of increase of number of sites per equidistance
Isolines_stats$diff_Sites <- c(NA, diff(Isolines_stats[, 6]))
```

```
# Calculate difference in increase of area per equidistance
Isolines_stats$diff_Area <- c(NA, diff(Isolines_stats[, 8]))
```

To make reading of the `data.frame` easier, we will convert the equidistances into kilometers. If you use a different map unit you may have to change this step or leave it out:

```
Isolines_stats[, 1] <- Isolines_stats[, 1] / 1000
```

We can inspect the `data.frame`, although it is difficult to read the important information for the selection of the ODI. Hence it might be a good idea to visualize the information of the `data.frame`.

```
##    km_isoline number_Area number_Sites percent_Sites   Area increase_Sites
## 1         0.5          79          376      15.81161    108             NA
## 2         1.0         141          637      26.78722    374            261
## 3         1.5         259          643      27.03953    860              6
## 4         2.0         498          684      28.76367   2191             41
## 5         2.5         703          964      40.53827   6191            280
## 6         3.0         948         1393      58.57864  12558            429
## 7         3.5        1224         1700      71.48865  19847            307
## 8         4.0        1407         1927      81.03448  27226            227
## 9         4.5        1447         2028      85.28175  34486            101
## 10        5.0        1495         2092      87.97309  40962             64
## 11        5.5        1535         2146      90.24390  46981             54
## 12        6.0        1536         2189      92.05214  52572             43
## 13        6.5        1529         2219      93.31371  58010             30
## 14        7.0        1512         2247      94.49117  63189             28
## 15        7.5        1538         2265      95.24811  68277             18
## 16        8.0        1494         2284      96.04710  72929             19
## 17        8.5        1484         2299      96.67788  77415             15
## 18        9.0        1505         2307      97.01430  81948              8
## 19        9.5        1494         2315      97.35071  86168              8
## 20       10.0        1479         2321      97.60303  90172              6
## 21       10.5        1518         2326      97.81329  94218              5
## 22       11.0        1531         2332      98.06560  98234              6
## 23       11.5        1507         2334      98.14971 102200              2
## 24       12.0        1522         2340      98.40202 106063              6
## 25       12.5        1431         2341      98.44407 109599              1
## 26       13.0        1404         2342      98.48612 112963              1
## 27       13.5        1431         2344      98.57023 116303              2
## 28       14.0        1420         2346      98.65433 119477              2
## 29       14.5        1437         2346      98.65433 122793              0
## 30       15.0        1418         2347      98.69638 126101              1
## 31       15.5        1454         2347      98.69638 129271              0
## 32       16.0        1462         2347      98.69638 132288              0
## 33       16.5        1482         2348      98.73844 135385              1
## 34       17.0        1510         2348      98.73844 138529              0
## 35       17.5        1546         2348      98.73844 141655              0
## 36       18.0        1544         2348      98.73844 144915              0
## 37       18.5        1528         2351      98.86459 148160              3
## 38       19.0        1552         2351      98.86459 151450              0
## 39       19.5        1548         2352      98.90664 154748              1
## 40       20.0        1515         2355      99.03280 158135              3

##    diff_Sites increase_Area diff_Area
## 1          NA            NA        NA
## 2          NA           266        NA
## 3        -255           486       220
## 4          35          1331       845
```

```
## 5         239         4000      2669
## 6         149         6367      2367
## 7        -122         7289       922
## 8         -80         7379        90
## 9        -126         7260      -119
## 10        -37         6476      -784
## 11        -10         6019      -457
## 12        -11         5591      -428
## 13        -13         5438      -153
## 14         -2         5179      -259
## 15        -10         5088       -91
## 16          1         4652      -436
## 17         -4         4486      -166
## 18         -7         4533        47
## 19          0         4220      -313
## 20         -2         4004      -216
## 21         -1         4046        42
## 22          1         4016       -30
## 23         -4         3966       -50
## 24          4         3863      -103
## 25         -5         3536      -327
## 26          0         3364      -172
## 27          1         3340       -24
## 28          0         3174      -166
## 29         -2         3316       142
## 30          1         3308        -8
## 31         -1         3170      -138
## 32          0         3017      -153
## 33          1         3097        80
## 34         -1         3144        47
## 35          0         3126       -18
## 36          0         3260       134
## 37          3         3245       -15
## 38         -3         3290        45
## 39          1         3298         8
## 40          2         3387        89
```

Working step 11: Data export

Our script is able to export the isolines as polygons (.shp), the raster images of the kriging result and variance (.grd, GeoTiff) and a table with the statistical properties of the isolines. This code is not shown here, but you can find it at the end of the `03_Visualisation_Export.R` file (see also functions `write.table()` and `rgdal::writeOGR.R`).

Working step 12: Selecting the "Optimally Describing Isoline"

Following the recommendations of *Zimmermann et al. (2004, 53f.)* and *Zimmermann et al (2009, 9ff.)* we will have a look at three statistical properties: the difference in increase of sites per equidistance, the number of areas with a specific site density and the increase of included space. The most important is, however, the increase of included space (*Zimmermann et al. 2009, 9*). Please note that in the script a visualization of all statistical properties is provided.
To begin with, we will plot the difference in increase of sites per equidistance:

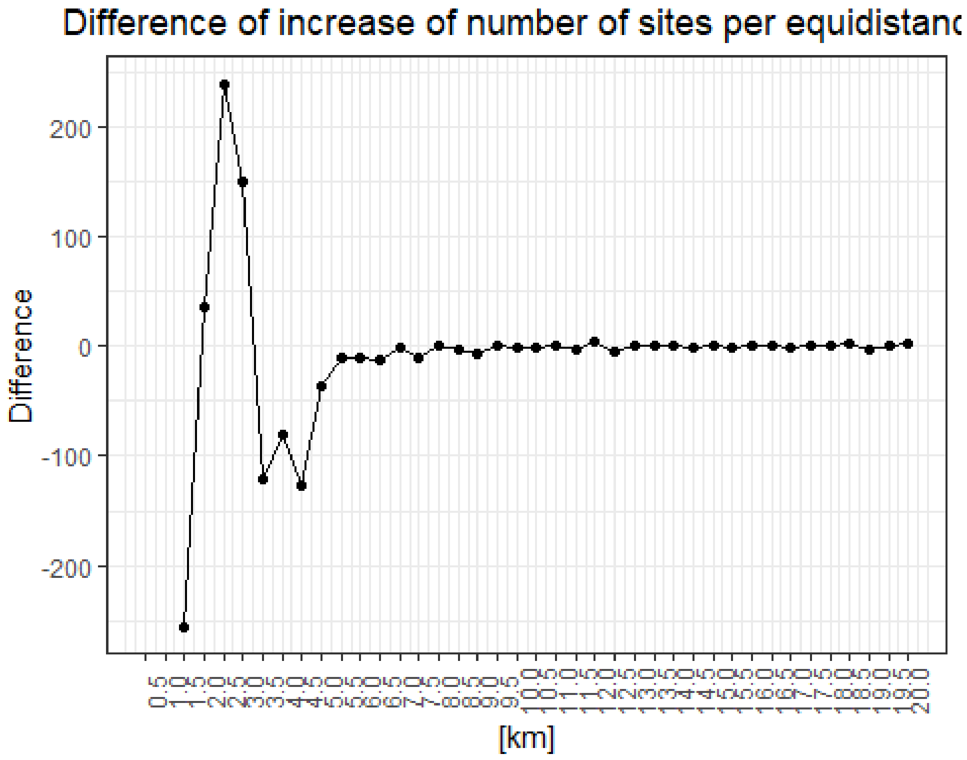

A maximum difference in increase of 239 sites can be seen from the 2.0 to 2.5 km isoline.
Next we will have a look at the number of areas with a specific site density:

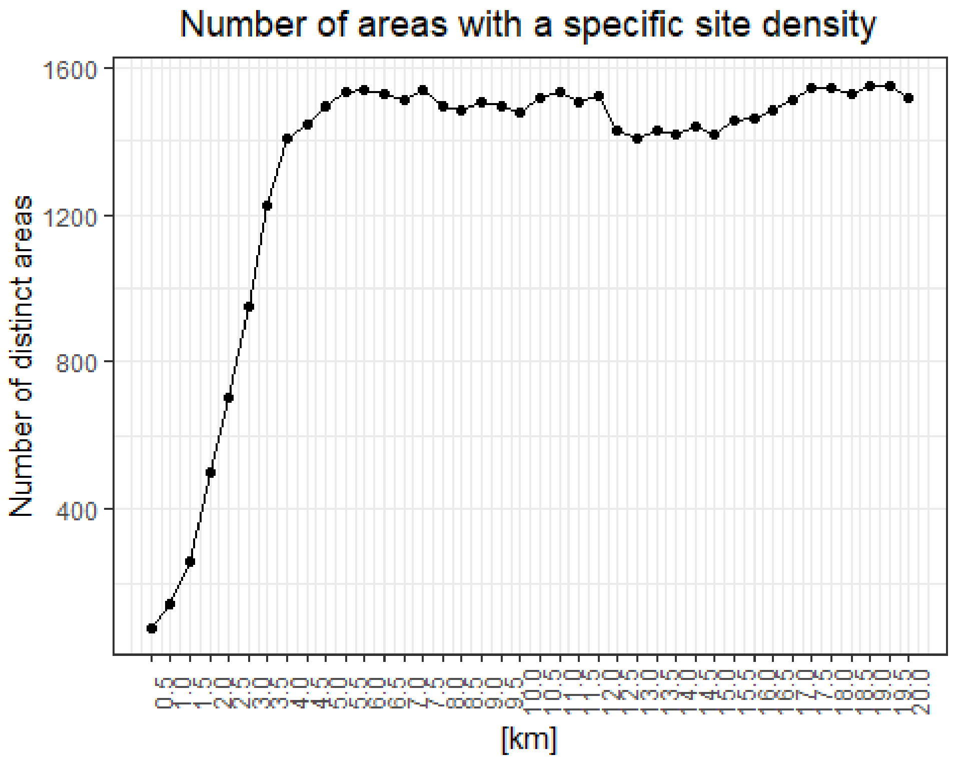

The number of areas with a specific site density reach a plateau at the 5.0 or 5.5 km isoline. Lastly, we will plot the most important statistical parameter: the increase of included space.

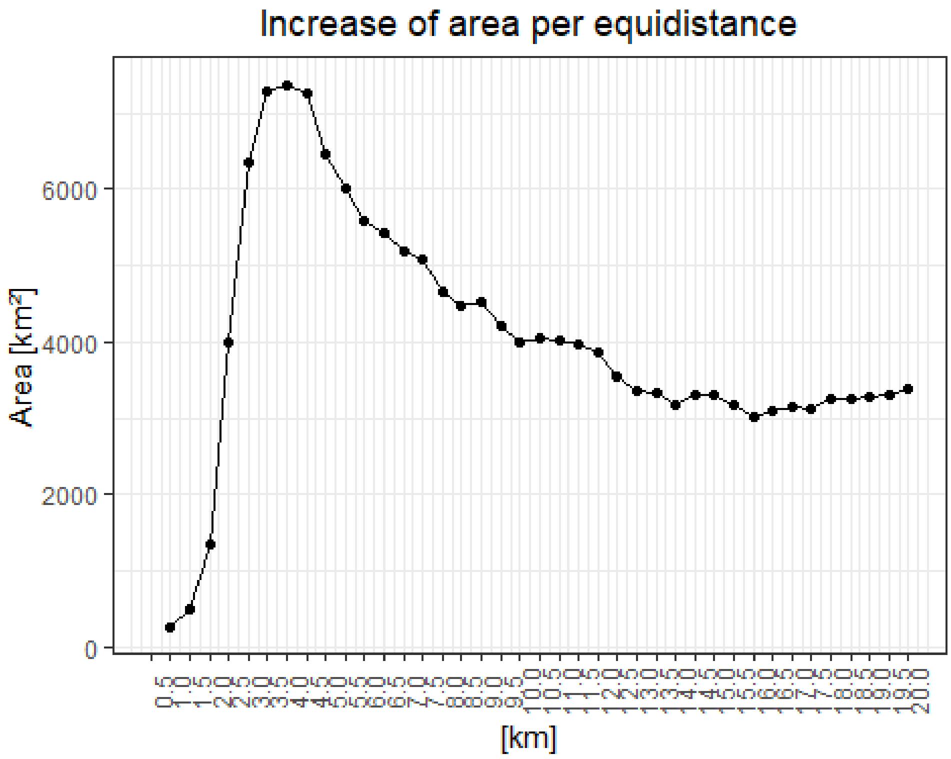

The figure shows a maximum of included space at the 4.0 km isoline. According to *Zimmermann et al. 2009, 9ff.* this isoline **would be selected as ODI**. For a theoretical background we refer to the mentioned publications.
We would like to note that the ODI **should not be selected** automatically based upon a maximum value of included space. For example several archaeological cultures have shown two maxima which need an archaeological interpretation (e.g. patterns of fission and fusion in hunter/gatherer societies (*cf. Kretschmer et al. 2016; Lundström et al. 2020*)). Furthermore in some cases it is not possible to observe a maximum; instead, with increasing distance between sites a continuous increase of space is to be observed. This would indicate a data set indicating a poor archaeological record (*cf. Zimmermann et al. 2009, 10*).

Bibliography

Hengel, T., **2007**. A Practical Guide to Geostatistical Mapping of Environmental Variables. (Luxembourg 2007).

Kretschmer, I., Maier, A., Schmidt, I., **2016**. Probleme und mögliche Lösungen bei der Schätzung von Bevölkerungsdichten im Paläolithikum. In:

Kerig, T., K. Nowak, G. Roth (Eds.), Alles was zählt. Festschrift für Andreas Zimmermann. Universitätsforschungen zur prähistorischen Archäologie 285, Bonn: Habelt: 47-57.

Lundström, V., Peters, R., Riede, F., **2020**. Demographic estimates from the Palaeolithic-Mesolithic boundary in Scandinavia: comparative benchmarks and novel insights. *Philosophical Transactions of the Royal Society B.*

Preuss, J. (ed.), **1998**. Das Neolithikum in Mitteleuropa: Kulturen, Wirtschaft, Umwelt vom 6. bis 3. Jahrtausend v. u. Z., Übersichten zum Stand der Forschung. Beier & Beran: Weissbach.

Schmidt, I., Hilpert, J., Kretschmer, I., Peters, R., Broich, M., Schiesberg, S., Vogels, O., Wendt, K. P., Zimmermann, A., Maier, A., **2020**. Approaching Prehistoric Demography: Proxies, Scales and Scope of the Cologne Protocol in European contexts. *Philosophical Transactions of the Royal Society B.*

Zimmermann, A., Richter, J., Frank, T., Wendt, K.P., **2004**. Landschaftsarchäologie II. Überlegungen zu Prinzipien einer Landschaftsarchäologie. *Bericht der Römisch-Germanischen Kommission* 85, 37-96.

Zimmermann, A., Wendt, K.P., Frank, T., Hilpert, J., **2009**. Landscape Archaeology in Central Europe. *Proceedings of the Prehistoric Society* 75, 1-53.
